# Ultrasound neural interface enabling acute spinal cord monitoring and blood flow modulation

**DOI:** 10.1126/sciadv.aeg2342

**Published:** 2026-11-04

**Authors:** Max J. Kerensky, Siddharth Krishnan, Ruixing Liang, Kelley M. Kempski Leadingham, Victor M. Quiroz, Joshua Punnoose, Constantin Smit, Denis Routkevitch, Avisha Kumar, A. Daniel Davidar, Leonard Steger, Carly Weber-Levine, Kelly Jiang, Meghana Bhimreddy, Stuart J. Bauer, Angelica F. Lopez, Patrick Kramer, Kayla Robinson, Arjun K. Menta, Abdul Karim Ghaith, Andrew M. Hersh, Haley G. Abramson, Ali Mohammadabadi, John N. Theodore, Osamah M. Zmily, Ritvik Jillala, Vikas Vattipally, David Kopala, Siavash S. Barough, Mohammad Hossein Amini, Jonathan Maltz, Francisco Chavez, Kyle Morrison, Laura Roy, Ian Suk, Ryan P. Lee, Betty Tyler, Mark Luciano, Nitish V. Thakor, Joshua C. Doloff, Nicholas Theodore, Amir Manbachi

**Affiliations:** ^1^ Department of Biomedical Engineering, Johns Hopkins University, Baltimore, MD, USA.; ^2^ HEPIUS Innovation Laboratory, School of Medicine, Johns Hopkins University, Baltimore, MD, USA.; ^3^ Department of Neurosurgery, Johns Hopkins University, Baltimore, MD, USA.; ^4^ Department of Electrical & Computer Engineering, Johns Hopkins University, Baltimore, MD, USA.; ^5^ Translational Therapeutics & Regenerative Engineering Center, Johns Hopkins University, Baltimore, MD, USA.; ^6^ Department of Mechanical Engineering, Johns Hopkins University, Baltimore, MD, USA.; ^7^ Advanced Patient Monitoring, Becton, Dickinson, and Company, Irvine, CA, USA.; ^8^ Department of Neurology, Johns Hopkins University, Baltimore, MD, USA.; ^9^ Fujifilm Visualsonics Inc., Toronto, ON, Canada.; ^10^ Molecular Biophysics and Integrated Bioimaging, Berkeley Lab, University of California, Berkeley, CA, USA.; ^11^ Sonic Concepts Inc., Bothell, WA, USA.; ^12^ Roy Illustration, New York, NY, USA.; ^13^ Department of Materials Science and Engineering, Institute for NanoBioTechnology, Johns Hopkins University, Baltimore, MD, USA.; ^14^ Department of Orthopaedic Surgery, School of Medicine, Johns Hopkins University, Baltimore, MD, USA.; ^15^ Department of Pediatrics, School of Medicine, Johns Hopkins University, Baltimore, MD, USA.; ^16^ Department of Anesthesiology and Critical Care, School of Medicine, Johns Hopkins University, Baltimore, MD, USA.

## Abstract

The days following decompression surgery for acute spinal cord injury (SCI) are a critical period for postoperative monitoring. After wound closure, clinicians lack on-demand access to local spinal cord morphology and hemodynamics, and care therefore remains guided largely by systemic surrogates rather than direct postoperative insight into the injured cord. We present the Multimodal Ultrasound System for Injury Care (MUSIC), a temporary epidural ultrasound neural interface placed during standard decompression surgery to enable postoperative brightness-mode imaging, Doppler-derived hemodynamic monitoring, and future device-integrated ultrasound actuation. Across simulations, benchtop, human cadaveric, and porcine studies, MUSIC enabled on-demand visualization of cord structure, hematoma tracking, detection and longitudinal tracking of localized Doppler flow signatures attributable to the sulcal artery region, and, in healthy pigs, proof-of-concept focused ultrasound modulation of blood-flow velocity. MUSIC establishes a temporary postoperative acoustic interface that provides spinal cord information to inform monitoring, future management, and localized intervention strategies after SCI.

## INTRODUCTION

Spinal cord injury (SCI) is a devastating condition that affects approximately 500,000 individuals each year (*1*). Neurologic deterioration after the initial mechanical insult may be influenced by secondary mechanisms such as ischemia, inflammation, edema, excitotoxicity, and microvascular disruption that evolve over the first hours to days (*2*, *3*). Postoperative management focuses on maintaining cord perfusion and blood flow in the early phase, a time marked by hemodynamic instability, evolving hematoma formation, and tissue vulnerability.

Earlier guidelines recommended mean arterial pressure (MAP) augmentation to 85 to 90 mmHg for 7 days after acute SCI, whereas recent guidelines suggest a lower MAP limit of 75 to 80 mmHg for 3 to 7 days and emphasize that supporting evidence remains limited (*4*–*6*). A recent multicenter randomized trial comparing augmented (>85 to 90 mmHg) and conventional (>65 to 70 mmHg) MAP targets did not identify improved 6-month neurologic recovery and reported higher respiratory complications, longer mechanical ventilation, and worse organ dysfunction in the augmented group (*7*). These data underscore that systemic MAP remains a global surrogate rather than a direct measure of regional spinal cord microvascular physiology. This limitation leaves clinicians without a technology to measure local spinal cord hemodynamics at the bedside. As a result, care relies on systemic manipulation, neurologic examinations, and intermittent imaging such as magnetic resonance imaging (MRI), which cannot provide continuous insight into the tissue at highest risk. Sustained systemic blood pressure augmentation also carries risks including cardiovascular strain, pulmonary complications, and extremity- or end-organ perfusion complications, underscoring the need for methods that complement systemic targets with local physiologic information (*6*–*8*).

Following routine decompression of the spinal cord and stabilization of the spine, there is no existing technology that enables real-time, localized monitoring of spinal cord hemodynamics. This shortfall allows critical postoperative events such as microvascular breakdown, venous congestion, edema progression, or enlarging hematomas to proceed undetected until symptoms appear. Intraoperative ultrasound has demonstrated anatomical and functional spinal cord monitoring capability (*9*–*12*), but conventional probes remain too bulky for continuous use, lack access through the closed surgical field, and are not designed for longitudinal monitoring (*9*–*15*).

Imaging alone incompletely addresses the problem of evolving tissue compromise due to secondary injury processes. Effective postoperative care after SCI also requires precise, localized interventions to restore or stabilize regional blood flow. Focused ultrasound (FUS) offers promise in vascular modulation in the central nervous system (*16*–*23*), yet no existing platform supports FUS delivery directly to the spinal cord in the immediate postoperative period. Recent advances in wearable, implantable, and conformable ultrasound technologies have demonstrated that sustained, device-based physiological monitoring is becoming technically feasible across many organ systems (*24*–*31*), making this an opportune moment to extend these innovations to the spinal cord.

To meet these needs, we developed the Multimodal Ultrasound System for Injury Care (MUSIC), a temporary epidural neural interface placed during standard decompressive surgery. The device geometry and flexibility are inspired by clinically familiar epidural devices placed on or adjacent to the dura; this comparison is limited to form factor and procedural workflow rather than a claim of equivalent safety. MUSIC integrates brightness-mode (B-mode) imaging, Doppler monitoring, and FUS delivery to provide on-demand assessment of spinal cord morphology and Doppler-derived blood-flow velocity (BFV) during the acute postoperative window. Through simulations, benchtop characterization, human cadaveric placement, and porcine studies, we demonstrate that MUSIC can monitor spinal cord morphology, identify hematoma formation, detect and longitudinally track localized Doppler flow signatures attributable to the sulcal artery region, and, in healthy pigs, modulate Doppler-derived BFV using device-delivered FUS. MUSIC is therefore positioned as a temporary postoperative acoustic interface for local monitoring and proof-of-concept hemodynamic modulation rather than as a proven therapeutic intervention.

## RESULTS

### MUSIC’s ultrasound capabilities

The MUSIC platform is an ultrasound device for postoperative SCI monitoring following decompressive surgery (Fig. 1, A and B). It combines on-demand monitoring with FUS-delivery capability. Three imaging arrays track tissue changes, while two FUS arrays provide a potential pathway for localized ultrasound actuation (Fig. 1C). The first two ultrasound imaging arrays capture different planes at or near the injury site, and the third ultrasound imaging array is designed to capture an adjacent reference region. Using four imaging modalities [B-mode, spectral Doppler, color Doppler (shown later), and power Doppler], MUSIC can yield data on tissue morphology, hematoma progression, and Doppler-derived microvascular BFV after the incision is closed (Fig. 1D). Leveraging its imaging capabilities, MUSIC can also deliver low-intensity FUS as a future route for localized hemodynamic modulation (Fig. 1E).

**Fig. 1.**
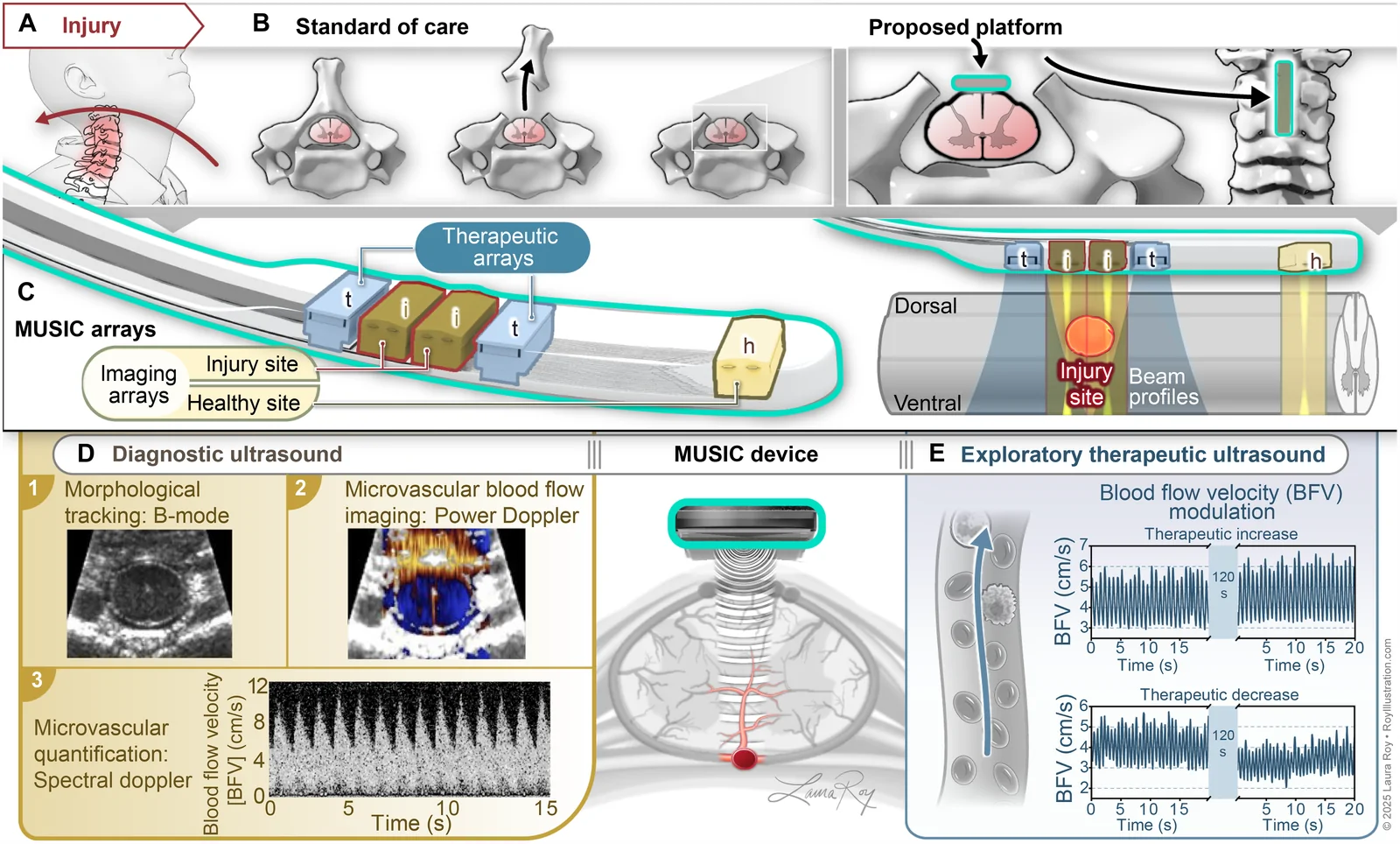
Overview of the implanted ultrasound spinal cord platform. (**A**) Depiction of a traumatic SCI. (**B**) The MUSIC device is designed to be placed on the dorsal side of the injured spinal cord during standard-of-care decompression surgery for SCI. (**C**) Design of the three imaging and two FUS arrays enables monitoring and informed ultrasound-delivery options. (**D**) Diagnostic capabilities empower clinicians and researchers to monitor patients with SCI. (**E**) Doppler-derived spinal cord BFV can be modulated by FUS in proof-of-concept experiments.

### Hardware design and characterizations of the ultrasound device

The MUSIC hardware features a compact design, 45 mm long × 10 mm wide × 3 mm thick, housing diagnostic and FUS piezoelectric ultrasound arrays attached to a flexible printed circuit board substrate (Fig. 2A and movie S1). The two FUS arrays were designed to have a center frequency of 2.5 MHz, as validated by an impedance analyzer (Fig. 2B). The corresponding normalized pressure field is shown (Fig. 2C). The three imaging arrays of the MUSIC device can transmit at different center frequencies to balance the trade-off between axial resolution (10.5 MHz: 326 ± 68 μm; 16 MHz: 289 ± 29 μm) and penetration depth (10.5 MHz: 30 mm; 16 MHz: 20 mm) needed for monitoring SCI (Fig. 2, D and E).

**Fig. 2.**
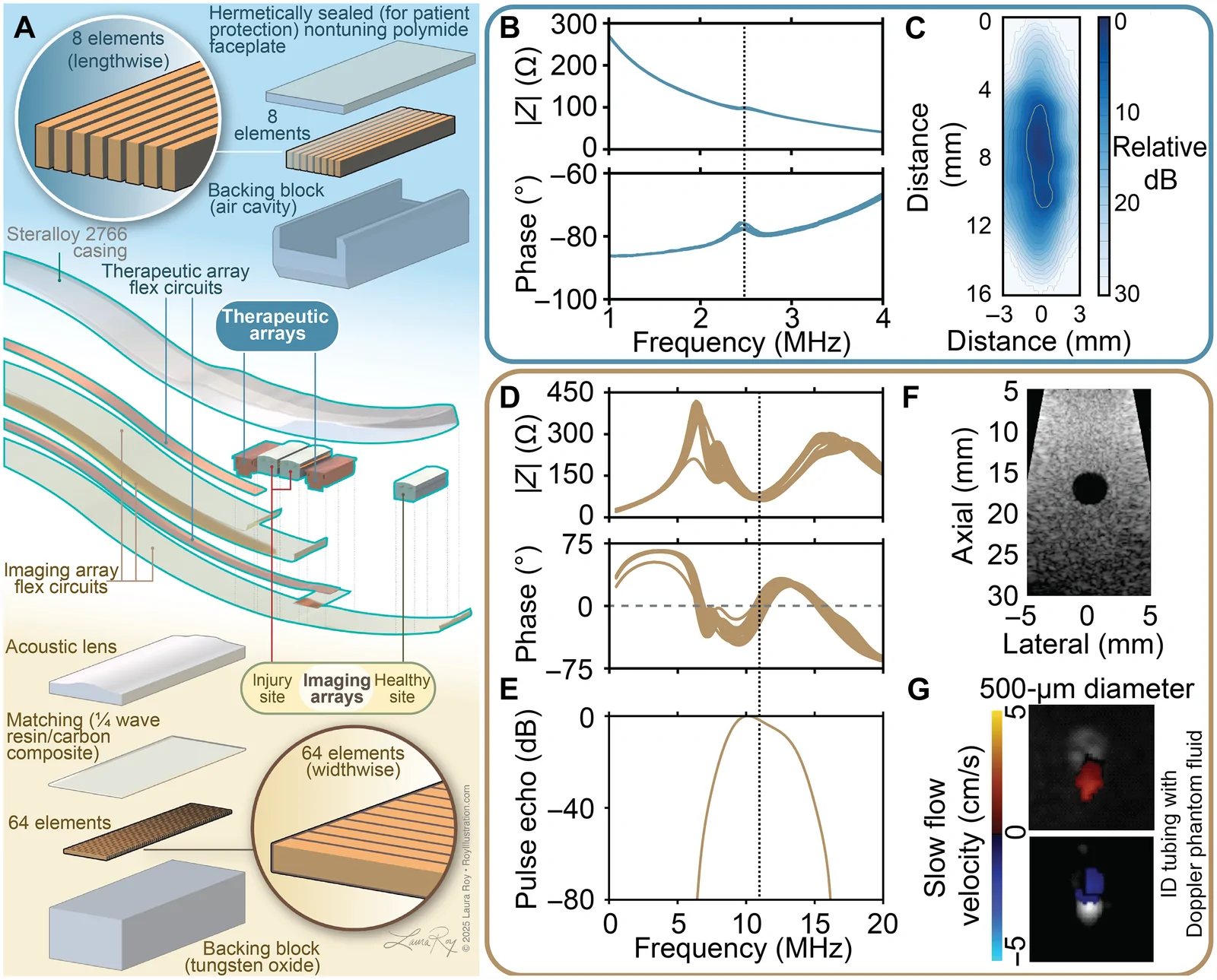
Components, characterizations, and benchtop deployment of MUSIC. (**A**) Exploded view of the core device components. (**B**) Experimental impedance and phase plots for the FUS arrays, which are designed to operate near 2.5 MHz. (**C**) Demonstrating deployment-ready functionality, an unfocused plane wave from eight FUS elements was characterized in a water bath, and a normalized cross-sectional pressure field is shown. (**D**) A representative characterization of imaging arrays with the matching network. The minima on the impedance plot and zero-crossing on the phase plot indicate reliable operation at 10.5 MHz. (**E**) The pulse echo of the imaging array reveals a 31.5% bandwidth. (**F**) B-mode ultrasound resolves an anechoic region in a benchtop ultrasound phantom (model: 040GSE, CIRS). (**G**) Zoomed-in snapshots of color Doppler ultrasound detect slow flow in 500-μm diameter tubing filled with Doppler fluid (model: 769DF, CIRS).

The device delivers high spatiotemporal ultrasound imaging resolution despite the constraints of its compact design (*32*). We tailored acoustic lenses to mechanically focus the acoustic beam, enhancing spatial resolution and signal-to-noise ratio (SNR) while minimizing ultrasound exposure to surrounding tissues (Fig. 2A). To address both the constraints of compactness and the challenges it creates, we offloaded components outside the body. A detachable breakout box was designed to interface MUSIC with external processing units through a matching network, reducing implanted circuitry and minimizing volume (section S1 and fig. S1). Last, to mitigate the electromagnetic interference and especially its radiofrequency (rf) component, associated with flexible circuits, a polyimide dielectric shielding layer was added (*33*).

A wired design was selected for this first-generation temporary platform for two primary reasons. First, it supports reliable signal transmission while reducing implanted electronics, maintaining physical access to the device, and enabling controlled retrieval. Second, MUSIC is intended for acute postoperative intensive care unit (ICU) patients, who are typically bed bound and already managed with externalized monitoring lines, drains, and support devices. In this context, the externalized flex cable is designed to exit through a tunneled pathway and route flat against the body, analogous to temporary spinal or lumbar drainage systems. This low-profile, flexible architecture provides strain relief and reduces exposed cable loops that could be caught or pulled during routine patient repositioning. In clinical use, the cable exit site would be managed with sterile occlusive dressings and standard infection-control practices for temporary externalized neurosurgical devices. Future versions may reduce tethering burden further through integrated release mechanisms, miniaturized electronics, or wireless architectures.

The acoustic beam profiles from the FUS arrays were characterized in a water tank (Fig. 2C) (*34*). The maximum measured peak rarefactional pressure was 0.40 MPa, corresponding to a mechanical index (MI) of 0.26 and a maximum spatial peak pulse average intensity (*I*_SPPA_) of 5.41 W/cm^2^. These values are reported to contextualize the acoustic exposure delivered by MUSIC (*35*–*37*). Cavitation risk depends on the complete exposure condition, including pulse duration, frequency, waveform, propagation medium, tissue composition, endogenous nuclei or exogenous microbubbles, standing waves, and boundary conditions. Accordingly, these values are presented as exposure context for the tested conditions rather than as a universal cavitation threshold or definitive proof of clinical safety. To assess B-mode image quality, we used the CIRS Multi-Purpose Multi-Tissue Ultrasound Phantom (model 040GSE, Sun Nuclear, FL, USA) (Fig. 2F and section S2). The contrast and generalized contrast-to-noise ratio (gCNR) of an anechoic cyst were −16.7 dB and 0.93, respectively, indicating sufficient visibility and detectability with MUSIC. The SNR was 4.94, and the field of view was 30 mm deep by 15 mm laterally. MUSIC achieved lateral full width at half maximum (FWHM) values of 438 ± 35 μm at 10.5 MHz and 342 ± 29 μm at 16 MHz, comparable to commercially available transducers under the tested phantom conditions (section S2 and fig. S2). To evaluate color Doppler performance, we used a slow-flow 500-μm diameter tubing phantom filled with Doppler fluid (model 769DF, Sun Nuclear, FL, USA) (Fig. 2G).

### Validation of on-demand, real-time imaging capabilities

Leveraging our custom-designed MUSIC device for next-generation monitoring approaches, we explored a range of local health-tracking capabilities. Because of the small size of MUSIC, comparable to a spinal cord stimulator or a Jackson-Pratt style drain (section S3 and fig. S3), the device can be integrated into the workflow of a standard laminectomy (Fig. 3A). Following a laminectomy, monitoring microvascular BFV could provide a useful local hemodynamic marker for additional studies and future interventions (*38*, *39*). Of particular relevance is the sulcal artery region, supplied by branches of the anterior spinal artery, which supports the ventral spinal cord; reported sulcal artery dimensions vary by species, branch order, fixation state, anatomy, and measurement method, with expected diameters generally on the order of ∼0.1 to 0.4 mm, with 0.140 serving as our guiding approximate (*40*). MUSIC does not directly geometrically resolve the sulcal artery lumen; rather, MUSIC detects and tracks a localized Doppler flow signature arising from a spectral Doppler sample volume containing or overlapping the sulcal artery region. This approach enables longitudinal tracking of Doppler-derived BFV from an extradural position within the rail system without requiring direct lumen-level microvascular resolution. The successful insertion of the rail system over a cadaveric spinal cord supports feasibility of placement in human anatomy (section S4 and figs. S5 to S7).

**Fig. 3.**
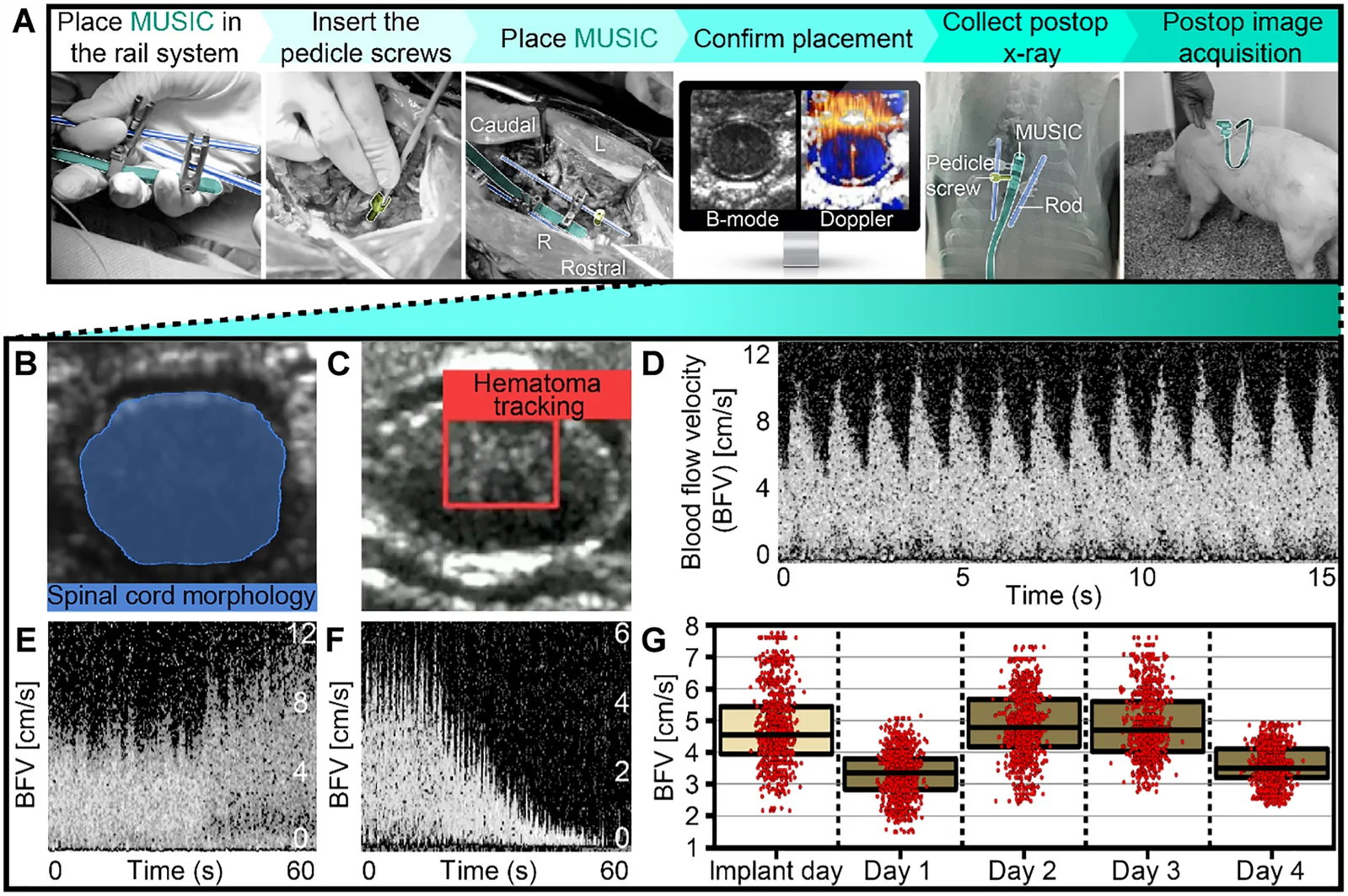
Implantation and postoperative imaging capabilities of MUSIC. (**A**) The surgical workflow for the ultrasound neural interface is depicted with photographs of different pigs. B-mode and power Doppler images from MUSIC are used to confirm placement. The representative B-mode and power Doppler images shown in Fig. 3A are duplicates of those presented in Fig. 1D. (**B**) Data are compatible with external software, including segmentation models [Segment Anything Model 2 (SAM 2), Meta FAIR] (*42*). (**C**) A custom localization pipeline automatically creates a bounding box around hematomas when present. (**D**) Quantifying spinal cord health through BFV over time, spectral Doppler detects a localized Doppler flow signature within a spectral Doppler sample volume anatomically attributable to the sulcal artery region. MUSIC does not directly resolve the sulcal artery lumen or wall. (**E** and **F**) Increasing and decreasing microvascular BFV can be observed with the device. For visualization purposes, the dynamic ranges and brightness of the images are adjusted. (**G**) The ability to measure BFV changes daily on demand during the critical postoperative period is shown. Sample 5-s windows are plotted with 25th, 50th, and 75th percentiles marked in black, excluding data points beyond a 1.5 outlier coefficient for each recording. The full spectral Doppler envelope is plotted with red dots. This stream of data provides a means to longitudinally assess Doppler-derived BFV at and around the injury site (dark brown) and at the reference-array site (light brown) in parallel.

In addition to color Doppler, MUSIC imaging supports triplex mode, combining B-mode, power Doppler, and spectral Doppler for relevant SCI tracking, including hemodynamic monitoring. This capability enables real-time processing for both intraoperative and postoperative applications, providing diagnostic information and guidance for potential future interventions. We demonstrated this capability in vivo by implanting MUSIC over the porcine spinal cord after thoracic laminectomy. MUSIC visualized spinal cord anatomy, with differentiation of the dura, intrathecal space, and central canal, and detected a localized Doppler flow signature attributable to the sulcal artery region (Fig. 3A). Furthermore, MUSIC quantified Doppler-derived spinal cord BFV through spectral Doppler, with clear visibility of cardiac cycles (Fig. 3D). MUSIC was able to identify increasing BFV, as well as decreasing BFV to 0 during euthanasia (Fig. 3, E and F).

While prolonged monitoring is desirable, it can produce large volumes of data that may be challenging for radiologists or sonographers to manage. To address this potential problem, we evaluated deep learning–based analysis adjuncts using YOLOv8 to detect and track a hematoma in the spinal cord when present, with a mean average precision (mAP@50) of 99% in held-out images from the tested cohort (Fig. 3C, movie S2, section S5, and fig. S8) (*41*). Moreover, the spinal cord can be segmented without training using Segment Anything Model 2 (SAM 2; Meta FAIR) (Fig. 3B), illustrating how MUSIC data streams can be incorporated into external analysis pipelines (*42*).

We first detected and quantified Doppler-derived spinal cord BFV intraoperatively, demonstrating MUSIC’s capacity to assess local hemodynamics in real time (movie S3). Postoperatively, we evaluated the feasibility of monitoring BFV during the critical period following SCI. Specifically, we investigated MUSIC’s potential application in the ICU by performing daily BFV assessments after injury, with recordings in the porcine model conducted while animals were freely moving and eating, presenting greater imaging challenges than would be expected in immobilized ICU patients. This provided quantifiable local hemodynamic data during the critical postoperative window, when timely interventions could mitigate secondary injury (Fig. 3G) (*43*). In addition, we conducted an injury-free postoperative study as a reference. Consistent with previous MRI studies in humans undergoing laminectomies, we observed changes in the spinal cord’s shape during the survival period (*44*). End-point CT myelograms showed no substantial cord compression (section S6). MUSIC was positioned at a distance from the dura and secured in place using our custom rail system (Fig. 3A). By enabling reproducible BFV recordings (Fig. 4), MUSIC offers researchers and clinicians a tool for on-demand assessment of spinal cord health following injury.

**Fig. 4.**
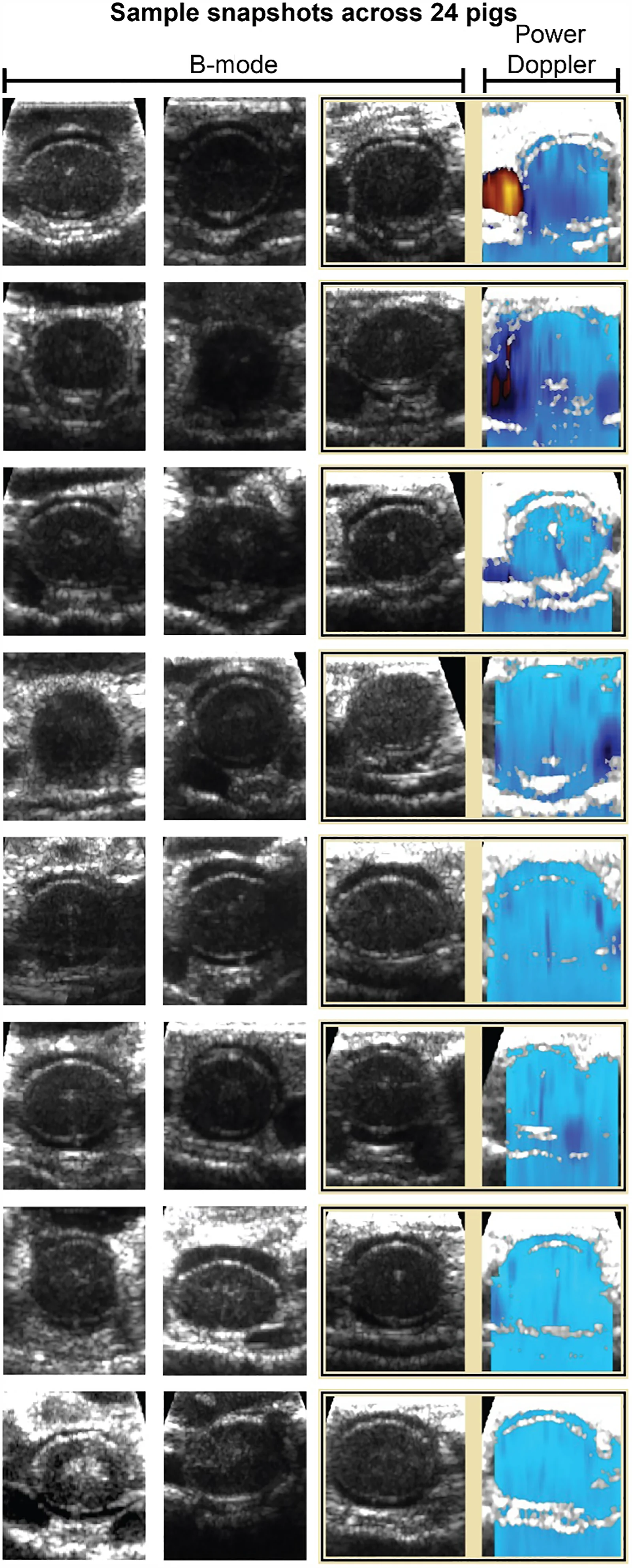
Robust sensor deployment across subjects. A representative imaging grid depicts the utility of MUSIC across pigs. B-mode (*N* = 24) and power Doppler images (*N* = 8) span healthy and injured states and both intraoperative and implantable settings and include both slow and fast flow.

### Feasibility of MUSIC-enabled FUS to modulate spinal cord blood flow velocity

Prolonged ischemia can lead to neuronal and glial cell death, exacerbating the primary injury, especially in the acute phase of SCI (*45*–*47*). BFV is a useful Doppler-derived marker of local hemodynamics, but it is not equivalent to tissue perfusion, vasodilation, vessel diameter, or volumetric blood flow. Volumetric flow depends on vessel cross-sectional area, which was not measured in this study (section S7 and fig. S9) (*48*–*50*). Accordingly, the following experiments are interpreted as proof-of-concept modulation of Doppler-derived BFV in healthy pigs rather than as evidence of improved spinal cord perfusion or therapeutic efficacy after SCI.

FUS can perform neuromodulation and open the blood-brain and blood–spinal cord barriers (*21*, *22*, *51*, *52*). Here, we focused on ultrasound’s ability to modulate spinal cord hemodynamics, a component of SCI recovery biology that requires careful local measurement (*53*, *54*). This approach to BFV modulation could be promising as a future mechanistic and therapeutic-study tool because the vascular monitoring capabilities of MUSIC could support sensing-informed sequential intervention after further validation.

Assessing the feasibility of MUSIC to induce hemodynamic modulation with FUS, we recorded the BFV response with a commercial ultrasound transducer in a healthy porcine model (Fig. 5A). In the absence of established parameters for spinal cord hemodynamic modulation in the literature, we performed parameter sweeps in our in vivo porcine model, varying input voltage, pulse repetition frequency (PRF), duty cycle, and sonication duration (section S8 and figs. S10 to S12). Positional overlap between the localized Doppler signal of interest and MUSIC-delivered FUS was assessed by the presence of an overlapping harmonic pattern in the spectrogram during concurrent FUS and imaging (section S8 and fig. S11).

**Fig. 5.**
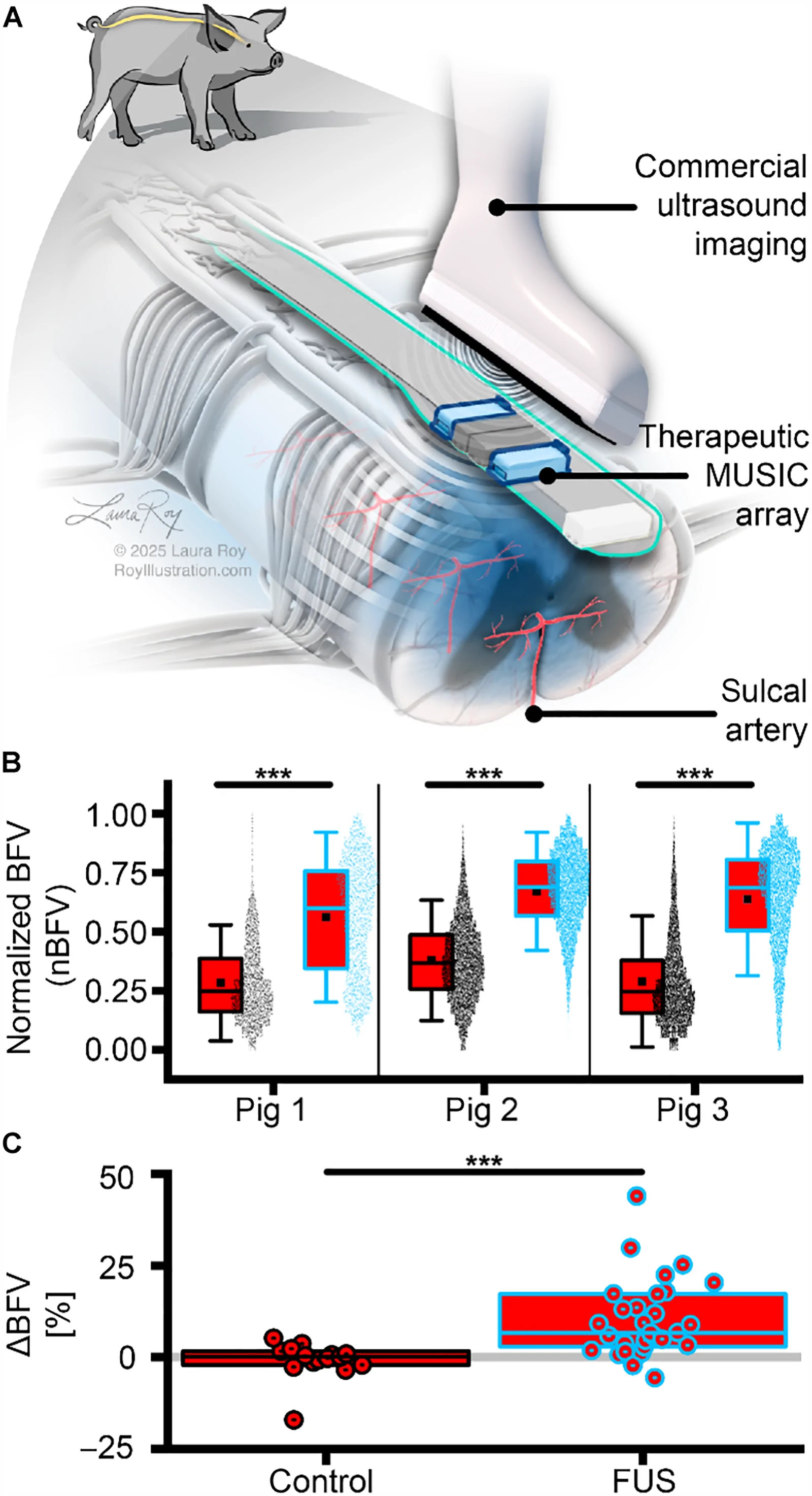
Exploration of potential local BFV modulation with MUSIC. (**A**) Isolating the FUS elements of the MUSIC device, exploratory effects of FUS on microvascular BFV were recorded with an adjacent commercial ultrasound transducer along the dorsal spinal cord in a porcine model. The commercial system records BFV with spectral Doppler and supports positional overlap assessment during FUS delivery. (**B**) Session-level BFV changes during FUS across 30 repeated sessions in three pigs (*n* = 4, *n* = 13, and *n* = 13). The second and third quartiles of session-normalized BFV (nBFV) are pooled and shown with ±1.5 SD whiskers (***P < 0.001). Pre-FUS data are in black, and post-FUS data are in blue. (**C**) The relative change in BFV in control sessions (black; *n* = 15) is compared with FUS sessions (blue; *n* = 30) (***P < 0.001). The BFV analyses use FUS-off pre/post windows specified for each analysis, including 20-s sampling windows for the nBFV comparison and expanded 30-s windows for the corresponding control, vital-sign, and RI summaries where indicated. These session-level statistics are exploratory repeated-observation analyses and are not interpreted as independent animal-level therapeutic efficacy. Additional graphics on methods, dosing, and parameters can be found in section S8.

To avoid the FUS-induced harmonic pattern captured by the commercial ultrasound spectrogram, we evaluated FUS-associated BFV changes using short FUS-off windows immediately before and after sonication. The session-normalized analysis used the 20-s pre-FUS and post-FUS sampling windows specified in section S8, whereas expanded 30-s FUS-off windows were used for the control, vital-sign, and RI summaries where indicated. These windows were selected to assess BFV changes while excluding FUS-on intervals with spectral interference. We normalized BFV based on each trial’s minimum and maximum to compare effects across three (*N* = 3) pigs. The pre- and post-FUS velocity distributions showed session-level increases in BFV following FUS across the collective 30 repeated sessions (*n* = 30) (Fig. 5B). These session-level comparisons are interpreted as exploratory repeated within-animal feasibility observations, not as independent animal-level therapeutic efficacy.

As a control comparison, FUS trials across the three pigs were compared with negative-control periods of similar duration in which FUS was not applied. Session-level ΔBFV was greater during FUS trials than during control periods (*P* < 0.001) (Fig. 5C), supporting repeatability under the tested conditions while remaining subject to the repeated-observation structure of the experiment.

Heart rate and MAP remained stable throughout the analyzed FUS sessions, indicating that the observed BFV changes were not accompanied by large systemic hemodynamic fluctuations under the tested conditions (section S8 and fig. S11). These results support feasibility of localized FUS-associated BFV modulation, while future studies are required to determine mechanisms, dose-response relationships, tissue perfusion effects, and injured-cord efficacy.

### MUSIC implantation biocompatibility and FUS safety

To evaluate the preliminary histological tolerability of MUSIC implantation and FUS exposure, we collected spinal cord samples after euthanasia during the acute postoperative window relevant to SCI monitoring and FUS interventions, up to 2 weeks after implantation. Spinal cord morphology was assessed using hematoxylin and eosin (H&E) and Masson’s trichrome staining (MST), and immunostaining was performed for Iba1 and glial fibrillary acidic protein (GFAP). At day 0, representative uninjured tissues exposed to MUSIC implantation and FUS did not show an obvious gross histopathologic abnormality relative to naïve controls in the sections examined, whereas SCI tissues showed hemorrhage on H&E, increased Iba1^+^ microglial and/or macrophage aggregation, and reduced GFAP^+^ glial signal (Fig. 6A). These observations support preliminary tolerability of the tested FUS exposures but do not establish definitive tissue safety.

**Fig. 6.**
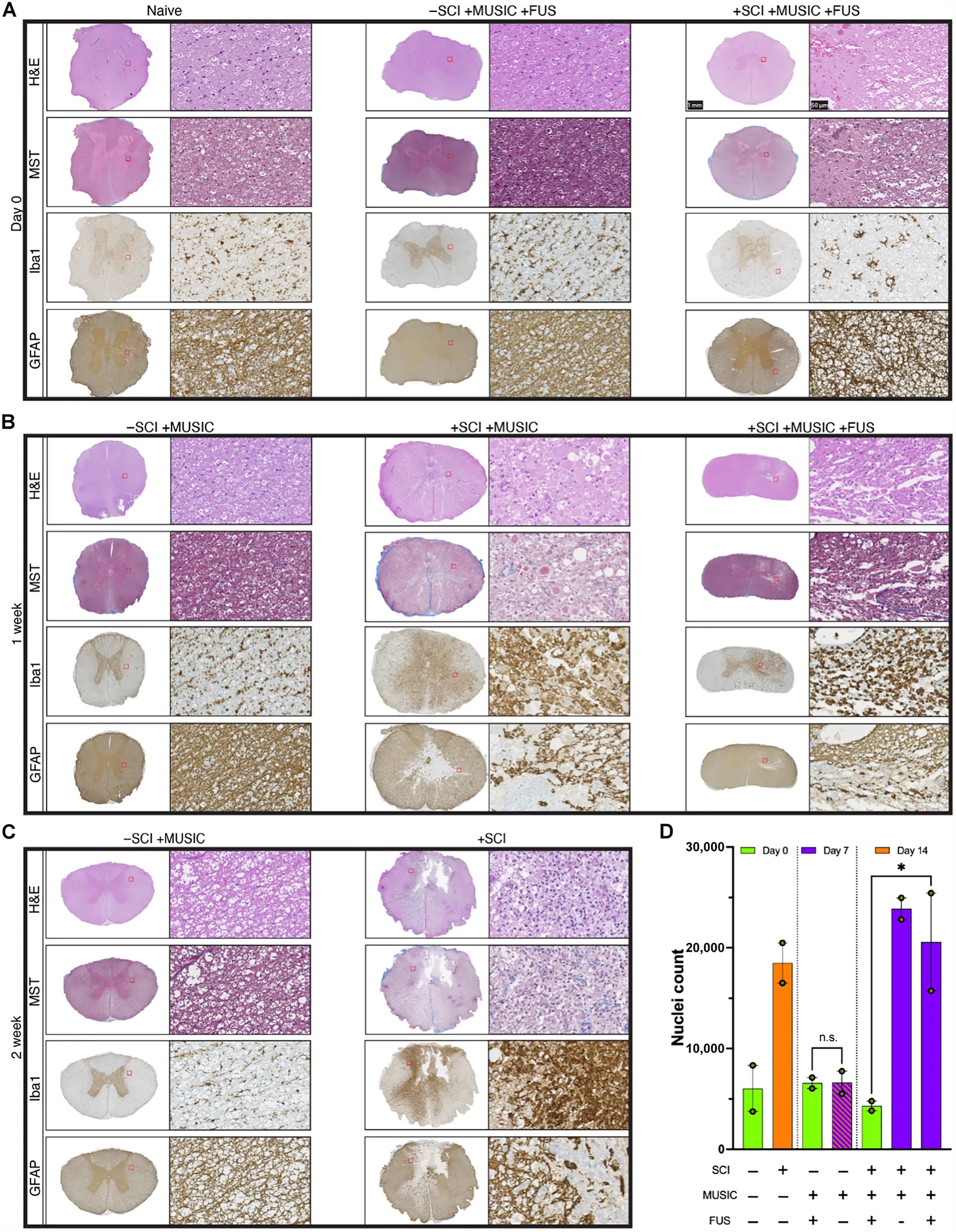
Preliminary histological tolerability of FUS exposure and MUSIC implantation. Representative histology (H&E and MST) and immunohistochemistry (IHC; Iba1 and GFAP) images of porcine spinal cord tissues retrieved from pigs with or without SCI, MUSIC implantation, and FUS exposure. Scale bar, 1 mm (left). Zoomed red-box regions are shown on the right. Scale bar, 50 μm. Because each condition included *N* = 2 pigs per group, these data are presented as a preliminary tolerability assessment and are not powered to exclude subtle histopathologic effects. (**A**) Compared with naïve tissue, the representative +MUSIC +FUS section without SCI did not show a gross histologic abnormality at day 0, whereas SCI tissue showed microhemorrhage on H&E and Iba1^+^ microglial and/or macrophage changes. (**B**) At 1 week, representative sections from MUSIC-only animals preserved gross spinal cord architecture, whereas SCI sections showed axonal spheroids, increased MST-positive scarring, increased Iba1^+^ immune cell presence, and reduced GFAP signal within the lesion region. Within this limited cohort, the addition of MUSIC or FUS was not associated with an obvious additional gross morphologic change beyond the SCI-associated pattern. (**C**) At 2 weeks, representative MUSIC-only sections again preserved gross spinal cord architecture, whereas SCI sections showed progressive tissue disruption, MST-positive scar deposition, increased Iba1^+^ microglial and/or macrophage presence, histological voids, and reduced GFAP signal. (**D**) Exploratory ImageJ-based quantification of nuclei from the H&E panels as a descriptive measure of nuclear density and cellularity. Because the independent biological sample size was *N* = 2 pigs per group, statistical comparisons are presented as exploratory context only, and nonsignificant comparisons are not interpreted as evidence of no effect. *P < 0.05; n.s., not significant.

At 1 week after implantation, representative MUSIC-only samples preserved gross spinal cord architecture, whereas SCI samples showed marked tissue disruption, including axonal spheroids on H&E, increased MST-positive scar formation, increased Iba1^+^ immune cell presence, and reduced GFAP signal within lesion regions (Fig. 6B). Similar SCI-associated histologic disruption was observed regardless of whether MUSIC implantation or FUS exposure was added, suggesting that SCI was the dominant driver of the observed tissue changes in this limited cohort. At 2 weeks, representative MUSIC-only sections again preserved gross spinal cord architecture, whereas SCI sections showed continued tissue disruption, including increased MST-positive scar deposition, increased Iba1^+^ microglial and/or macrophage presence, histological voids, and reduced GFAP signal within the lesion region (Fig. 6C).

ImageJ-based nuclei counts from H&E sections and percent-area quantification of Iba1 and GFAP staining are presented as exploratory, descriptive analyses (Fig. 6D, section S9, and fig. S13). The MUSIC-only day-7 and day-14 specimens were pooled for descriptive comparison with the other groups, while SCI groups showed higher nuclei density over time, consistent with the observed increase in Iba1^+^ microglial and/or macrophage presence. Together, these data show no gross histologic safety signal in the tested cohort and support preliminary tolerability of MUSIC implantation and FUS exposure during the acute postoperative window. Of note, the independent biological sample size was *N* = 2 pigs per group, so nonsignificant comparisons are not interpreted as evidence of no effect. Larger, powered studies are required to evaluate subtle tissue effects, delayed inflammatory responses, angiogenesis, and definitive safety before human translation.

## DISCUSSION

We present MUSIC, a temporary epidural acoustic interface for postoperative spinal cord monitoring and future localized ultrasound intervention. In porcine models, MUSIC provided on-demand B-mode imaging of spinal cord morphology, hematoma tracking, power Doppler imaging, and spectral Doppler-derived BFV monitoring after wound closure. MUSIC does not directly geometrically resolve the sulcal artery lumen or vessel wall: The measured lateral FWHM resolution of the imaging arrays (∼342 to 438 μm) is larger than expected sulcal artery diameters, which vary by species, branch order, fixation state, anatomy, and measurement method and are generally on the order of ∼0.1 to 0.4 mm. Thus, the ∼140-μm value sometimes cited for this vessel should be viewed as an approximate point within this variable range, not as a fixed universal caliber. Instead, MUSIC detects and longitudinally tracks a localized Doppler flow signature within a spectral Doppler sample volume attributable to the sulcal artery region. The attribution of this signal is supported by localization in the expected ventral midline region, reproducible arterial pulsatility synchronized with cardiac cycles, consistent spectral Doppler morphology, and disappearance of the signal during euthanasia. Direct lumen-level microvascular resolution and volumetric perfusion quantification will require future higher-frequency arrays, contrast-enhanced imaging, ultrasound localization microscopy, or histologic coregistration.

The FUS experiments establish proof-of-concept hemodynamic modulation feasibility rather than therapeutic efficacy. In healthy pigs, MUSIC-delivered FUS was associated with session-level changes in Doppler-derived BFV, while heart rate and MAP remained stable during the analyzed windows. These observations do not establish tissue perfusion improvement, vasodilation, injured-cord benefit, lesion containment, tissue preservation, or neurologic outcome improvement. Volumetric blood flow depends on vessel diameter and cross-sectional area, which were not measured, and RI is retained only as descriptive hemodynamic context. Future studies should combine MUSIC with contrast-enhanced ultrasound, ultrafast perfusion imaging, tissue oxygenation measurements, vessel-diameter imaging, and histologic coregistration to establish whether BFV changes correspond to tissue-level perfusion or biologically meaningful protection after SCI.

Delivering FUS to a specific spinal cord region often requires MRI guidance or an external optical tracking system. The complex geometry of spinal vertebrae can attenuate and complicate wave propagation, making cost-effective ultrasound-guided methods challenging to implement. By placing MUSIC after laminectomy, these obstacles are reduced. The present study demonstrates a sensing-informed sequential workflow in which baseline Doppler-derived hemodynamic conditions can be measured, FUS parameters can be selected, FUS can be delivered, and the response can be reassessed. No autonomous controller was implemented. Thus, MUSIC should be viewed as a future closed-loop–capable platform rather than a fully autonomous closed-loop system in its current form (section S10 and fig. S14).

We further demonstrated preliminary MUSIC-based FUS/BFV examples in which the same platform was used for Doppler monitoring and FUS delivery. Both increases and decreases in the localized Doppler signal attributable to the sulcal artery region were observed in preliminary sensing-informed experiments (section S10 and fig. S14, B and C). These examples support the feasibility of integrating monitoring and actuation in a single epidural interface, but they remain exploratory and require prospective dose-response validation, artifact handling, and autonomous-control development before clinical translation.

The mechanisms underlying the observed BFV modulation remain an active area of investigation. Potential contributors include mild thermal effects, acoustic radiation force, endothelial or shear-mediated vascular responses, neuromodulatory or autonomic pathways, and other mechanical interactions between ultrasound and spinal cord microvasculature. The present study was designed to establish feasibility of BFV modulation rather than isolate a dominant mechanism. Future studies can incorporate local thermometry, vessel-diameter measurements, contrast-enhanced and ultrafast perfusion imaging, tissue oxygenation assessments, autonomic blockade experiments, and prospective dose-response designs to characterize the biological pathways responsible for the observed BFV changes.

In addition to hemodynamic modulation, the acoustic outputs of MUSIC overlap with preclinically established exposure ranges for emerging interventional strategies such as neuromodulation, microbubble transport, and blood–spinal cord barrier opening (*55*–*57*). These applications remain future research directions. The present work shows that MUSIC can generate and monitor acoustic and Doppler signals in relevant ranges, providing a platform for mechanistic studies of spinal cord physiology and localized ultrasound bioeffects.

The histology and immunohistochemistry (IHC) data provide preliminary tolerability evidence for the tested MUSIC implantation and FUS exposure conditions. Representative MUSIC-only tissues preserved gross spinal cord architecture at the examined time points, and SCI-associated disruption dominated the observed histologic changes in injured groups. However, because each condition included only *N* = 2 pigs per group, these data are descriptive and not powered to exclude subtle tissue effects, delayed inflammation, angiogenesis, or longer-term safety risks. Larger survival studies with vascular-density markers, local thermometry, longitudinal imaging, and powered histologic end points are needed before definitive tissue safety conclusions or human translation.

Several practical workflow considerations remain for future clinical translation. The current tethered design was developed as a reliable first-generation temporary platform for acute ICU monitoring, with cable tunneling, low-profile routing, flexible strain relief, sterile dressing management, and controlled retrieval incorporated into the design concept. The epidural-device analogy is limited to workflow and form factor; related devices placed on or adjacent to the dura can be associated with cord or root compression, direct trauma, epidural hematoma, infection, fibrosis, and migration under some circumstances. MUSIC therefore requires device-specific validation of placement reproducibility, migration risk, infection management, patient-handling procedures, dressing protocols, longer ICU deployment, and standardized retrieval. Although MUSIC remained implanted for up to 14 to 15 days in porcine studies, its intended clinical use case is primarily the first postoperative week after injury, when the risk of secondary injury is greatest.

The present 45 mm × 10 mm MUSIC configuration is a standardized first-generation implementation, not a one-size-fits-all solution for human SCI. Traumatic SCI lesion dimensions vary substantially with injury level, morphology, and patient anatomy; for example, a 1420-patient traumatic cervical SCI cohort reported a median intramedullary lesion length of 30.8 mm, and multicenter MRI data show that lesion extent is level dependent and prognostically relevant (*58*, *59*). Future iterations may require changes in array spacing, coverage area, modularity, fixation, and deployment strategy for multilevel, noncontiguous, or anatomically constrained injuries. In this regard, MUSIC should be viewed as an adaptable foundational implantable ultrasound platform for postoperative spinal cord monitoring and intervention rather than as a single fixed device design.

Together, these studies support MUSIC as a feasibility-stage neural interface that can be placed during decompression, remain accessible after wound closure, provide on-demand structural and Doppler-derived hemodynamic information, and deliver device-integrated FUS in proof-of-concept experiments. The current evidence supports platform feasibility, preliminary tolerability, and localized BFV modulation in healthy animals. It does not yet establish therapeutic efficacy, autonomous closed-loop control, microvascular lumen resolution, tissue perfusion improvement, or clinical outcome benefit. Future work will focus on injured-model efficacy studies, animal-level statistical validation, biomarker and perfusion coregistration, extended workflow testing, and device iterations optimized for human anatomy and clinical use.

## MATERIALS AND METHODS

### Experimental design

This study was designed to evaluate the feasibility of a multifunctional temporary epidural ultrasound neural interface for postoperative spinal cord monitoring and localized ultrasound actuation. Device design and fabrication, including material selection and schematics, are presented to define the platform architecture. Ultrasound array performance was assessed through simulations and benchtop experiments, followed by human cadaveric placement and porcine in vivo deployment. MUSIC imaging reliability, Doppler-derived hemodynamic monitoring, preliminary histological tolerability, and proof-of-concept FUS-associated BFV modulation were evaluated as feasibility end points. The study was not designed or powered to establish injured-cord therapeutic efficacy, definitive tissue safety, or autonomous closed-loop control.

Human cadaver studies were performed using deceased adult donors obtained through a legally authorized anatomical donation program permitting research use. Because the cadaver studies involved deceased donors and did not constitute human-subjects research under institutional policy, no Institutional Review Board (IRB) approval number or informed-consent protocol number was applicable.

#### Human participant demonstration

A live, noninvasive demonstration of MUSIC as a wearable ultrasound device over the radial artery was conducted under approval of the Johns Hopkins Medicine IRB protocol #00353692. The participant provided written informed consent before participation and before use of the resulting images and video. Data were collected in accordance with the approved protocol, and participant privacy was maintained.

### Device design and fabrication

The MUSIC device has both imaging and FUS arrays for a total of five stacked flex circuits (Fig. 2A). There are individual processes for each type of array. There are three independent 10.5-MHz linear imaging arrays, each of which is 9.20 mm long and 3.47 mm wide, with a 135-μm pitch and 24-μm kerf. Two imaging arrays are placed adjacent to one another (center-to-center distance of 3.4 mm with 1-mm Steralloy 2766 between) to provide two planes of view at the injury site. The third array is located 2 cm distally, providing a self-reference of the healthy cord. Each linear imaging array consists of 64 diced piezoceramic elements, with a 2-mm-thick tungsten oxide backing layer [*Z* = 4.2 megarayleighs (MR)], quarter-wave resin/carbon composite matching layer (*Z* = 5 MR), and an acoustic lens optimized for imaging at depths of 4 to 12 mm.

There are two independent 2.5-MHz FUS arrays spaced 10 mm apart, located on either side of the central imaging arrays (Fig. 2A). The FUS arrays are 9.2 mm long and 3.3 mm wide, with a lambda/2 = 300-μm pitch and 100-μm kerf. Each array consists of eight bar-mode elements designed to target regions on either side of the injury (Fig. 1C). Similar to the imaging arrays, the FUS arrays use a diced piezoceramic. The front of each array was covered with a dielectric material to provide a hermetic seal for patient protection, and a bifurcated flex circuit provided the connection to the eight-element stack. The rear of the FUS arrays was air backed (*Z* = 0 MR) to optimize efficiency. The 2.5-MHz frequency was selected as a practical compromise among spinal target access, lower attenuation than the higher-frequency imaging arrays, available aperture/output efficiency, and beam dimensions compatible with the epidural-to-cord target region. Both imaging and FUS arrays were driven directly from Verasonics Vantage transmit electronics.

After the individual ultrasound stacks and flex circuits were constructed, they were assembled to build the final neural interface (section S3 and fig. S4). A fixture was developed to position each array in the device, with alignment features designed into the flex, and the arrays were bonded into position. Shielding was applied to the arrays, followed by casting the first shot in the mold. This first shot helped position the assembly in the second shot mold and helped define the total coverage of the treatment array. After casting the part, the alignment features were removed, and the assembly was encapsulated in Steralloy 2766. All epoxies used were mixed, degassed, and cured, and then all parts were cleaned and surface treated before bonding.

### Ultrasound imaging acquisition

Data acquisition was performed using the Vantage 256 and Vantage NXT ultrasound systems (Verasonics, WA, USA), which provide an analog front end that can simultaneously transmit and receive up to 256 channels. To interface the fabricated transducers with the Vantage system, we designed and built a connector that was compatible with a universal transducer adapter. To simultaneously acquire B-mode and either color or power Doppler or spectral Doppler, we implemented duplex mode as a single pulse sequence. For spectral Doppler, the rf in-phase and quadrature (IQ) signal could be saved over a preselected region of interest (ROI) and thus served as the basis for later processing. A multithreaded software architecture ensured that saving the data did not introduce delays in the function of the Verasonics software–based ultrasound system that would lead to nonuniformity in the pulse repetition interval. Alternating pulses for B-mode and spectral Doppler ensured that image and spectral information were available at all times. The spectral Doppler pulses were used for both spectrogram and color or power Doppler image generation. The pulse-width–modulated transmit pulse was discretized in pulse widths of one-third the carrier period. The transmitter was active for two-third of each half-cycle, for 6 half-cycles per pulse for spectral Doppler, and 2 half-cycles per pulse for B-mode imaging. A Doppler PRF of 2604 Hz was used, with a 40 pixel (px)–by–20 px IQ region stored, where each pixel’s width and height corresponded to 1 wavelength.

With the saved IQ data available, an arbitrary distribution of spectral Doppler gates was applied to generate Doppler spectrograms. For example, the localized Doppler signal attributable to the sulcal artery region was identified by producing a rectangular spectral Doppler sample volume containing or overlapping the expected vessel region. The pixels in the color Doppler image exhibiting the largest flow power within this ROI were automatically identified and averaged before being input to the spectral Doppler processor.

Our separate triplex acquisition was performed with a plane wave angled at 17° for B-mode. The ensemble length and number of multiangle acquisitions for Doppler were 68 and 11, respectively.

### Benchtop characterization of MUSIC

A network analyzer (model 8753E, Agilent Technologies, MD, USA) was used to characterize the electrical impedance of the individual elements within the diagnostic and therapeutic ultrasound arrays. The electrical impedance curve is used to identify the resonance and antiresonance frequencies of the transducer. The resonance and antiresonance frequencies are the frequencies at which the impedance curve reaches a local minimum and local maximum, respectively.

The pressure maps for the FUS arrays were generated in a 30 cm–by–30 cm–by–30 cm water tank (Sonic Concepts Inc., WA, USA) using a calibrated hydrophone (Model Y-120, Sonic Concepts Inc., WA, USA) mounted to a three-axis stage (Thorlabs, NJ, USA) (*34*). Single-point hydrophone measurements were taken ∼5 to 7 mm away from MUSIC. These readings were used to calculate the MI and the *I*_SPPA_ of the FUS arrays under the assumption that the time-varying pressure is a symmetric sinusoidal waveform. These parameters provide acoustic exposure context for the tested configuration; they do not by themselves determine thermal damage or cavitation risk. The MI is calculated according to Eq. 1 (*60*)

$$\text{MI}=\frac{p_{r}}{\sqrt{f_{0}}}$$ (1)

For MI calculations, *p*_r_ denotes peak rarefactional pressure (in megapascals), and *f*_0_ denotes the center frequency (in megahertz). Acoustic exposure was further characterized by pulse duration, PRF, duty cycle, and insonation duration, as cavitation and thermal bioeffects depend on the complete exposure condition rather than pressure alone.

The *I*_SPPA_ is calculated according to Eq. 2 (*61*)

$$I_{\text{SPPA}}=\frac{P_{i}}{P_{D}}$$ (2)

where *P*_i_ is the pulse intensity integral and *P*_D_ is the pulse duration. The pulse intensity integral is calculated according to Eq. 3 (*61*)

$$P_{i}=\int_{0}^{P_{D}}I\;\mathrm{dt}$$ (3)

where *I* is the pulse intensity, which is calculated according to Eq. 4 (*61*)

$$I=\frac{p^{2}}{\rho c}$$ (4)

where *P* is the time-varying pressure (assumed to be sinusoidal) and ρ and *c* are the density and speed of sound of the medium, respectively. For these water tank scans, the propagation medium was water. The pulse-echo response was measured by placing an aluminum plate under MUSIC. MUSIC was connected to the Vantage NXT system to coordinate the transmission of an ultrasound pulse and storage of the corresponding echo data. A Fourier transform was applied to the received data, and the −6-dB width was used to measure the bandwidth of the transducer. The experimental outputs of MUSIC were compared to simulated values (section S11 and figs. S15 to S24) and benchmarked to theoretical computations (section S12, figs. S25 to S40, and eqs. S2 to S27).

To characterize the B-mode images captured with MUSIC, we used the Multi-Purpose Multi-Tissue Ultrasound Phantom (model 040GSE, Sun Nuclear, FL, USA). With this tissue phantom, we calculated resolution, penetration depth, and image quality. These measurements were collected and compared with a state-of-the-art clinical ultrasound system (Philips EPIQ 7 with L15-7io transducer; Philips Healthcare, MA, USA). Resolution was quantified by measuring the FWHM of image amplitude through 100-μm point targets at a depth of 1 cm. Penetration depth was measured by identifying the deepest 100-μm point target visualized with at least 6-dB contrast. Image quality was assessed by calculating contrast (*C*), gCNR, and SNR of an anechoic cyst using Eqs. 5 to 7 (*62*–*64*)

$$C=20\;{\text{log}}_{10}\left(\frac{\mu_{t}}{\mu_{b}}\right)$$ (5)

$$\text{SNR}=\frac{\mu_{b}}{\sigma_{b}}$$ (6)

$$\text{gCNR}=1-\sum_{k=0}^{N-1}\text{min}\left\{h_{t}\left(x_{k}\right),h_{b}\left(x_{k}\right)\right\}$$ (7)

where μ_t_ and μ_b_ are the mean pixel amplitudes of target and background ROIs, respectively, and σ_b_ is the SD of the background ROI. *h*_t_ and *h*_b_ are histograms of the target and background ROIs, respectively, with *N* bins centered at {*x*_0_, *x*_1_,*…*, *x*_*N–*1_}.

To characterize the Doppler images captured with MUSIC, we used the Doppler String Phantom (model 043A, Sun Nuclear, FL, USA). A continuous flow rate of 10 cm/s was set to determine the accuracy of the measured PDI signal. Pulsatile flow with the preset “common carotid artery” setting was used to assess the spectral Doppler accuracy.

### Porcine protocols and specifications

All terminal and survival studies were conducted using porcine models following procedures approved by the Johns Hopkins Animal Care and Use Committee, protocol number: SW23M249 (section S13). Yorkshire pigs weighing 35 to 40 kg were tested, all of which were acclimated for 7 days before the start of the study. Animals were housed according to standard guidelines, and steps were taken to minimize pain and distress, including the use of anesthesia and humane end points when applicable. After anesthesia induction, we monitored and recorded every 15 to 30 min several parameters: heart rate, blood pressure, blood oxygen and carbon dioxide, respiratory rate, and rectal temperature. Hypothermia was prevented using warming blankets. Additional details on the surgical procedure are provided in section S13.

Pigs received a laminectomy at T4 to T6 (fourth through sixth thoracic vertebrae) to expose the spinal cord. Baseline ultrasound images were collected using MUSIC and a Canon Aplio i800 with an i22LH8 transducer (the latter serving as the clinical state-of-the-art comparison). These baseline images included B-mode, PDI, and spectral Doppler data. The range of FUS acoustic parameters is summarized in Table 1. The MUSIC device was secured over the spinal cord via a custom rail system connected to spinal hardware (figs. S5 and S6). The pigs were divided into two experimental groups: intraoperative and postoperative pigs as seen in Table 2.

**Table 1.** Overview of six experimental acoustic parameters for the pigs in group 1.

Pig #	*I*_SPPA_ (W/cm^2^)	Input voltage (V)	PD (ms)	PRF (kHz)	Duty cycle (%)	Insonation period (s)
1	1.22	60	0.07	1–5	7–35	120–300
2	0.041–1.49	20–80	0.07–0.40	1–5	7–35	120–600
3	5.41	20	0.07–0.35	1–5	7–40	120–600

**Table 2.** Approximate sample sizes for intraoperative and postoperative usage of MUSIC modalities. FUS + hemodynamic monitoring indicates sessions in which FUS-induced hemodynamic changes were tracked with MUSIC spectral Doppler or power Doppler capability.

Ultrasound modality	Intraoperative pigs	Postoperative pigs
Brightness mode imaging	25	8
Power Doppler imaging	8	5
Spectral Doppler imaging	5	3
Therapeutic ultrasound (FUS)	7	2
FUS + hemodynamic monitoring	3	2

Group 1 involved assessing the diagnostic capabilities and FUS feasibility intraoperatively in healthy and injured porcine models (Fig. 4). The SCI was induced by either a 20-g weight dropped from a height of 15 cm or a custom spinal cord impactor over the center of T5 (*65*). After injury, repeated B-mode, PDI, and spectral Doppler images were acquired to assess the effect of injury on morphology and BFV. MUSIC-delivered FUS was evaluated in healthy pigs in concert with the Canon Aplio i800 ultrasound system’s i22LH8 transducer. This commercial transducer was used to identify a localized Doppler signal attributable to the sulcal artery region and record the corresponding Doppler spectrogram (Fig. 5A). Spectral Doppler was recorded using the commercial probe, while FUS was performed with MUSIC. The commercial probe and MUSIC were positioned such that their corresponding fields of view overlapped. We confirmed overlap through visualization of interference patterns in the spectrogram while FUS was administered (fig. S10), indicating that MUSIC’s FUS arrays insonated the Doppler signal region. In addition, healthy pigs were used to demonstrate a preliminary sensing-informed workflow (fig. S14, B and C), in which both spectral Doppler and FUS were performed with the MUSIC device. We note that there was a delay between FUS application and spectral Doppler recording, as we switched between ultrasound transmission scripts during preliminary sensing-informed testing.

Group 2 evaluated the postoperative capabilities of MUSIC. Select pigs outside the therapeutic-ultrasound cohort in group 1 were survived for up to 15 days. At the conclusion of the surgical procedure, the incisions were closed, and the pigs were housed in the recovery room. B-mode, PDI, and spectral Doppler images were acquired while the pig was awake and moving (movie S4) and under sedation. MUSIC-delivered FUS was deployed in two pigs as a postoperative feasibility and tolerability assessment. Anatomical geometries evolved during the postoperative healing period, as expected after laminectomy and implantation; these observations are reported as feasibility data rather than definitive evidence of chronic safety. At the conclusion of the studies, pigs were euthanized, and tissue samples from the SCI site underwent histopathological analysis to evaluate host response to the device.

### Histological H&E and MST

Retrieved tissues were fixed overnight using 10% neutral-buffered formalin at 4°C. Postfixation and just before sample processing for paraffin embedding, tissue samples were washed using 70% alcohol. Tissues in formalin-fixed, paraffin-embedded blocks were then sectioned at 5-μm thickness using a microtome, placed on charged superfrost slides, and stained according to standard histological protocols (H&E: for assessing cellular infiltration or MST: to assess collagen deposition and scarring).

### IHC staining

The unstained paraffin slides were stained according to standard IHC protocols. Iba1 (019-19741, Fujifilm) and GFAP (14-9892-82, Thermo Fisher Scientific) were used at concentrations of 2.5 μg/ml (1:1000) and 5 μg/ml (1:500), respectively.

### Deep learning model training and validation for automatic SCI monitoring

For injury localization, images with a hematoma present were labeled with a bounding box surrounding the injury. The ground truth image masks were created in Computer Vision Annotation Tool (CVAT) (CVAT.ai Corporation, CA, USA). The object detection dataset consisted of 400 images of the injured porcine spine and 400 images of uninjured porcine spine. The equal number of true negative images ensured that the model does not overfit and falsely detect hematoma. We trained YOLOv8 (*41*), a state-of-the-art object detection model, which uses an anchor-free architecture for object detection across various sizes and shapes. The dataset was split so that 80% of the images were randomly selected for the training set, 10% for the validation set, and 10% for the test set. To improve the models’ robustness and avoid overfitting, we performed data augmentations during training. The YOLOv8 model was trained on a Windows 11 computer (8-gigabyte RAM) with 24-gigabyte NVIDIA GeForce RTX 3090 graphics unit and a 14th Gen Intel Core i5-14600 processor (14 cores, 20 threads, 2.7- to 5.2-GHz turbo frequency).

For model evaluation, we observed four parameters: mAP@50, @50-95, precision, and recall. mAP is a commonly used metric ranging from 0 to 1, which measures the accuracy of object detectors, as it provides a comprehensive assessment of their performance, considering both precision (how accurately objects are detected) and recall (how many relevant objects are detected) at multiple levels of confidence (i.e., 50% and 50 to 95% at intervals of 5%). The levels of confidence, known as the intersection over union (IoU) thresholds, consider a detection to be accurate if the IoU of the ground truth bounding box and the predicted bounding box is greater than the set threshold (e.g., 0.5 for mAP@50).

In addition, the SAM 2 (Meta FAIR) demo was used to illustrate how MUSIC’s B-mode outputs could be incorporated into existing models without training to perform spinal cord segmentation (*42*). We used three user inputs to initiate segmentation: two inputs selecting the cord (positive input) and one selecting the background region (negative input). With these limited user inputs, the model can effectively generalize across the ultrasound images while remaining robust to noise and motion artifacts.

### Hemodynamics analysis based on spectral Doppler

The time-series data were first processed using a band-pass filter before extracting the envelope of the spectrogram. Subsequently, the maximums [i.e., peak systolic velocity (PSV)], minimums [i.e., end diastolic velocity (EDV)], and mean velocity were analyzed for each FUS session. While FUS was actively applied, interference patterns in the spectrogram precluded analysis during the FUS-on segment. Thus, we assessed the impact of FUS on BFV using FUS-off windows immediately before and after each FUS session; the session-normalized BFV comparison used 20-s pre/post sampling windows, and expanded 30-s windows were used for the corresponding control, vital-sign, and RI summaries where indicated. The resistivity index is a Doppler-derived descriptive metric related to downstream resistance or impedance within the sampled vessel region. RI was calculated using the extracted spectral envelope and is not interpreted as independent proof of vasodilation, volumetric flow change, or improved tissue perfusion. The formula for the resistivity index is as follows

$$\text{RI }=\;\frac{\text{PSV}-\text{EDV}}{\text{PSV}}$$ (8)

#### Artificial intelligence–assisted code generation

OpenAI ChatGPT was used for limited MATLAB code generation supporting exploratory Doppler resistivity-index analysis, spectral-envelope processing for supplementary sensing-informed FUS analyses, and acoustic-exposure calculations. No study data were uploaded to ChatGPT; generated code was executed locally. All AI-assisted code used in reported analyses was reviewed by the authors, and outputs were independently verified against source data and manuscript equations. No identifiable or confidential research data were entered into the platform.

## Supplementary Materials

### The PDF file includes:

Sections S1 to S19 Figs. S1 to S43 Tables S1 and S2 Eqs. S1 to S28 Legend for data S1 Legends for movies S1 to S11 References
