# Supplementary material for: Ultrasound neural interface enabling acute spinal cord monitoring and blood flow modulation
Source: Sci Adv. 2026 Nov 4;12(45):eaeg2342. doi: 10.1126/sciadv.aeg2342 (PMC13733905; doi:10.1126/sciadv.aeg2342)
Supplement: Supplementary file 1 [file sciadv.aeg2342_sm.pdf]

Science Advances
AAAS

Supplementary Materials for

Ultrasound neural interface enabling acute spinal cord monitoring and blood flow modulation

Max J. Kerensky *et al.*

Corresponding author: Amir Manbachi, amir.manbachi@jhu.edu

Sci. Adv. **12**, eaeg2342 (2026)
DOI: 10.1126/sciadv.aeg2342

The PDF file includes:

Sections S1 to S19
Figs. S1 to S43
Tables S1 and S2
Eqs. S1 to S28
Legend for data S1
Legends for movies S1 to S11
References

Other Supplementary Material for this manuscript includes the following:

Data S1
Movies S1 to S11

Supplementary Section 1
MUSIC driver electronics

In order to minimize the size of the implantable device itself, an external belt was designed to house the interface electronics. The implantable portion of MUSIC is inserted into the thoracic or lumbar spine, while the external belt is designed to wrap around the patient's pelvis. The belt contains a 3-D printed enclosure that houses the interconnections between the device and the driver electronics (Figure S1). This architecture allows MUSIC to fully disconnect from the external hardware at the end of the flex circuit, reducing the amount of equipment attached to the patient when the system is not actively being used.

To interface MUSIC with the Verasonics Vantage system, external electronics were used rather than incorporating additional circuitry into the implant itself. The final configuration used a simple 1 μH shunt inductor, which provided the best usable bandwidth for the device despite a non-ideal impedance match. An alternative LC configuration consisting of a 0.68 μH shunt inductor and a 100 pF series capacitor was also evaluated. Although this configuration improved impedance matching, it reduced bandwidth and was therefore not selected for the final device. Because the Verasonics Vantage system provided sufficient voltage and power headroom, the final design prioritized usable bandwidth over ideal power transfer.

The 3-D printed enclosure is shielded to minimize EMI/RF interference during signal acquisition. This belt serves as a proof-of-concept external interface for the research platform and provides a pathway toward future miniaturization and integration of these electronics directly into the MUSIC device architecture, reducing overall system bulk.

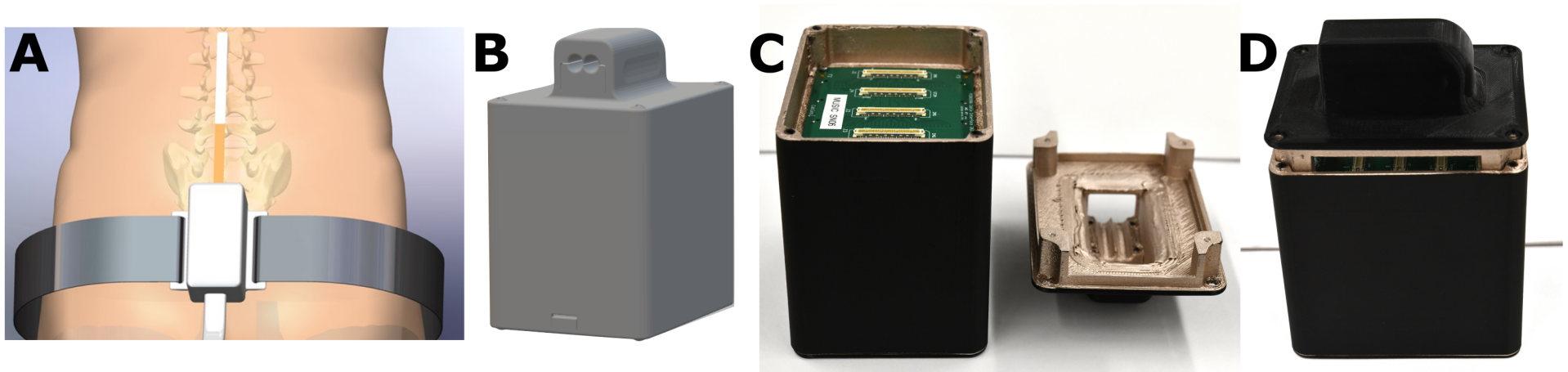

Figure S1: **External Interface Electronics for MUSIC . (A)** Schematic of an external belt housing the interface electronics. **(B)** Computer-aided design (CAD) model and **(C)** fabricated bio-belt housing the circuitry used to connect the MUSIC flex cables to the Verasonics Vantage system. **(D)** The enclosure protects the electronics from biological tissue and external handling during operation.

Supplementary Section 2
Image quality benchmarking

We compared the benchtop image quality metrics for MUSIC with those obtained using a Philips L15-7io transducer connected to a Philips EPIQ 7 ultrasound system (Philips Healthcare, MA, USA). The Philips L15-7io transducer is a linear, 128-element, hockeystick probe with a 7-15 MHz bandwidth. The aperture length of the clinical probe was 23 mm. We selected this probe as the clinical ground truth comparison due to its bandwidth and center frequency being similar to those of MUSIC.

Similar to MUSIC, the resolution of Philips system was quantified as the full-width half maximum (FWHM) of 100 μm diameter point targets located at a depth of 1 cm using B-mode images acquired of the CIRS Multi-Purpose Multi-Tissue Ultrasound Phantom (Model 040GSE; Sun Nuclear, FL, USA). The measured axial and lateral resolution with the Philips L15-7io was 554±47 μm and 429±9 μm, respectively. The axial and lateral FWHM when acquired with MUSIC operating at 10.5 MHz were 326±68 μm and 438±35 μm, respectively. There was no significant difference between the resolution measured with MUSIC and Philips (axial FWHM: $p = 0.0695$; lateral FWHM: $p = 0.5469$; Figure S2A).

When operating MUSIC at 16 MHz, there was a significant improvement in lateral resolution (compared to operating it at 10.5 MHz) (342±29 μm; $p = 0.0170$). While there was a decrease in the axial FWHM at 16 MHz compared to 10.5 MHz (286±29 μm), the reduction was not significant ($p = 0.3188$; Figure S2B).

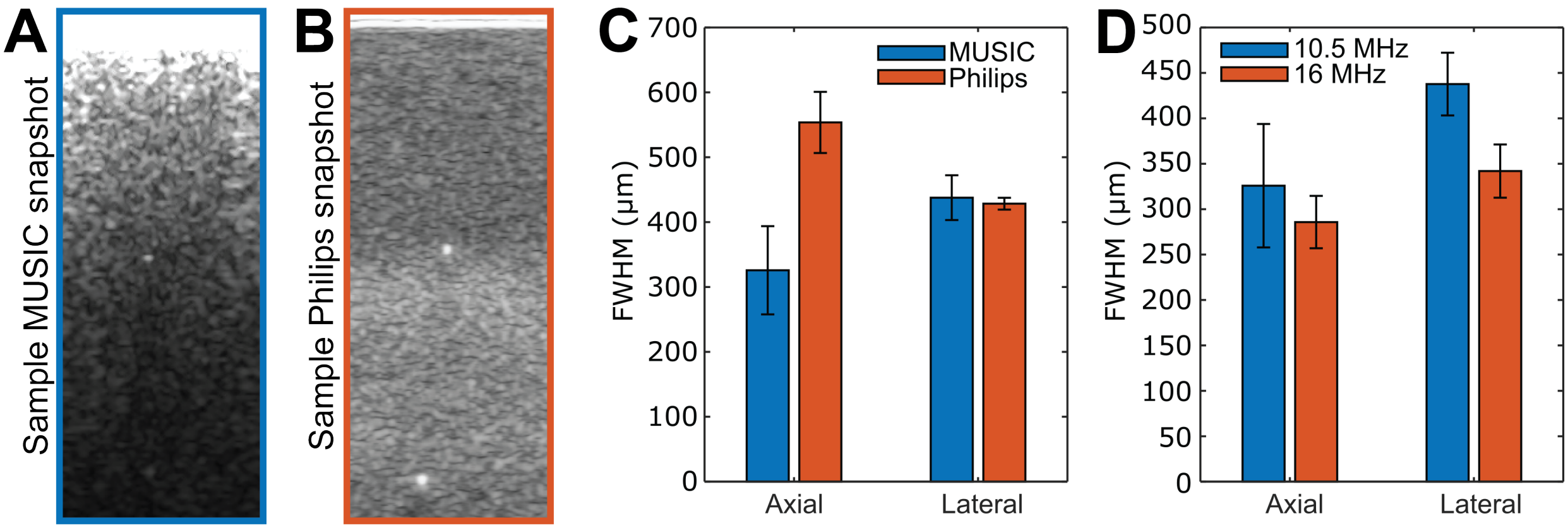

Figure S2: **Comparison of image quality of MUSIC and the Philips L15-7io clinical ultrasound system.** **(A)** A B-Mode benchmarking acquisition is shown for MUSIC. **(B)** A Philips benchmarking acquisition is shown for context. **(C)** Full width half maximum of the point spread function through 100 μm diameter point targets in a B-mode tissue phantom. The axial and lateral measured values compare MUSIC when operating at a 10.5 MHz center frequency and the Philips L15-7io transducer, and **(D)** MUSIC operating at 10.5 MHz and 16 MHz center frequencies. Mean $\pm$ standard deviation are shown.

Supplementary Section 3
MUSIC device housing and wiring assembly

The flex circuit consists of two copper layers with polyimide dielectric and a stiffener on the connector end. Each element on a given imaging array was connected to a single pin on the Hirose 80-pin connector, while each element on a given therapeutic array was connected to two pins for higher current capacity. The total length of each imaging and therapeutic flex is 50 cm. The length from the exit plane of the matching network box to the beginning of biocompatible formed material (i.e., Steralloy 2766) is 30 cm.

To improve shielding and mitigate for electromagnetic noise, metallized cloth duct tape (1502) was added. The increased distance between the tape and the signal-carrying portion of the cable reduces parasitic capacitance, thereby preserving signal integrity.

The form factor of the finished device was comparable to Jackson-Pratt drain commonly inserted along the spinal cord to drain surgical fluid (Figure S3). The layered assembly of MUSIC can be seen in (Figure S4)

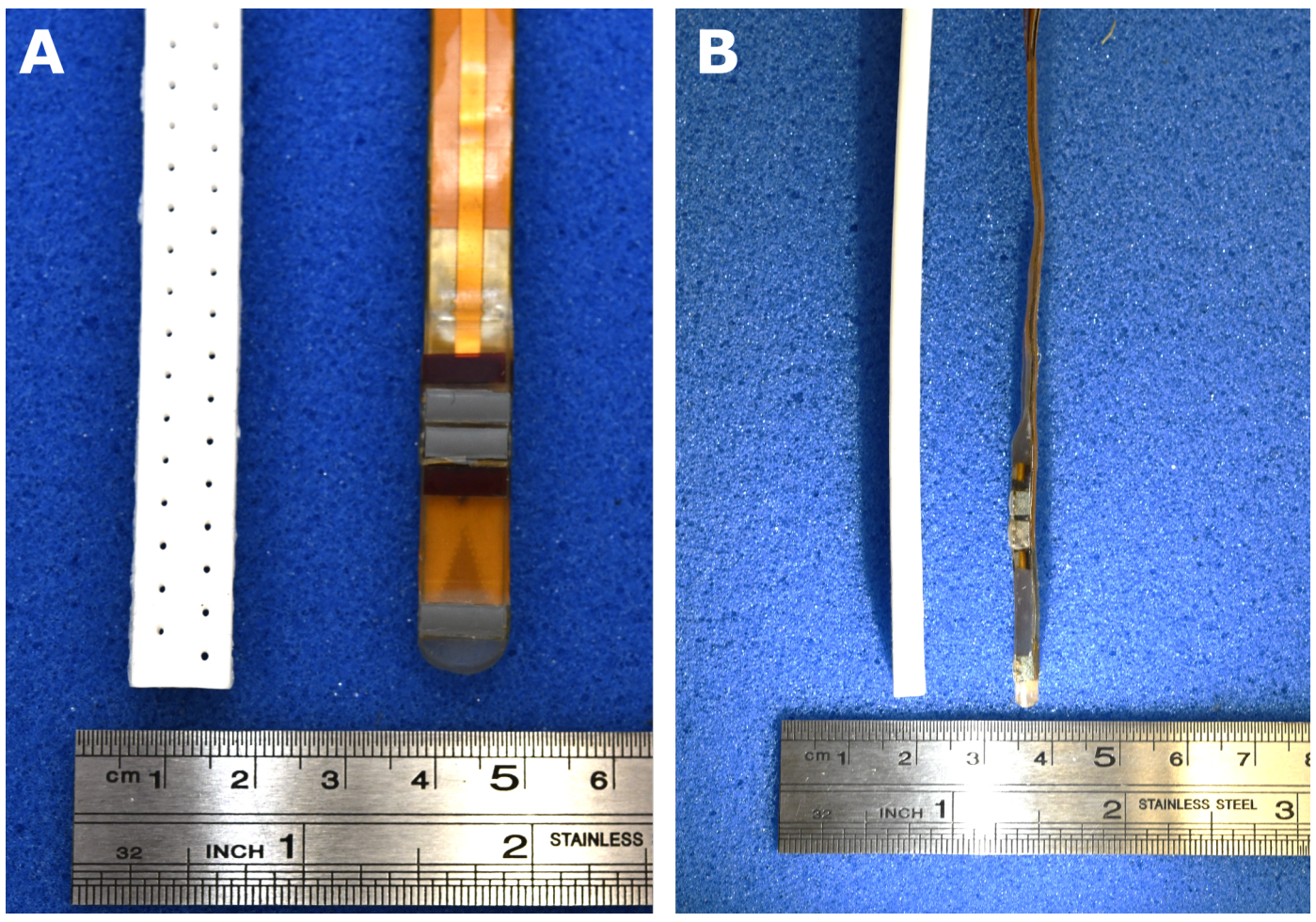

Figure S3: **MUSIC is comparable in size to a Jackson-Pratt drain. (A)** Top and **(B)** side views of Jackson-Pratt (JP) surgical drain compared to MUSIC.

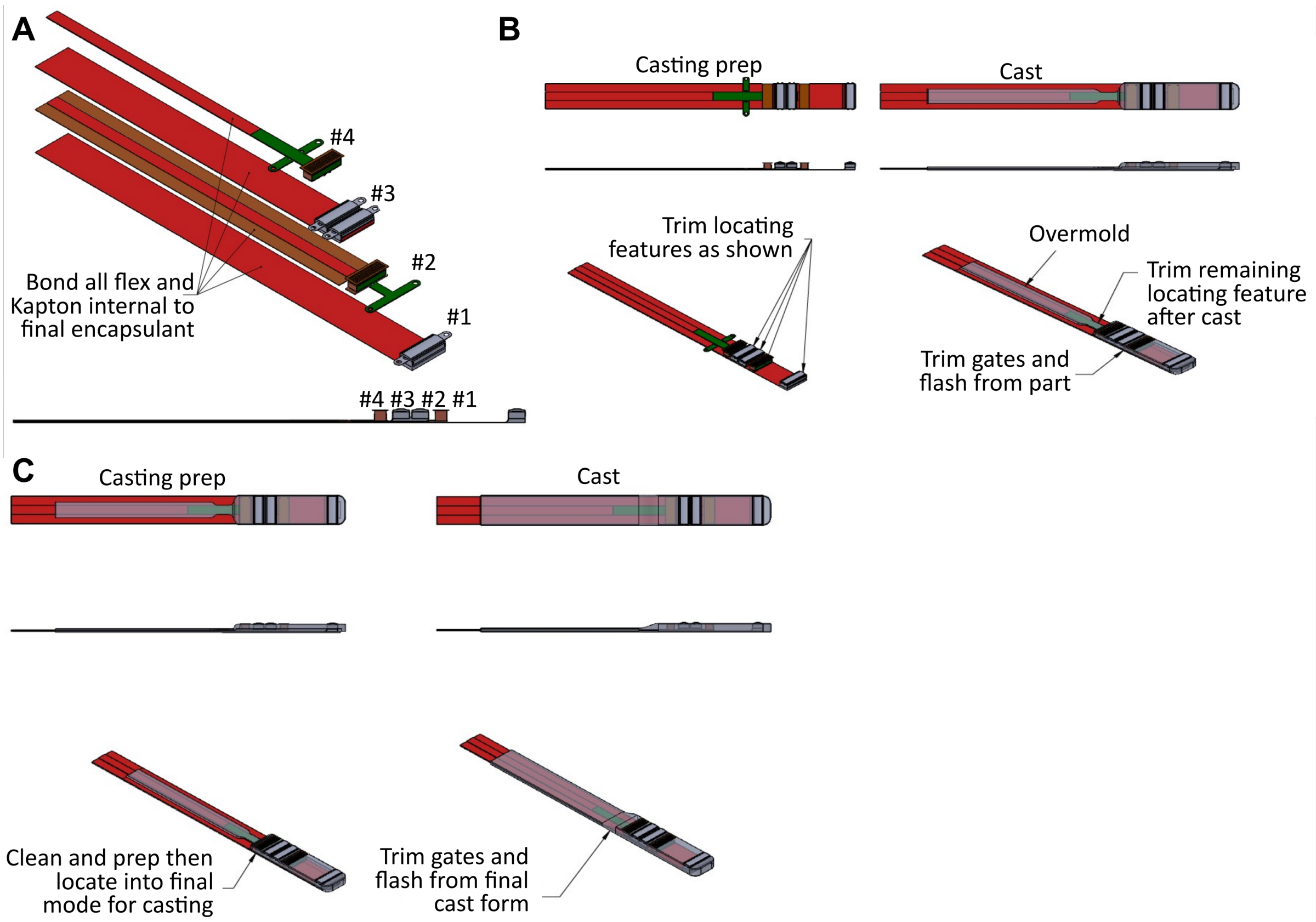

Figure S4: **Assembly of MUSIC** in **(A)** Stage 1, **(B)** Stage 2, and **(C)** Stage 3. The subtasks include bonding the back spacer to the transducer and forming the flex. We bonded the back spacer to the transducer and formed flex, trimmed the flex alignment to suit the position, bonded the transducer and Kapton flex spacers to the stack and clamped. Then, we assembled imaging array transducers together with a backing spacer and bonded the assembly to the stack in the fixture and clamped. Finally, we trimmed the flex as shown and bonded and clamped to the transducer stack assembly.

Supplementary Section 4
Rail system for MUSIC

To secure the device, a custom rail system was designed to fix the MUSIC device over the spinal cord (Figure S5). During the standard decompressive laminectomy, spinal hardware (e.g., spinal rods and pedicle screws) are commonly placed to stabilize the spine. The designed rail system allows for lateral and vertical adjustments based on anatomical variation of the pig (Figure S6). The rail system attaches MUSIC to standard spinal hardware (i.e., rods and pedicle screws) to provide a smooth transition into the clinical workflow.

The selection of specific pedicle screw and rod constructs is dictated by patient anatomy and a review of the patient's preoperative imaging scans. The MUSIC device is compatible with any commonly used, FDA-cleared pedicle screw and rod system. The current rail system is compatible with spinal rods of 3.5 mm and 5 mm in diameter; however, the bracket can be adjusted to any commonly used spinal rod sizes.

The pipeline for MUSIC device placement is given in **Fig. 3A**. First, in the operating room MUSIC is loosely inserted into the rail system attachment pieces. Then, pedicle screws are placed in the vertebrae surrounding the injury location. Next, the full rail system with MUSIC is implanted into the surgical site. Any necessary lateral, elevational, vertical, or rotational adjustments can be made before securing the set screws to lock MUSIC in place. These location adjustments are made while visualizing the B-mode and Doppler images in real-time to ensure sufficient postoperative visibility.

To evaluate the feasibility of the rail system in human anatomy, the system was implanted in an adult human cadaver using a non-functional replica of the MUSIC device (Figure S7). This assessment supported compatibility with the anatomical variation encountered in the tested specimen.

In 13-20% of cases, spinal cord implants experience migration of spinal cord stimulator lead electrodes to areas where they are not intended to be, and this issue often cannot be addressed by reprogramming (*66*, *67*). Given the geometric similarities between MUSIC and spinal cord stimulators, the rail system could also be used to stabilize implanted electrical stimulators.

The rail system was designed to provide stable temporary fixation while preserving controlled access to the device. This design rationale is important for a temporary tethered implant, where maintaining access can reduce the risk of device migration, loss of retrievability, or need for additional operative exposure. In a future clinical workflow, the external flex cable could be tunneled to a remote lumbar exit site, allowing device access without reopening the primary surgical field. Future rail-system iterations may incorporate customizable release features to further standardize retrieval after the intended monitoring period.

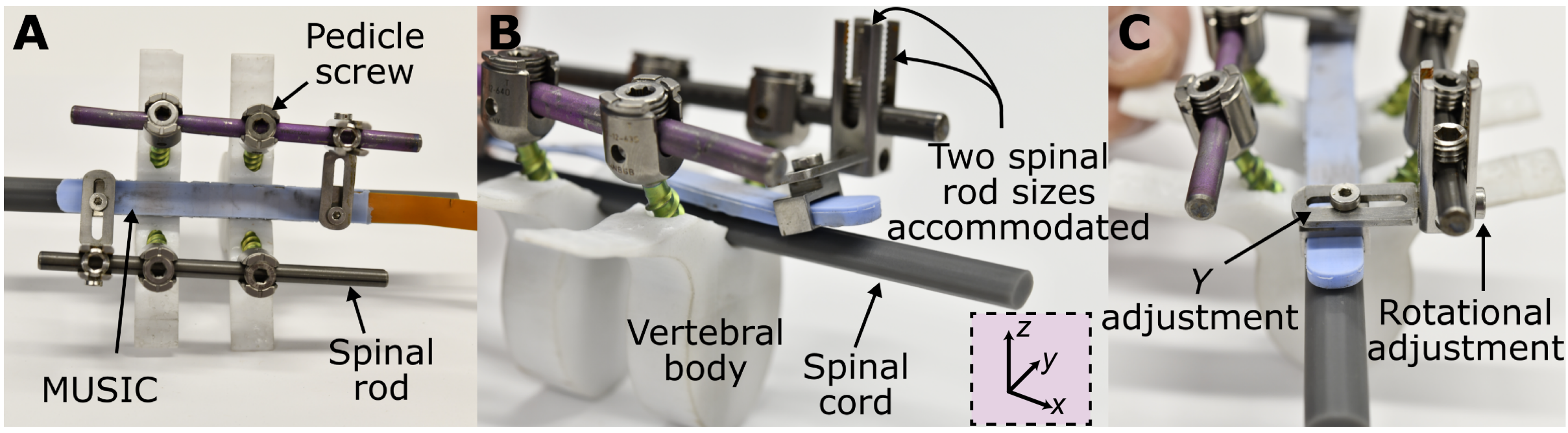

Figure S5: **Customized rail system for the spinal cord.** **(A)** Photograph of the full rail system placed in 3D printed vertebrae connected with commercially used pedicle screws and spinal rods. **(B)** The barrel was designed to accommodate both 3.5 mm and 5 mm diameter spinal rods. **(C)** Set screws allow for translation in the Y direction and rotation about the Y axis to accommodate anatomical variation (**Supplementary Video 5**).

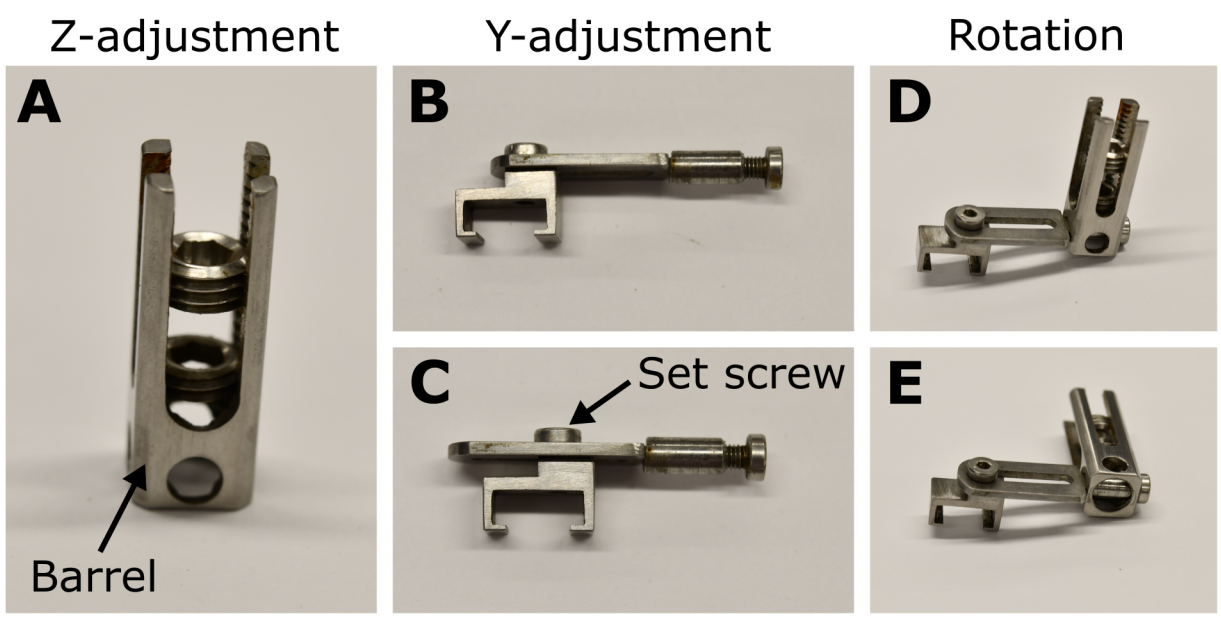

Figure S6: **Individual components of the rail system allowing for adjustments in 3-dimensional space. (A)** The barrel allows for vertical adjustment in the Z-direction. Sliding the spinal rod through the barrel before tightening allows for adjustment in the X-direction (along the length of the spinal cord). **(B-C)** A center screw allows for lateral adjustment in the Y-direction (along the width of the spinal cord; **Supplementary Video 6**). **(D-E)** The barrel can rotate about the Y-axis. These adjustments allow for personalized placement of the rail system in a wide range of anatomical sizes and conditions.

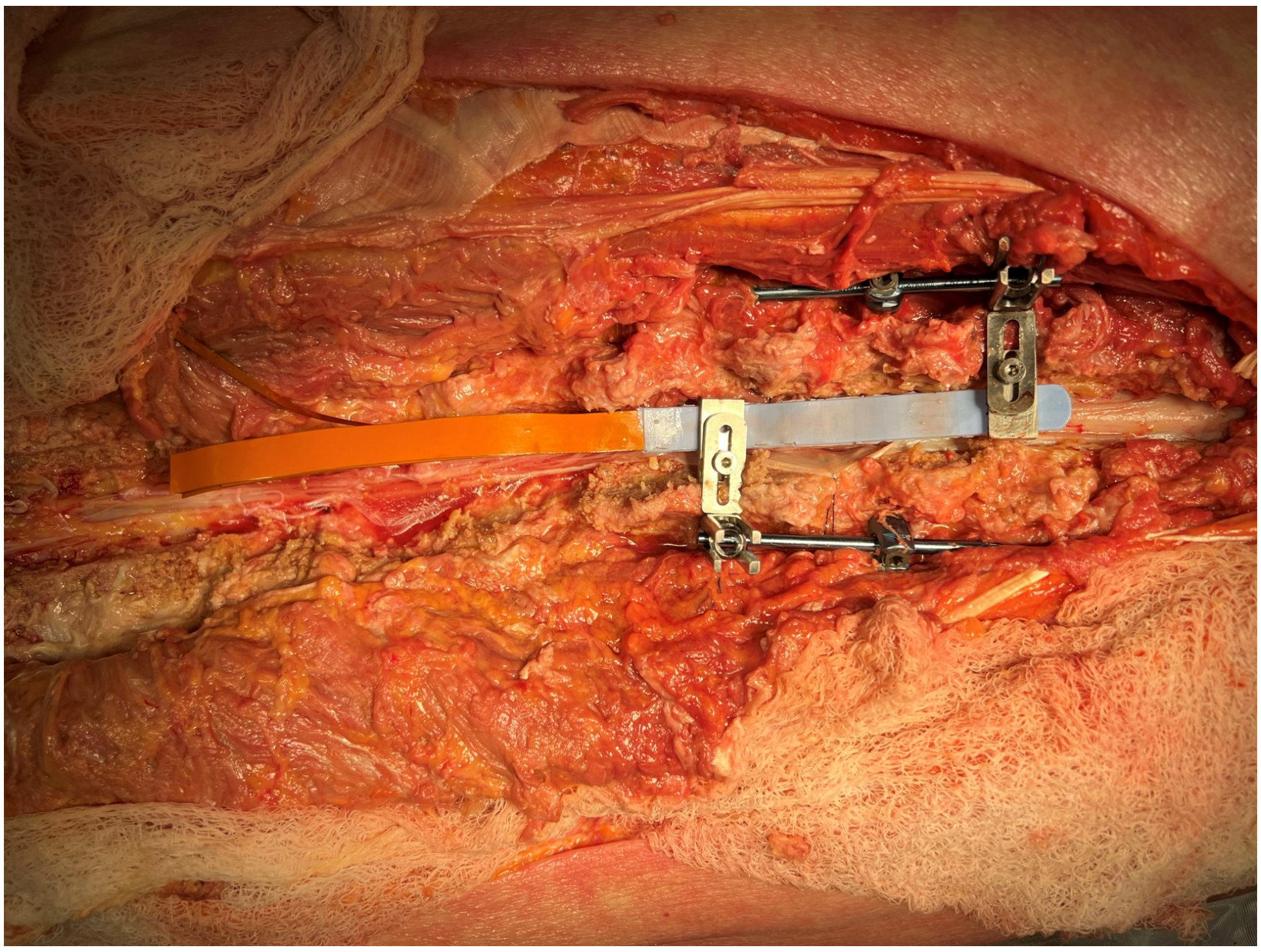

Figure S7: **The rail system was successfully implanted in a human cadaver.**

Supplementary Section 5
Automated hematoma detection

Hematoma localization was performed using a YOLOv8 (*41*) backbone and B-mode images acquired by MUSIC as inputs. Data augmentation included image transformations (i.e., rotating the samples with a degree range of -25 to +25, horizontal flipping with a probability of 0.5, and randomly zooming in or out of the image by 30%) and image degradation (i.e., blurring the image by a factor of 3 with a probability of 20%). We also adjusted the image appearance by randomly manipulating the statistical characteristics of the image intensities such as brightness, saturation, hue, and contrast by a factor of ±0.2.

The model was tuned to obtain the optimal set of hyperparameters using a dataset of ultrasound images obtained from a Canon Aplio i800 ultrasound system (Canon Medical Systems, Tustin, CA) connected to an i22LH8 transducer or an i18LX5 transducer (*68*). The hyperparameter tuning was conducted using Neural Network Intelligence (NNI), which is a toolkit to automate and accelerate the process of finding the optimal set of hyperparameters that minimizes the loss function (*69*). The batch size and number of epochs were selected from a discrete set of options informed by previous studies. Similarly, the learning rate was sampled from a predefined range based on prior research. To ensure a balanced and efficient exploration of the hyperparameter space, we employed a log-uniform sampling strategy, giving equal probability to each order of magnitude. The parameter search space for each of the hyperparameters is as follows: (1) Batch size: 16, 32, 48, 64, (2) Learning rate: [0.0001, 0.1], (3) Epochs 40, 60, 80. After 50 trials, the optimal hyperparameter combination that achieved the lowest error included a batch size of 48, learning rate of 0.000422, and 80 training epochs.

All tests were performed on previously unseen spinal cord images, demonstrating the generalizability of the model. We were able to identify and track the hematoma (when present) in real-time, with the model processing at an average frame rate of 18 fps on a CPU and 115 fps on a GPU. Additional model characteristics are included in Table S1.

To assess the accuracy of our hematoma detection algorithm, we used the mean average precision (mAP). The mAP incorporates both precision and recall (Figure S8A-B), allowing the user to understand both how accurate and how many relevant objects are detected to ensure that our model does not pick up irrelevant anatomy. Our tuned model achieved mAP@50 of 99% when hematomas were present (Figure S8C). Due to the critical nature of spinal cord injuries, we employed a more stringent metric (i.e., mAP@50-95), achieving an accuracy of 60% (Figure S8D).

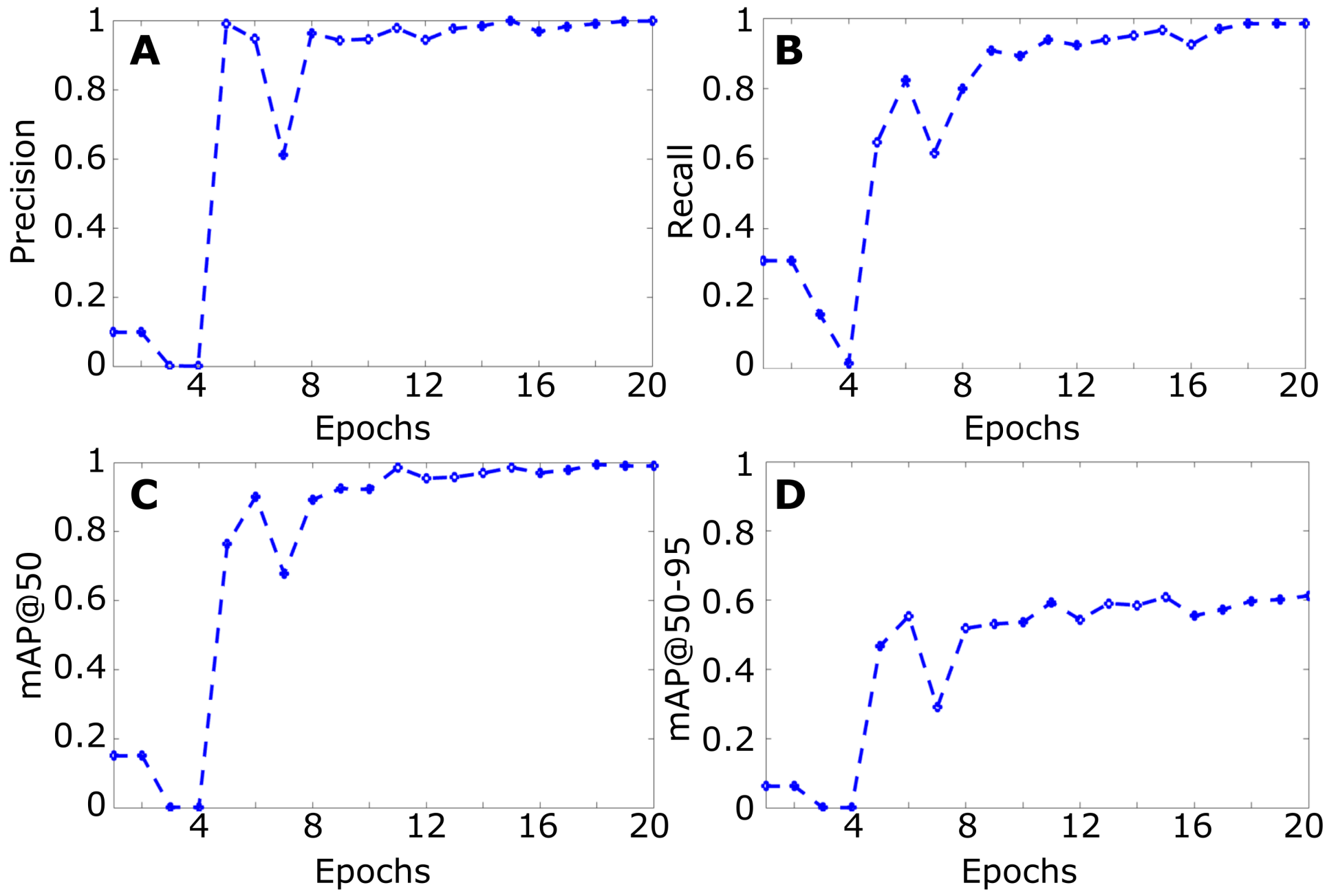

Figure S8: **Accuracy of the hematoma detection algorithm. (A)** Precision, **(B)** recall, **(C)** mean average precision (mAP) @50, and **(D)** mAP@50-95 for hematoma detection within B-mode images captured with MUSIC.

Number of parameters	25,856,899
Batch size	8
Learning rate	0.0437
Training epochs	20
Loss function	Binary cross entropy + distribution focal loss + complete intersection over union

Table S1: **Hematoma localization model characteristics.**

The spinal cord segmentation and tracking was performed using SAM2 (*42*). Two positive points and one negative point were selected on the first frame of a B-mode video of the porcine spinal cord, and the automatically generated cord mask was generalized to the remainder of the frames. This generalization was successful throughout the 19 minutes of the full video tested, and a 1-minute sample is shown in **Supplementary Video 7**.

Supplementary Section 6
CT Myelograms after MUSIC implantation

Introduction: The safety profile of MUSIC needs to be rigorously evaluated before translating to humans. Fibrosis and tissue growth around an implant can alter the spinal cord environment. For MUSIC, we have conducted a CT myelogram to assess the spinal cord's structural integrity after acute (5-days) and longer-term (15-days) implantation.

Methods: An injury was performed in the acute model but not in the 2-week model. On Day 5 and Day 15, myelography was conducted to confirm device placement and to detect any impairment of cerebrospinal fluid circulation. A lumbar laminectomy was performed at L3-L5 to allow intrathecal access. A lumbar catheter was advanced into the thecal sac under direct visualization. Through the catheter, 10 mL of nonionic iodinated contrast (iohexol, Omnipaque™ 300 mgI/mL) was injected slowly by hand over approximately 60 seconds. Following injection, CT myelography of the lower cervical to lumbosacral spine was performed on a Siemens Somatom 256-slice dual-energy CT scanner. Sagittal, axial and coronal images were obtained and reviewed by a board-certified neurosurgeon for evaluation of device position and spinal canal compression.

Results: Myelography on both Day 5 and Day 15 revealed uniform opacification of the CSF space from the lumbar cistern to the lower cervical region. The device was visualized spanning from T5-T7 in expected position without associated narrowing of the thecal sac or mass effect on the spinal cord. No filling defects, contrast extravasation, or intradural masses were identified. Contrast ascended freely without evidence of obstruction or delay in transit. Overall, the CT myelogram demonstrated normal CSF flow dynamics without evidence of significant cord compression.

Conclusion: The absence of substantial cord compression in these assessments provides preliminary structural tolerability evidence under the tested conditions. These findings support further evaluation of MUSICfor extended temporary implantation.

Supplementary Section 7

Physiological Importance of Blood-Flow Velocity modulation

Common clinical practice aims to increase microvascular blood flow to minimize secondary ischemia after spinal cord injury. We evaludated MUSIC 's ability to measure Doppler-derived blood-flow velocity changes in a localized signal attributable to the sulcal artery region, a vital microvascular region supplying the gray and white matter tracts of the spinal cord.(*40*, *70*) Monitoring flow changes within this anterior vessel requires balancing penetration depth with image resolution, hence why diagnostic ultrasound imaging is used. Spectral doppler resolves the beat-to-beat variation in blood flow dynamics and quantifies the blood flow velocity. The relationship between blood flow velocity and blood flow is governed by the following equation: Equation S1:

$$BF = BFV * A \tag{S1}$$

BF and BFV are blood flow(ml/s) and blood flow velocity(cm/s) respectively, and A is the cross sectional area of the vessel. We acknowledge that contrast agents could track perfusion-adjacent metrics through techniques such as contrast-enhanced ultrasound and ultrasound-localization microscopy. However, these agents not only could induce unintended bioeffects during therapeutic ultrasound administration, but can also elicit an adverse systemic reaction in our porcine studies.(*71*) Hence, we evaluated features of the spectral doppler data to contextualize the blood flow velocity changes observed. Vascular resistance in the sulcal artery was analyzed to determine any association with ultrasound stimulation. Across two FUS sessions, A 45 V input voltage induced the largest change in the resistivity index (-0.07 and -0.05), a metric for resistance to flow. Figure S9 This change in the metric indicates a reduction in the vessel's impedance to flow. The observed changes in BFV and resistivity index are consistent with altered local hemodynamics under the tested conditions. However, these exploratory Doppler-derived measurements do not independently establish vasodilation, increased volumetric blood flow, or improved tissue perfusion.

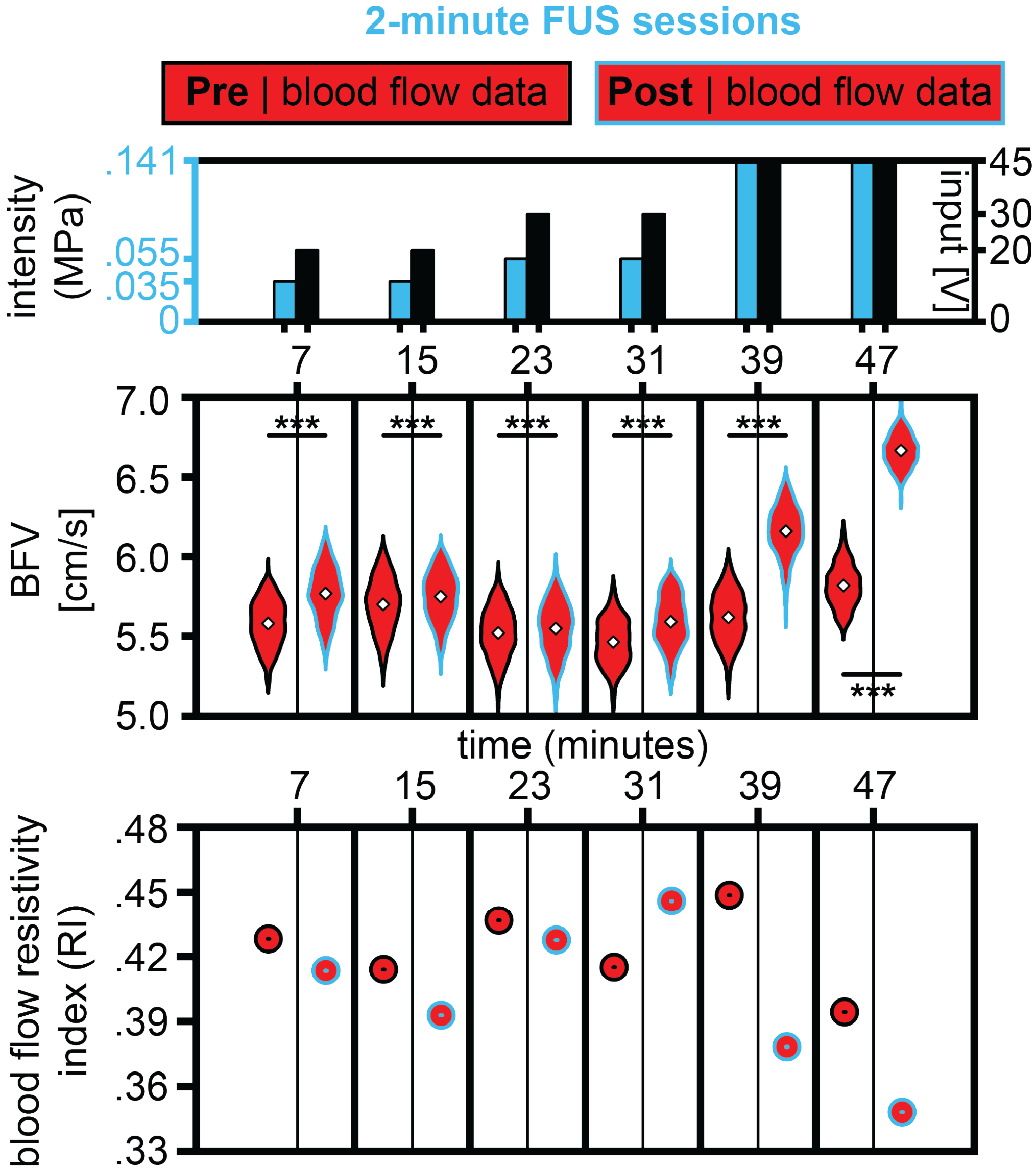

Figure S9: **BFV and RI data from a sequence of exploratory FUS experiments in one pig.** The BFV analysis includes a 30-second window before and after FUS.($^{***}p < 10^{-3}$). BFV is averaged across 10-second windows to limit noise, noting that the BFV is pre-smoothed here following envelope extraction. RI is computed from the raw envelope across 29 cardiac cycles (roughly 30 seconds) before and after therapy. Observed changes in BFV and RI are consistent with altered local hemodynamics but do not independently establish vasodilation, volumetric flow changes, or improved tissue perfusion.

Supplementary Section 8
Supporting hemodynamic modulation information

Ultrasound has demonstrated considerable potential as a non-invasive modality for modulating neural tissue. However, whether effective stimulation parameters generalize across tissue and cell types remains unknown (*72*), which highlights the growing need for a spinal cord-specific platform to identify effective parameters for spinal cord injury. The evaluation of MUSIC's FUS hemodynamic modulation capabilities was performed on a healthy porcine model consisting of three pigs. After being implanted and fixed on a targeted region, various acoustic parameters were tested to investigate whether instantaneous acoustic power, time-averaged power, and/or acoustic energy delivered influenced blood flow within the spinal cord's microvasculature. To this end, duty cycle, input voltage, pulse repetition frequency (PRF), and insonation duration were systematically varied to explore how these individual or combined parameters could be leveraged to modulate blood flow velocity. Input voltage was used to adjust the peak acoustic power generated by the therapeutic arrays, while duty cycle and PRF controlled the time-average acoustic power. Insonation duration, on the other hand, could vary the cumulative energy deposited into the region of interest. Together, these parameters provided a comprehensive framework for optimizing the therapeutic potential of MUSIC.

Three time series data and associated surgical logs were collected during the surgery including Canon spectral, MUSIC imaging data, and arterial line data. From these, we can extract local hemodynamics, spinal cord morphological features, and systemic hemodynamics. Additionally, surgical records detailing drug dosages administered during the procedures were used as a screening indicator to exclude experimental sessions heavily influenced by anesthesia or other medications.

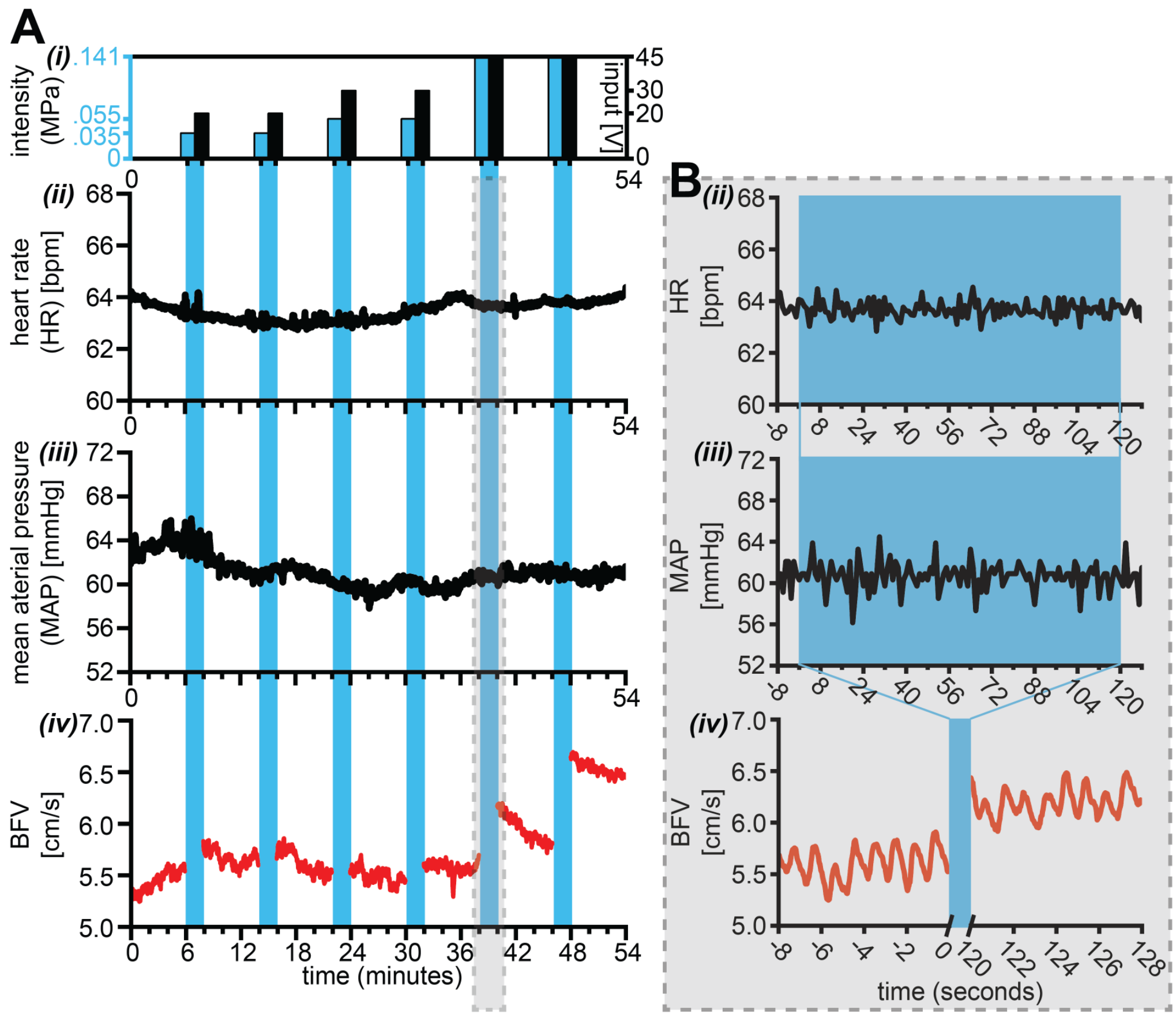

Figure S10: **Sample of a exploratory FUS experiment with MUSIC. (A)** Preliminary proof-of-concept data from six, two-minute therapy sessions are included to indicate that: 1.) systemic physiological function, including heart rate (HR) and mean arterial pressure (MAP), does not appear to be disrupted by the focused ultrasound, 2.) BFV can be modulated and appears to be dependent on the therapeutic intensity or cumulative dose (i). Across the 54 minutes, HR (ii), MAP (iii), and BFV (iv) are averaged across 10-second windows to limit noise, noting that the BFV is pre-smoothed here following envelope extraction.**(B)** Data from the fifth FUS session is shown to illustrate the quality and validity of the signals acquired. The duty cycle within the FUS sessions was 35%. For more information on the FUS parameters used, see row 2 of Table 1.

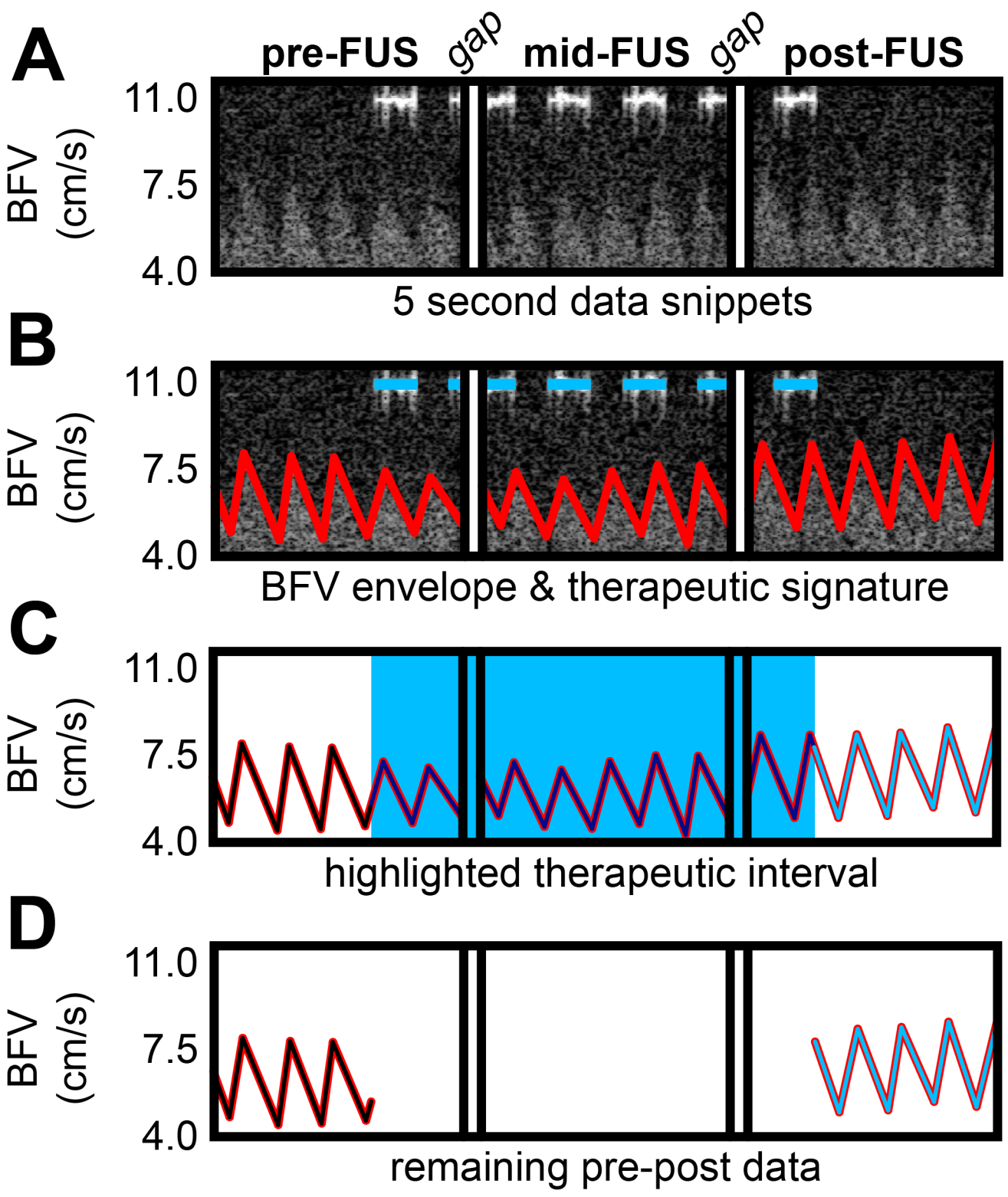

Figure S11: **Illustrative BFV data pipeline.** (**A**) Three 5-second snippets of spectral Doppler data from the commercial ultrasound system are shown. (**B**) Hand tracing highlight the envelope of BFV data in red, and FUS pulses in blue which confirm FUS delivery. (**C**) The spectrogram images are removed, revealing the illustrative data framework. (**D**) The remaining illustrative red envelopes depict the pre- and post- FUS BFV data endpoints in black and blue, respectively.

Each pig surgical experiment consisted of multiple sections, with each section typically comprising six 2-minute sessions or a single 10-minute session. Between sections, recovery time (6-10 minutes) was provided to allow the pig to regain sensitivity to stimulation and to assess its status before starting the next section. This approach ensured that the effects of stimulation could be consistently evaluated across multiple trials.

Each session, the smallest unit of the experiment, included two baseline recordings, referred to as FUS-off periods, taken before and after the FUS-on period. These FUS-off periods served as both the pre-FUS baseline and the post-FUS recovery monitoring for the preceding session. However, due to the limited experimental window available for pig surgeries, the intersession recovery periods may not have been ideally long. A 20-second sampling window was applied to capture the portion of the FUS-off periods immediately before and immediately after FUS application.

While there were minimal differences in blood velocity changes between the 20 V (0.035 MPa) and 30 V (0.055 MPa) input voltage cases, there were significantly greater increases in blood flow when 45 V (0.14 MPa) input voltage was used Figure S10A-B. This change may indicate a minimum voltage threshold to elicit significant changes, or this increase may be due to the cumulative energy deposition of FUS over several previous trials.

In cases where the FUS harmonic pattern did not overlap with the main BFV waveform in the spectrogram, we analyzed the blood flow behavior during FUS Figure S11A-B. Interestingly, we observed a transient decrease in blood velocity while FUS was on, followed by the increase in flow after FUS was turned off Figure S11C-D. This phenomenon introduces the possibility of both positive and negative hemodynamic modulation.

The duty dose control section in Figure S12A showed a statistically significant increase in blood velocity when the burst length increased from 0.15 ms to 0.4 ms. The duration dose control section revealed a more pronounced increase in blood velocity after a fivefold longer FUS stimulation at 10 minutes (Figure S12B). As shown in Figure S12C, the pressure dose control section demonstrated a statistically significant increase in blood velocity changes for high-pressure, versus low- and mid-pressure, settings. This finding highlights the possibility of a threshold effect, where a certain pressure level may be required to induce more pronounced modulatory effects. All results were assessed for statistical significance using the Kruskal-Wallis test.

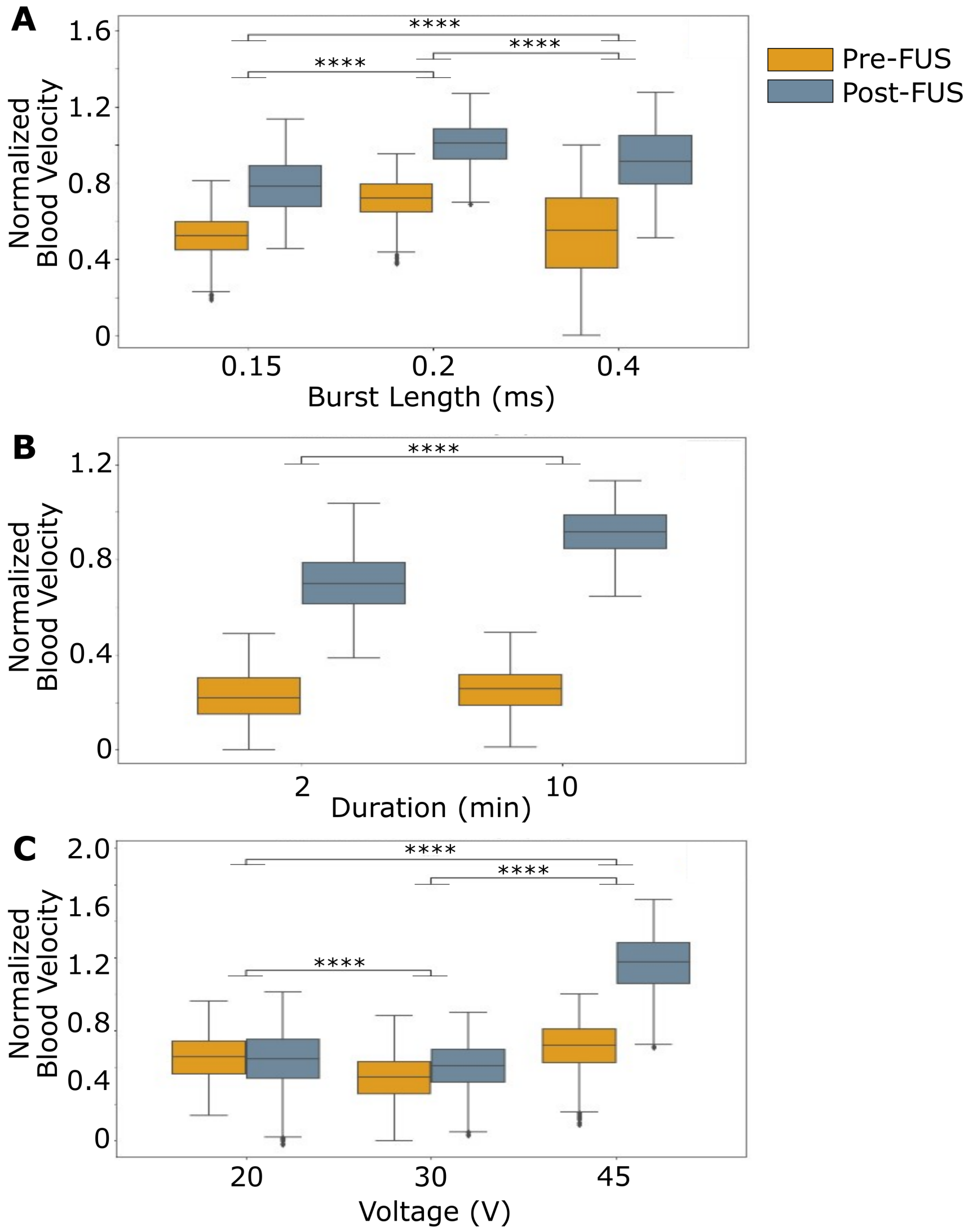

Figure S12: **The hemodynamic response is dependent on the input FUS parameters. (A)** Normalized blood velocity pre- and post-FUS delivery at varying burst lengths, **(B)** duration, and **(C)** input voltage. For more information on the focused-ultrasound parameters, see Table 1.

Supplementary Section 9

Exploratory Immunohistochemistry quantification for MUSIC

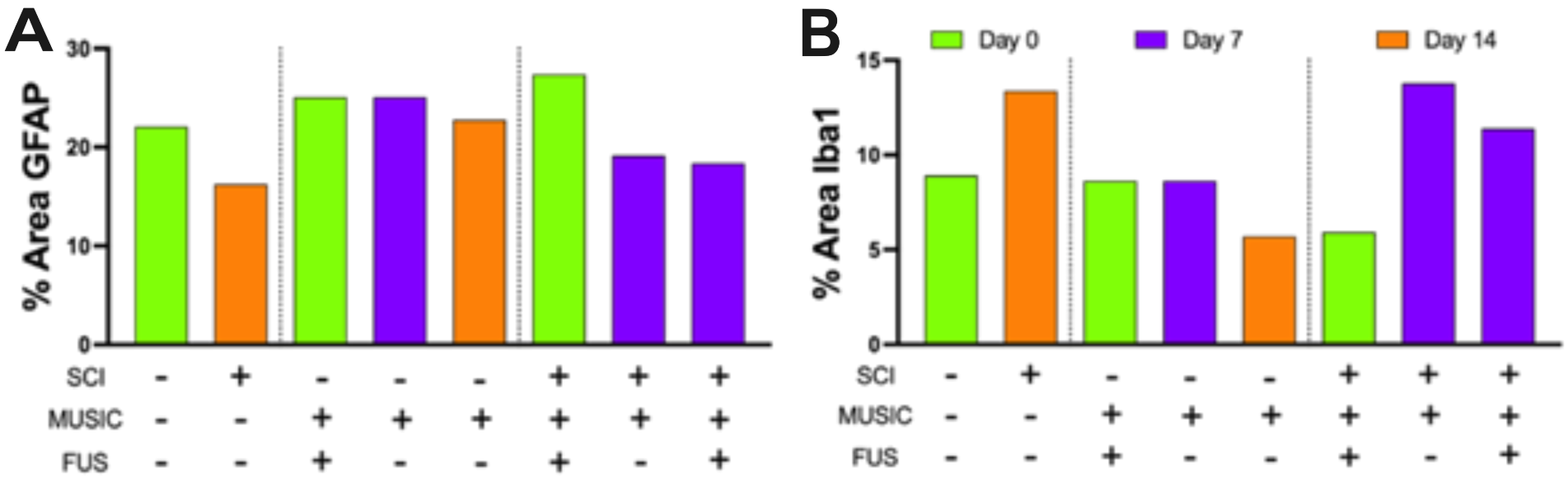

Figure S13: **Exploratory immunohistochemistry quantification for MUSIC .** Percent-area analysis ([stained area/spinal cord area] $\times$ 100) of representative IHC images from Figure 6 shows **(A)** GFAP, an astroglial marker, and **(B)** Iba1, a macrophage/microglial marker. In this $N = 2$ pigs/group cohort, uninjured MUSIC -implanted specimens were compared descriptively with naïve controls, while SCI specimens showed increased Iba1 signal and reduced GFAP signal in the analyzed sections. These measurements are presented as descriptive histologic context and should be interpreted alongside the matched H&E and Masson's trichrome morphology rather than as definitive tissue-safety inference. Analysis performed via ImageJ.

Supplementary Section 10
Closed-loop therapy concept

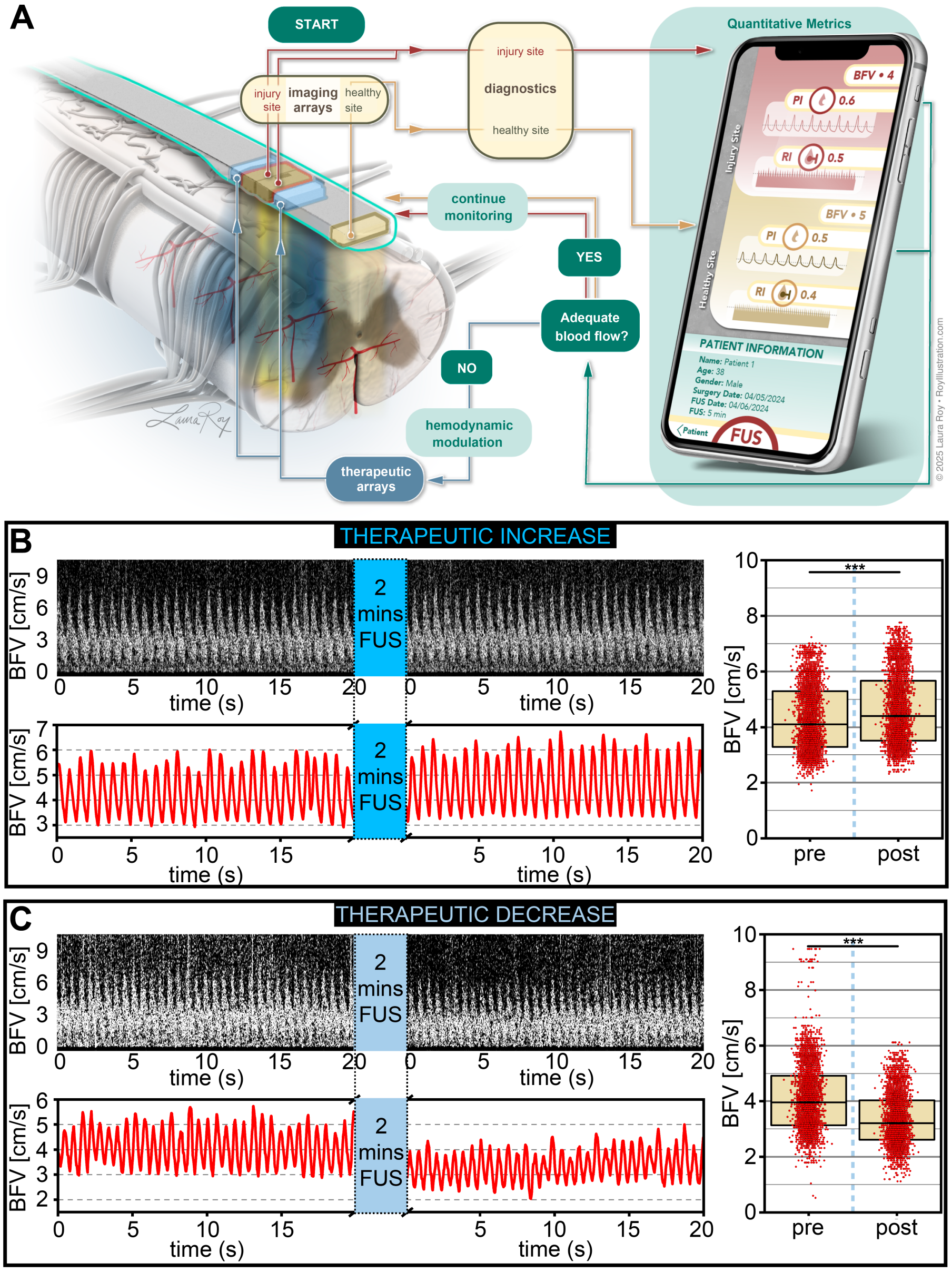

Figure S14: **Demonstrating the potential of sensing-informed FUS with MUSIC . (A)** A schematic depicts the potential deployment of a future closed-loop, theranostic platform. **(B-C)** Experimental data demonstrate preliminary sensing-informed hemodynamic modulation, showing that BFV changes to the sulcal artery of interest can be induced and tracked for both increases and decreases ($^{***}p < 10^{-3}$). Raw spectrograms and its corresponding envelopes are plotted. Spectrograms have a modified brightness range and the BFV envelope is smoothed across 0.25-second windows for clear visualization purposes.

Supplementary Section 11
Acoustic field simulations

Here we present 3-dimensional numerical simulations modeling the acoustic output of the MUSIC imaging and therapeutic arrays, which we performed with OnScale (OnScale Inc., GA, USA). These simulations provide insight into how the acoustic pressure fields perform in realistic conditions, including the presence of additional fabrication layers such as acoustic lenses used for imaging. The simulations were performed for one-way imaging (transmit-only), two-way imaging (transmit and receive), and therapeutic ultrasound (transmit-only). The purpose of these simulations was to validate the acoustic performance observed experimentally.Furthermore, this section connects the theoretical derivation of the acoustic output later described in Supplementary Section 12 to the fabrication methods described in the main text and Supplementary Sections 1-3 and performance optimization for MUSIC.

The results in each of the following subsections are presented as time-domain signals, frequency-domain signals (calculated via discrete Fourier transform), directivity patterns, and pressure fields. These figures provide an extensive assessment of MUSIC's acoustic performance. Integrating numerical simulations, theoretical derivations, and experimental validations provides a deep understanding of how MUSIC operates, as well as how design and fabrication could be modified for future clinical usage.

The following acoustic field simulations are presented as interpretive and exploratory models intended to provide qualitative insight into the system behavior, spatial interactions, and underlying design rationale. They are not intended to represent fully resolved real-world acoustic conditions or definitive physical outcomes. Actual system performance may vary due to environmental influences, material properties, boundary conditions, nonlinear effects, manufacturing tolerances, and other practical factors not fully captured within the simulation framework.

11.1 Imaging, High-Frequency (10.5 MHz) Arrays

11.1.1 Simulation, One-Way Pulse Capture

The one-way imaging simulations provide the acoustic fields generated by a MUSIC imaging array during transmission. We performed the transmission-only tests to evaluate the pressure amplitude and the spatial spread of energy observed at the focal point (Figure S15-Figure S17).

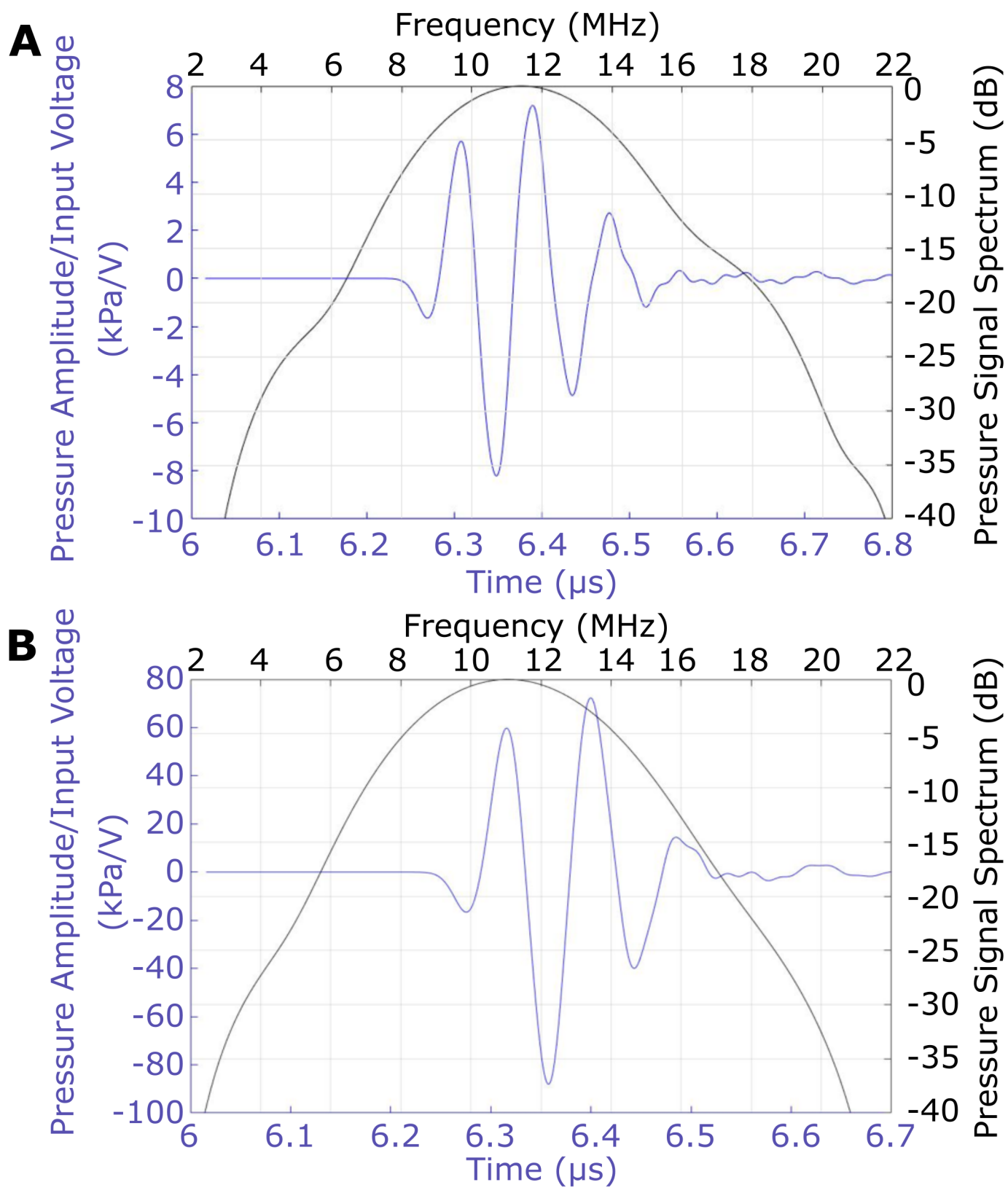

Figure S15: **One-Way pressure response. (A)** Time-domain pressure signal at the expected 9 mm focal point, and its frequency spectrum, resulting from a single-element sine wave excitation and **(B)** a plane-wave sine wave across the array.

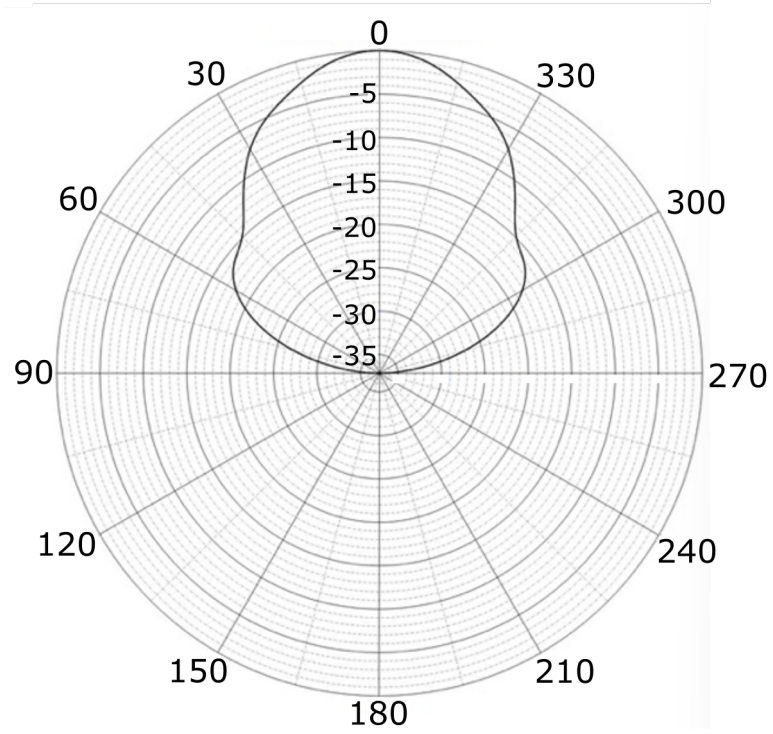

Figure S16: **Directivity pattern for a single-element transducer**, demonstrating the radial pressure magnitude response. The -6 dB directivity angle is $\pm 27°$. Of particular interest is the lack of side lobes in the imaging array, resulting in the energy focus at the locations of interest.

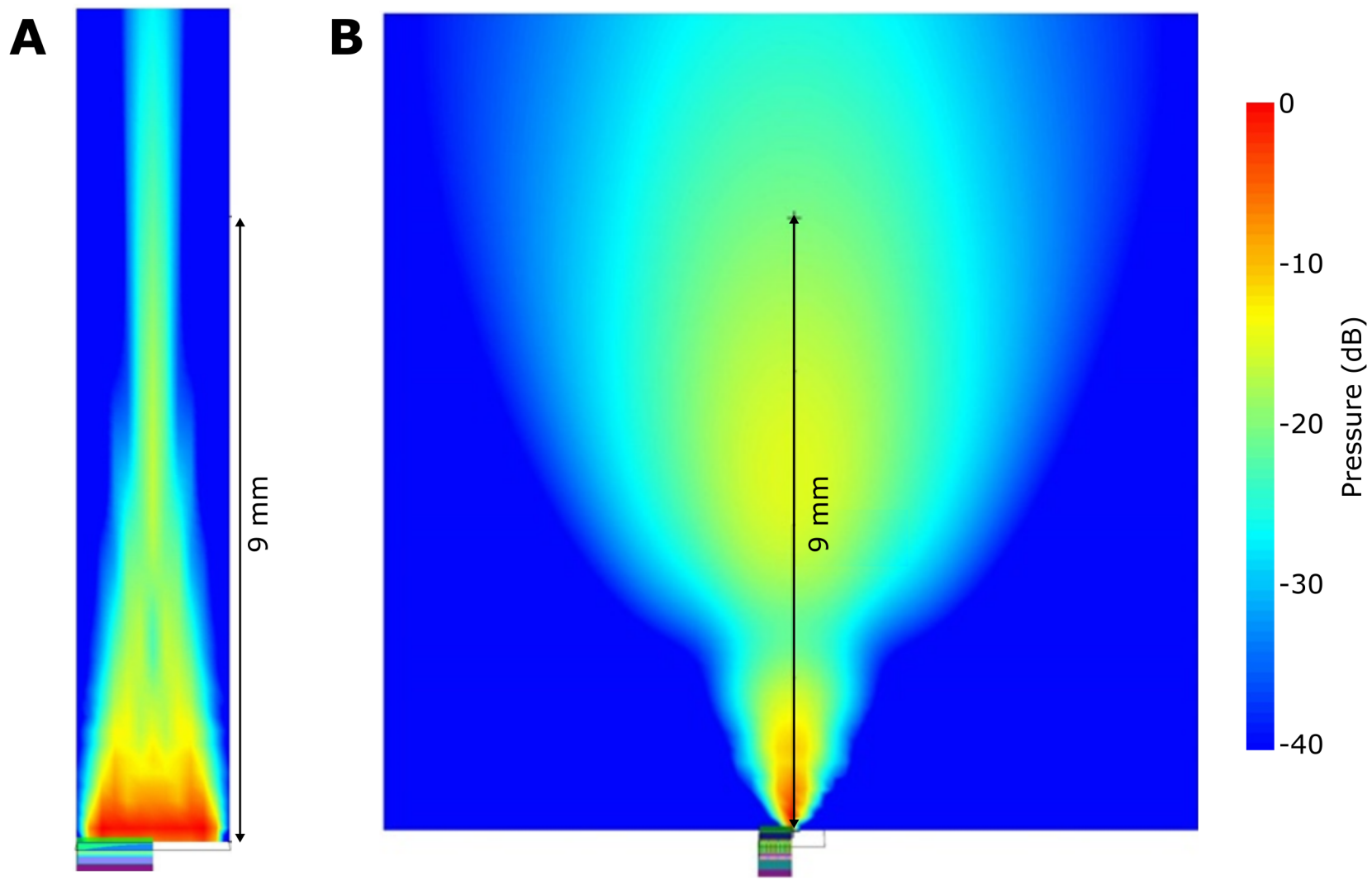

Figure S17: **Pressure field plot** in the **(A)** elevation and **(B)** azimuth planes obtained from single-element excitation.

11.1.2 Simulation, Two-way Pulse Capture

We performed two-way imaging simulations to assess MUSIC's imaging performance during transmit and receive, providing a more realistic diagnostic imaging scenario where image reconstruction is done by doing continuous two-way (i.e., both transmitting and receiving) . These simulations provide a theoretical benchmark for the sensitivity and acoustic efficiency of MUSIC imaging (Figure S18).

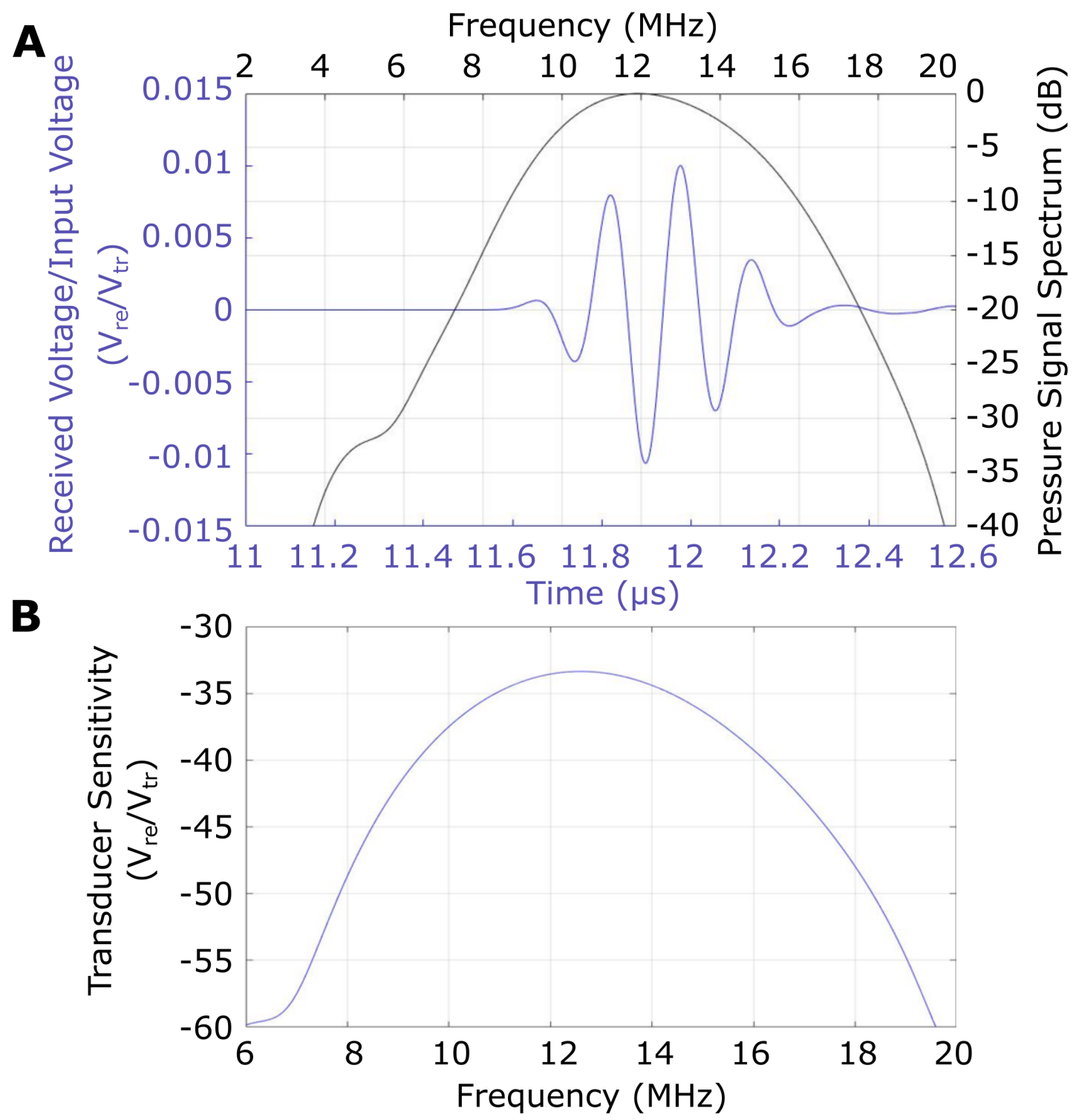

Figure S18: **Simulated Pulse echo plot. (A)** 2-way pulse-echo received voltage and its frequency spectrum, resulting from a single-element sine-wave excitation and a reflector placed at a 9 mm distance. **(B)** Sensitivity plot (output-to-input voltage ratio) of the transducer.

11.1.3 Experiment, Two-Way Pulse Capture

An experimental study was carried out on the prototyped transducer, in which the reflector was positioned 9 mm away from the transducer. The Results for the 2-way pulse-echo signal received from four individual elements are shown in Figure S19. The reflector was positioned 9 mm away from the transducer. The close agreement of amongst four representative elements shows the repeatability and producibility of the transducer design.

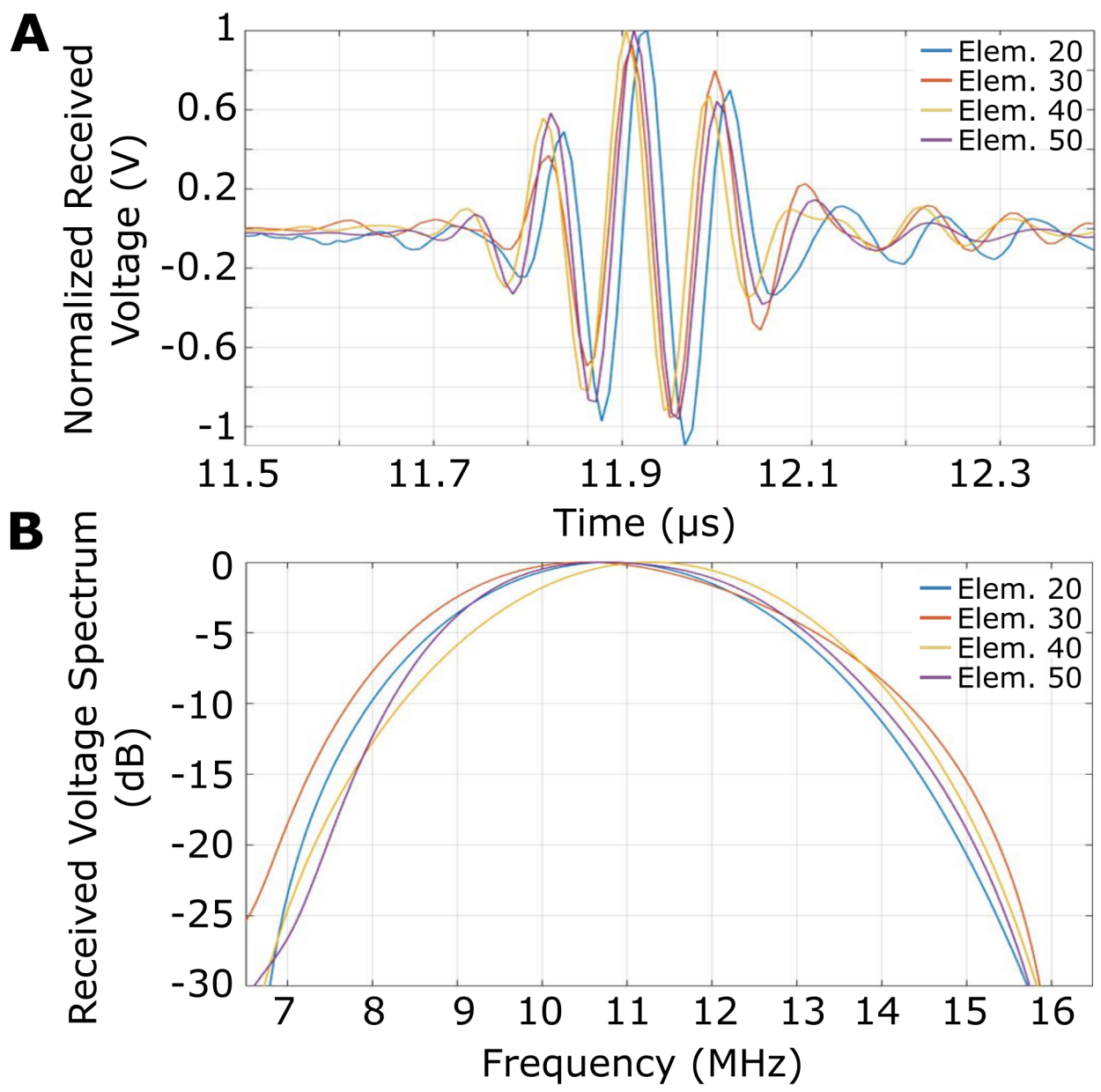

Figure S19: **Simulated Pulse echo plots. (A)** 2-way pulse-echo received signal, resulting from a single-element sine wave excitation. **(B)** Frequency spectrum of the pulse-echo received signal from a single-element sine wave excitation. Each line represents a different element across the imaging array.

11.1.4 Simulations Informed the MUSIC Design

To relate the acoustic stack design to the fabricated MUSIC device, we compared the simulated response with the experimentally captured two-way pulse-echo response from single-element excitation (Figure S20). These simulations were used as an approximate design tool to guide stack selection, define a target operating band, and inform the MUSIC build, rather than as a direct reproduction of the final fabricated device response. The measured response showed operating-band behavior, while the observed differences in bandwidth and center frequency, including a center-frequency shift within approximately 15 percent, are consistent with practical fabrication and measurement effects. These include fabrication tolerances, kerf and cut variation, epoxy/casting/loading layers, backing and packaging boundary conditions, interconnect parasitics, crosstalk, ringing, and measurement-setup differences that are not fully captured in the idealized simulation model. Therefore, acceptance of the final MUSIC design was based on post-fabrication experimental characterization, including impedance, pulse-echo testing, phantom imaging, Doppler testing, acoustic-output assessment, and in vivo deployment. Collectively, these measurements demonstrate that the final MUSIC device was functional and performed as intended for its spinal cord deployments.

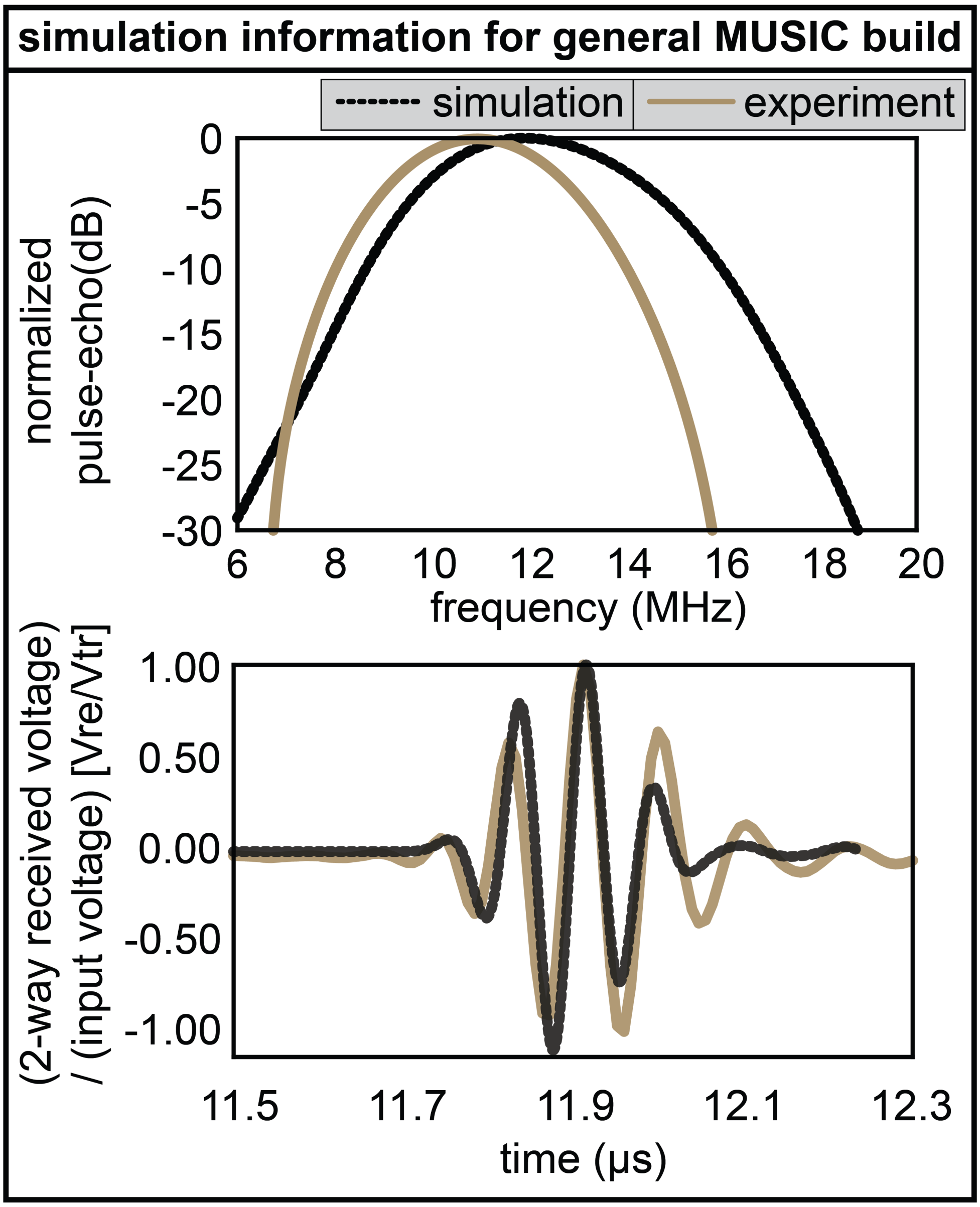

Figure S20: **Simulation-guided comparison of MUSIC pulse-echo response.** Simulated and measured MUSIC pulse-echo sensitivity and frequency-spectrum responses are shown for single-element excitation. The simulations were used as approximate design guidance to help define a target operating band and support selection of the MUSIC build, rather than to directly reproduce the complete response of the fabricated device. Differences between simulated and measured responses are expected from fabrication tolerances, kerf and cut variation, epoxy/casting/loading layers, backing and packaging boundary conditions, interconnect parasitics, crosstalk, ringing, and measurement-setup differences. Final MUSIC performance was established experimentally through impedance, pulse-echo testing, phantom imaging, Doppler testing, acoustic-output assessment, and in vivo deployment, demonstrating that the device was functional and performed as intended for spinal cord deployment.

11.2 Therapeutic, Low-Frequency (2.5 MHz) Arrays

11.2.1 Simulation, One-Way Pulse Capture

We performed simulations to evaluate the acoustic performance of the therapeutic ultrasound arrays (Figure S21-Figure S23).

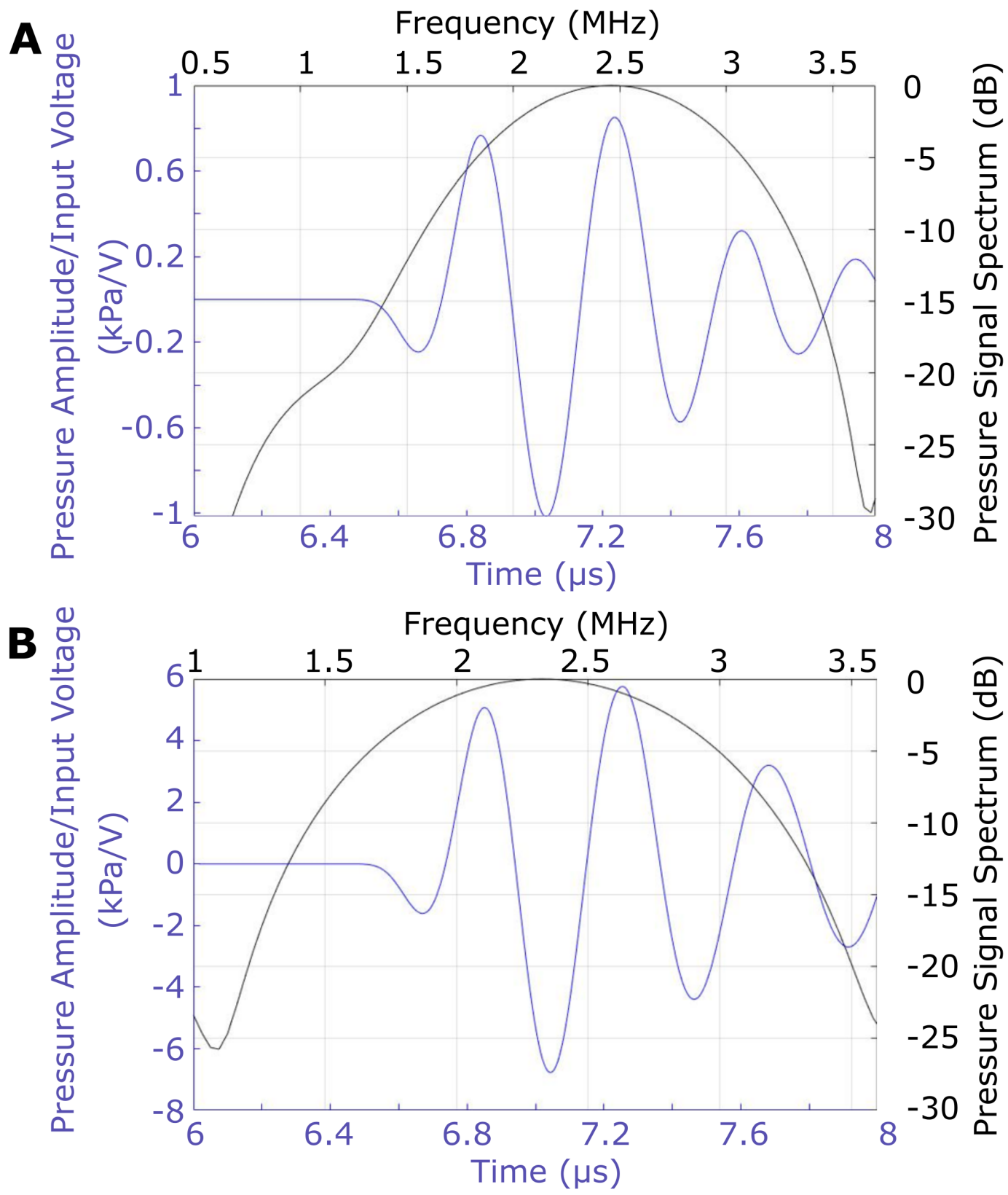

Figure S21: **One-way pressure response. (A)** Time-domain signal and its frequency spectrum at 9 mm for a single-element sine wave excitation and **(B)** plane-wave sine wave excitation across the array at 2.5 MHz.

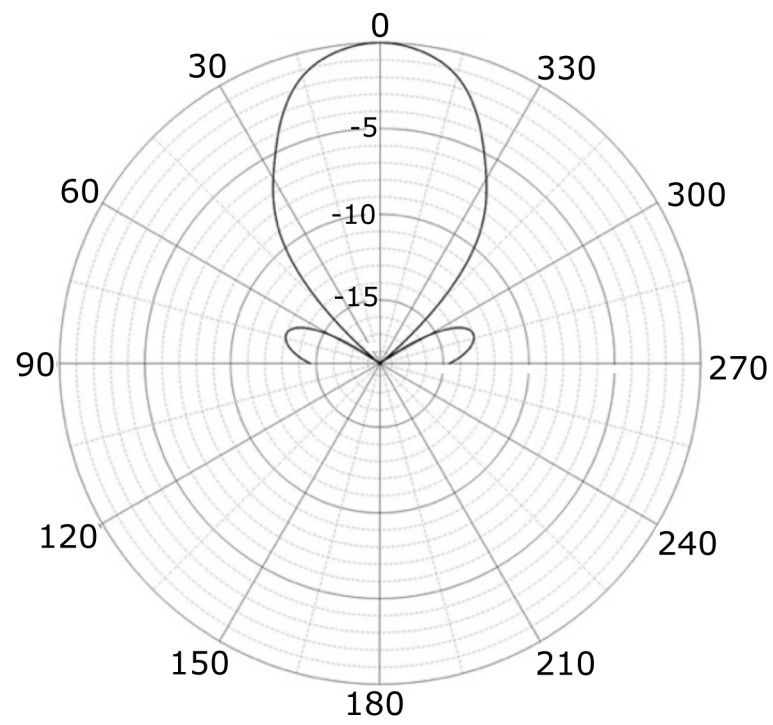

Figure S22: **Directivity pattern for single-element excitation at 2.5 MHz, illustrating a radial pressure response.** The -6 dB directivity angle is $\pm 30°$.

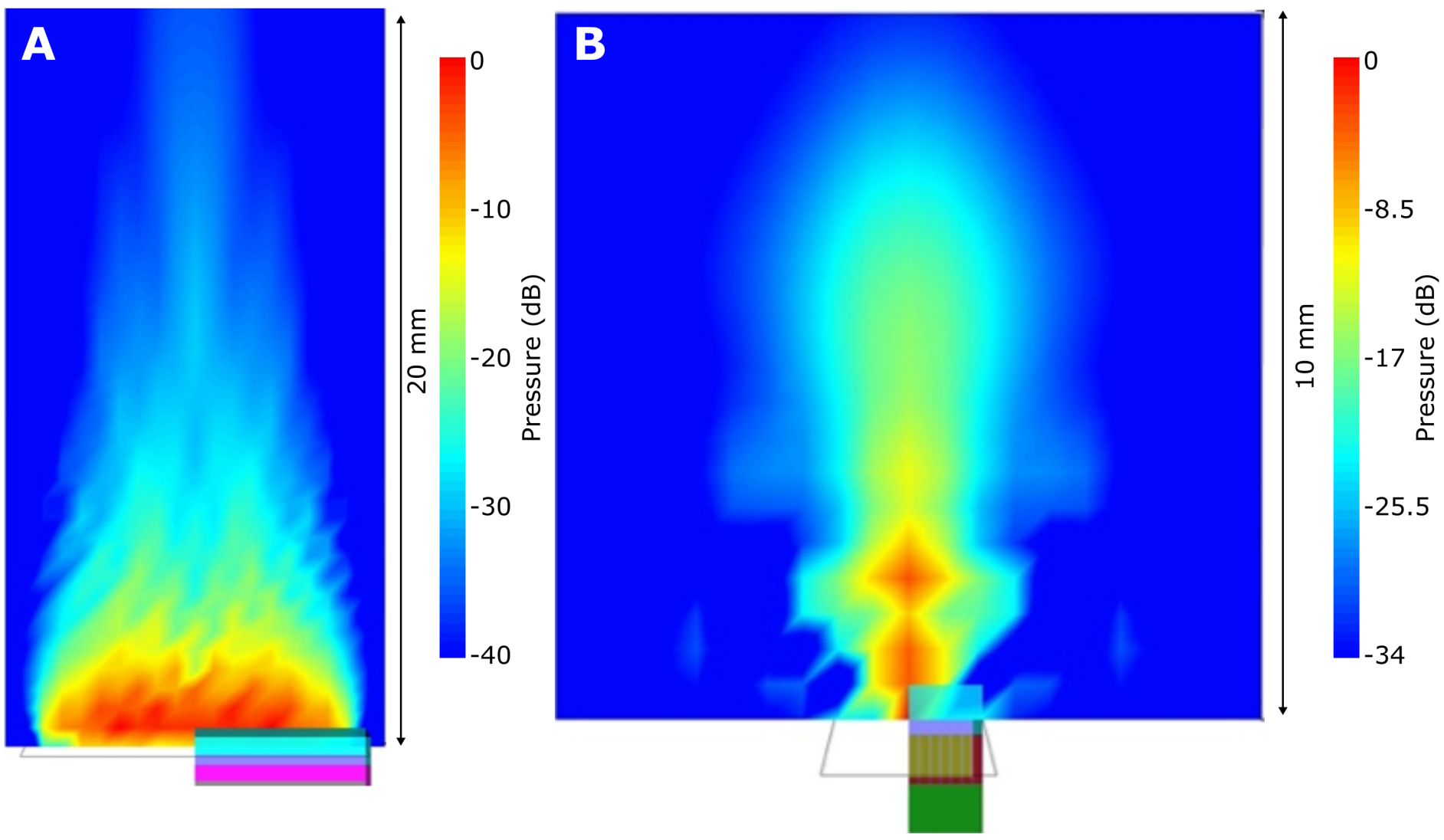

Figure S23: **Simulated pressure plots for a single element.** **(A)** Elevation and **(B)** azimuthal pressure field plots from a single-element excitation, showing the 2D distribution in the therapeutic range.

11.2.2 Simulation vs. Experiment, Two-Way Capture

To verify the therapeutic array simulation results, the two-way pulse-echo obtained from both simulations and experiment were compared. The therapeutic element was half activated for the experimental results (Figure S24).

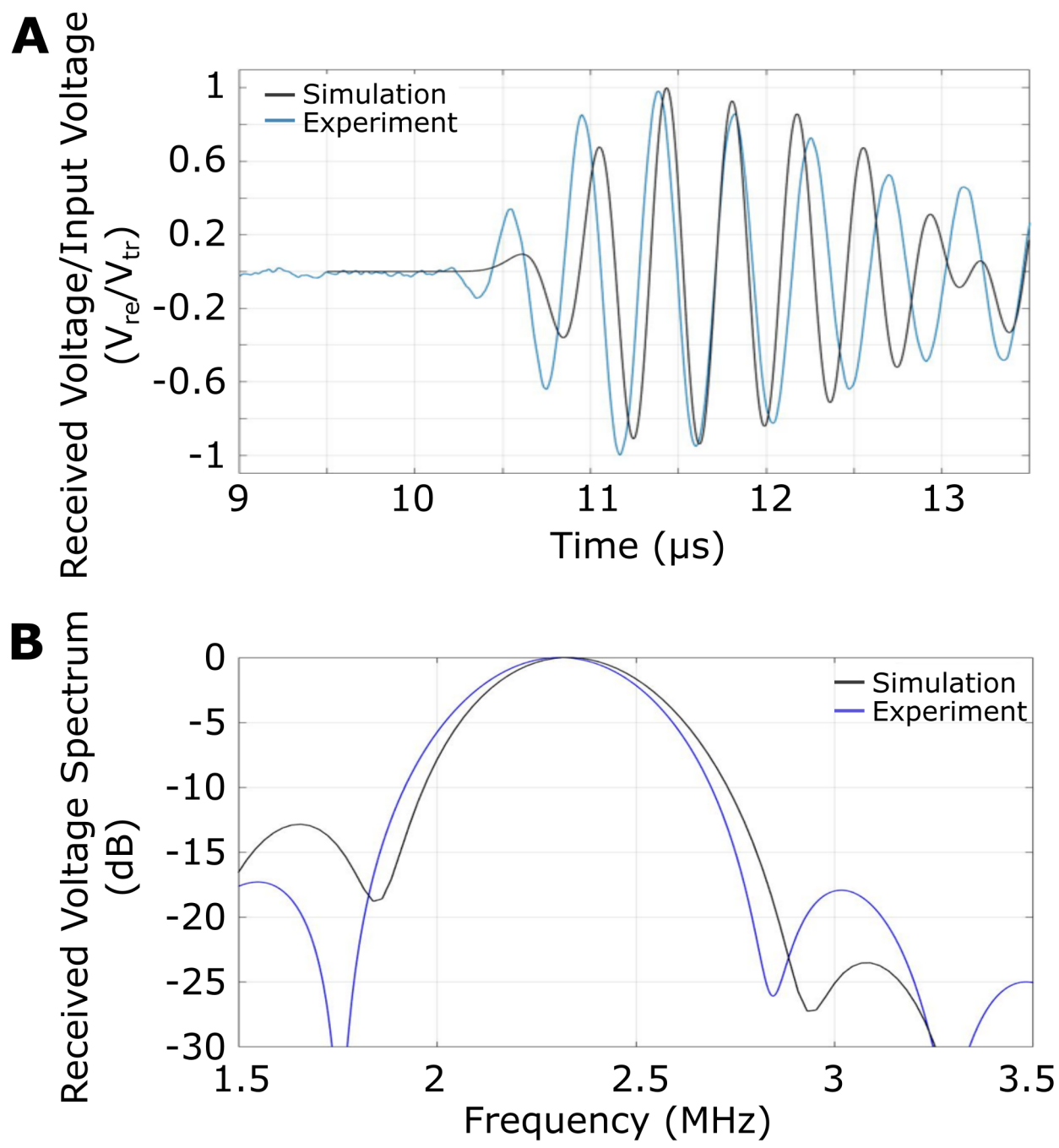

Figure S24: **Comparing experimental pulse-echo data to simulation results. (A)** 2-way pulse-echo sensitivity and **(B)** frequency spectrum comparison between simulation and experiment received from a single-element excitation of the therapeutic arrays.

Supplementary Section 12
Mathematical modeling and driving equations

Here we derive a formula using the Rayleigh-Sommerfeld diffraction integral to describe MUSIC's therapeutic and imaging acoustic pressure outputs. We provide detailed equations for on-axis and off-axis acoustic pressure fields, in the presence of multiple parallel imaging and therapeutic ultrasound arrays.

The following equations are presented as conceptual and analytical frameworks intended to illustrate the reasoning, assumptions, and design methodology underlying the system architecture. They should be interpreted as guiding approximations rather than exhaustive representations of real-world physics, and are not claimed to constitute complete or definitive theoretical models. As with all complex physical systems, practical implementation is influenced by additional variables, nonlinearities, material constraints, and empirical considerations beyond the scope of the simplified formulations presented here.

12.1 On-axis, 1-dimensional pressure profile for one, 8-element therapeutic array

12.1.1 Rayleigh integral setup

The ultrasound pressure field at a point, $\mathbf{r} = (x, y, z)$, is given by the Rayleigh-Sommerfeld diffraction integral (Equation S2) (*73*):

$$p(\mathbf{r}) = \frac{j\rho\omega}{2\pi} \int_S \frac{v(\mathbf{r}')e^{-jk|\mathbf{r}-\mathbf{r}'|}}{|\mathbf{r}-\mathbf{r}'|} dS', \tag{S2}$$

where ρ is the propagation medium density, ω is the angular frequency of the transducer, k is the wavenumber, $\mathbf{r}' = (x', y', z')$ is a source point on the array surface, and $v(\mathbf{r}')$ is the surface velocity of the transducer elements at point $\mathbf{r}'$. ω and k are calculated according to Equation S3-Equation S4, respectively.

$$\omega = 2\pi f, \tag{S3}$$

$$k = \frac{\omega}{c}, \tag{S4}$$

where c is the propagation medium speed of sound, and f is the center frequency of the transducer. Given that the therapeutic and imaging arrays have different center frequencies (f_t and f_i, respectively), k takes the form of either k_t or k_i in the case of therapeutic or imaging arrays, respectively.

Given that MUSIC's pressure output was measured in a water tank, $\rho = 1000$ kg/m^3 and $c = 1500$ m/s. We assume a uniform surface velocity (i.e., $v(\mathbf{r}') = v_0 = 1$ cm/s) over the aperture surface.

12.1.2 Numerical integration methodology

We approximate the pressure integral by discretizing with the trapezoidal rule over the individual transducer elements, given by Equation S5:

$$p(\mathbf{r}) \approx j\rho f \sum_{n=1}^{N} \frac{v_0 e^{-jk|\mathbf{r}-\mathbf{r}_n|}}{|\mathbf{r}-\mathbf{r}_n|} \Delta n, \tag{S5}$$

where N is the number of discrete points (i.e., the number of transducer elements), and Δn is the area of the discrete element (i.e., the width of each transducer element).

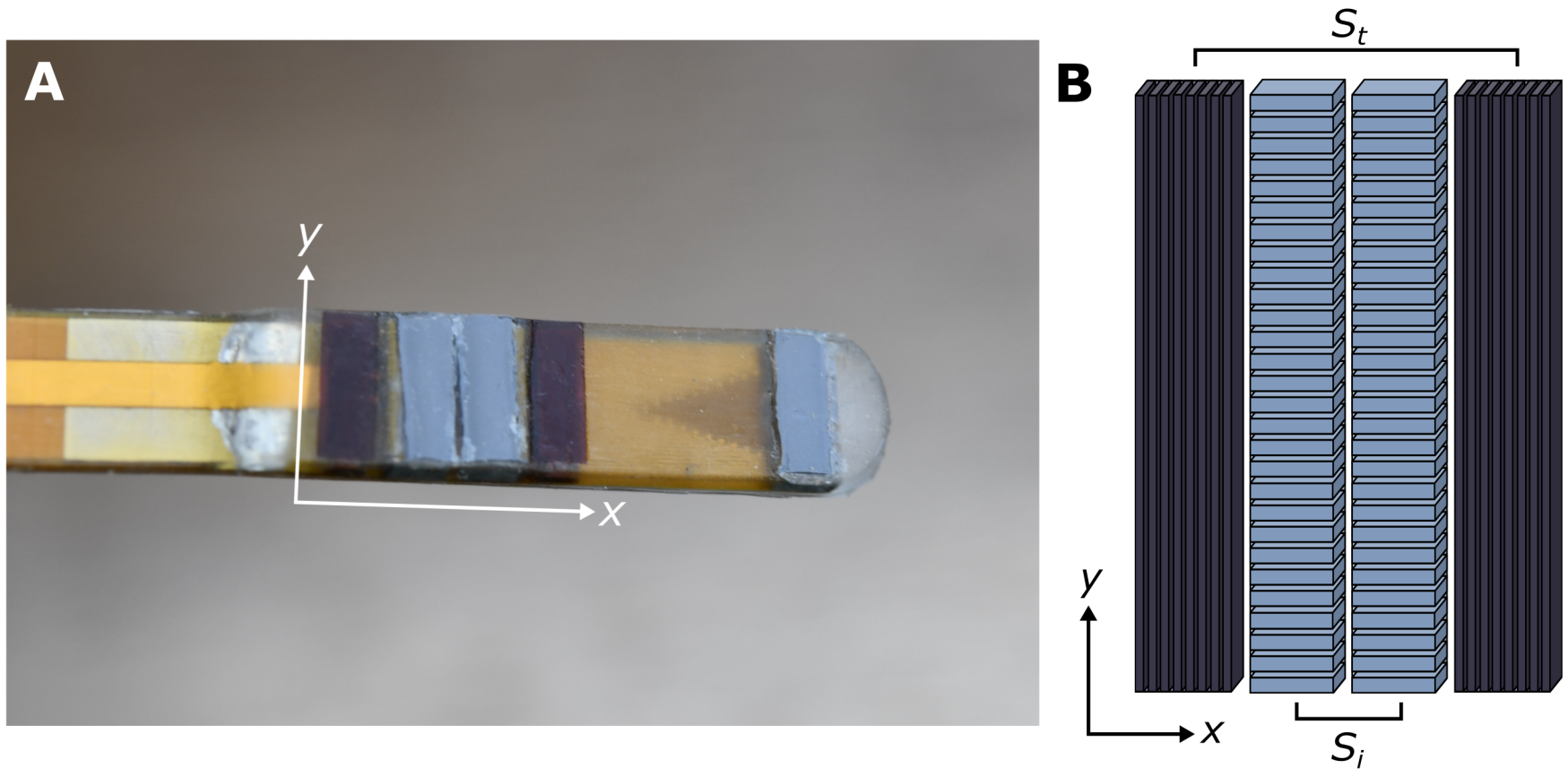

Figure S25: **Illustrative coordinate system for MUSIC. (A)** Photograph and **(B)** schematic of the X and Y orientation of the imaging and therapeutic arrays of MUSIC. The Z-axis is normal to the surface of the transducer. S_i and S_t refer to the center-to-center distance between the imaging arrays and therapeutic arrays, respectively.

12.1.3 Geometry and boundary conditions

The orientation of the arrays and elements for the therapeutic and imaging arrays are defined in Figure S25. The therapeutic arrays are oriented along the x-axis, with the elements concurrently arranged along the x-axis (Figure S25B).

The following specifications are provided for a single therapeutic array:

- Element pitch: 300 μm
- Element width, Δx_n: 200 μm
- Element kerf: 100 μm
- Frequency, f_t: 2.5 MHz
- Number of elements, N_t: 8

12.1.4 On-axis pressure calculation

When the field position is on-axis, directly in front of the center of the array, $\mathbf{r} = (0, 0, z)$. Given that the individual elements are oriented along x, the source position is given by $\mathbf{r}_n = (x_n, 0, 0)$. Thus $|\mathbf{r} - \mathbf{r}_n|$ is simplified as follows:

$$\begin{aligned} |\mathbf{r} - \mathbf{r}_n| &= \sqrt{(x - x_n)^2 + (y - y_n)^2 + (z - z_n)^2} \\ &= \sqrt{x_n^2 + z^2} \end{aligned} \tag{S6}$$

Thus, the on-axis, 1D pressure for a single therapeutic array is given by Equation S7:

$$p(z) = j\rho f_t \sum_{n=1}^{N_t} \frac{v_0 e^{-jk_t\sqrt{x_n^2+z^2}}}{\sqrt{x_n^2 + z^2}} \Delta x_n, \tag{S7}$$

where x_n is the center position of each element and Δx_n is the width of each element.

Assuming the elements are symmetric about $x = 0$, the center position of each element is given by Equation S8:

$$x_n = \left(n - \frac{N_t - 1}{2}\right) \times \text{pitch}, \tag{S8}$$

where n ranges from 0 to (N_t-1) (i.e., the index of the array element). Therefore, $x_n = [-1.05, -0.75, ..., 0.75, 1.05]$ mm.

12.1.5 Numerical results and discussion

We multiplied the theoretical pressure by a 10^{-6} term to provide an estimate of pressure in MPa; however, given the assumption of a surface velocity v_0 of 1 m/s, we present the relative pressure in arbitrary units (Figure S26-Figure S28). These plots show the acoustic pressure output's real, imaginary, and magnitude components. Figure S27 and Figure S28 illustrate the squared pressure output, as p^2 is directly proportional to the acoustic intensity (*73*). Figure S28 shows the squared pressure on a logarithmic scale to better expose the variability in pressure values across different depths.

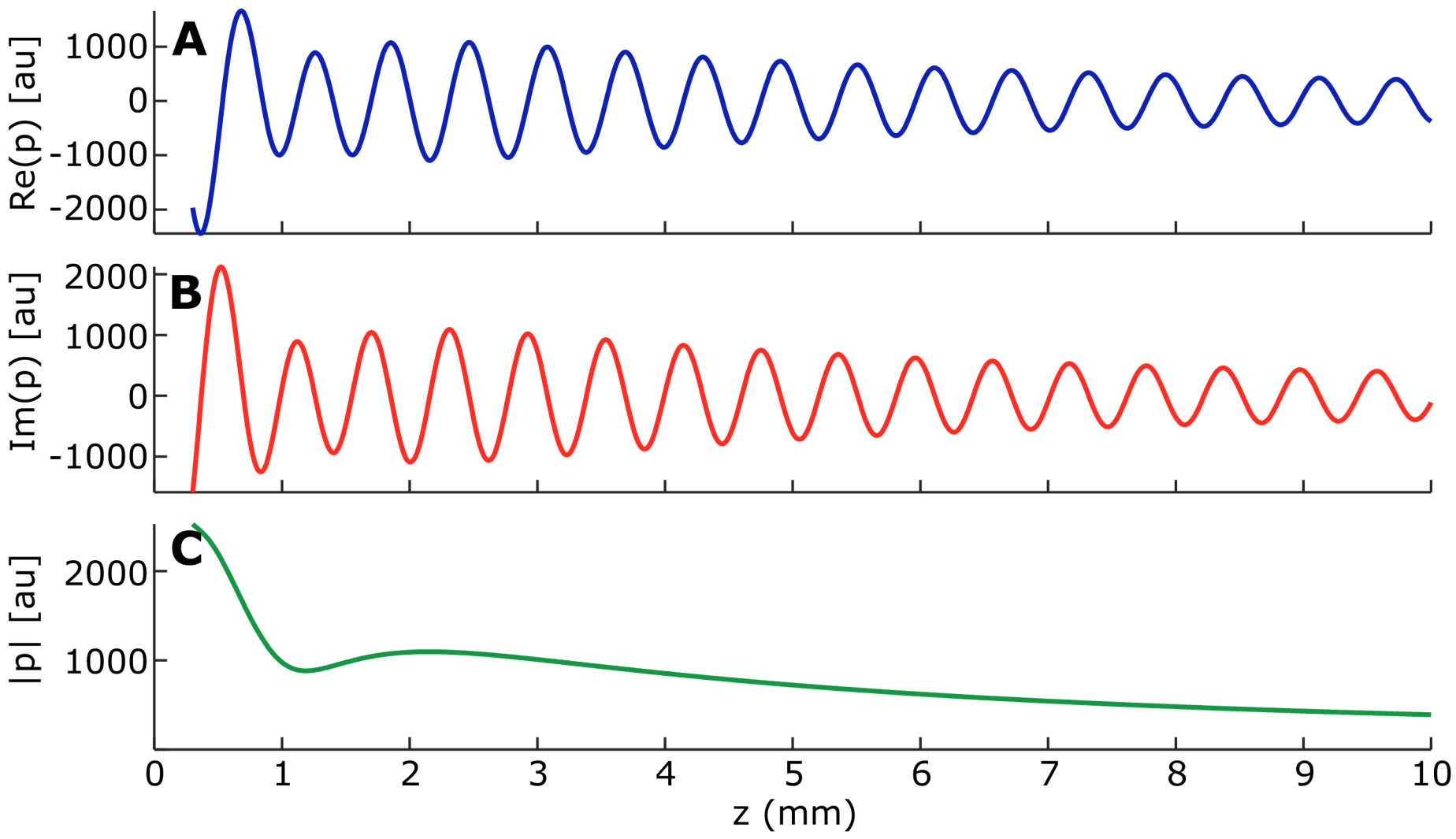

Figure S26: **Pressure versus distance for the therapeutic array. (A)** Real component, **(B)** imaginary component, and **(C)** the magnitude of the pressure emitted from the one therapeutic array on-axis.

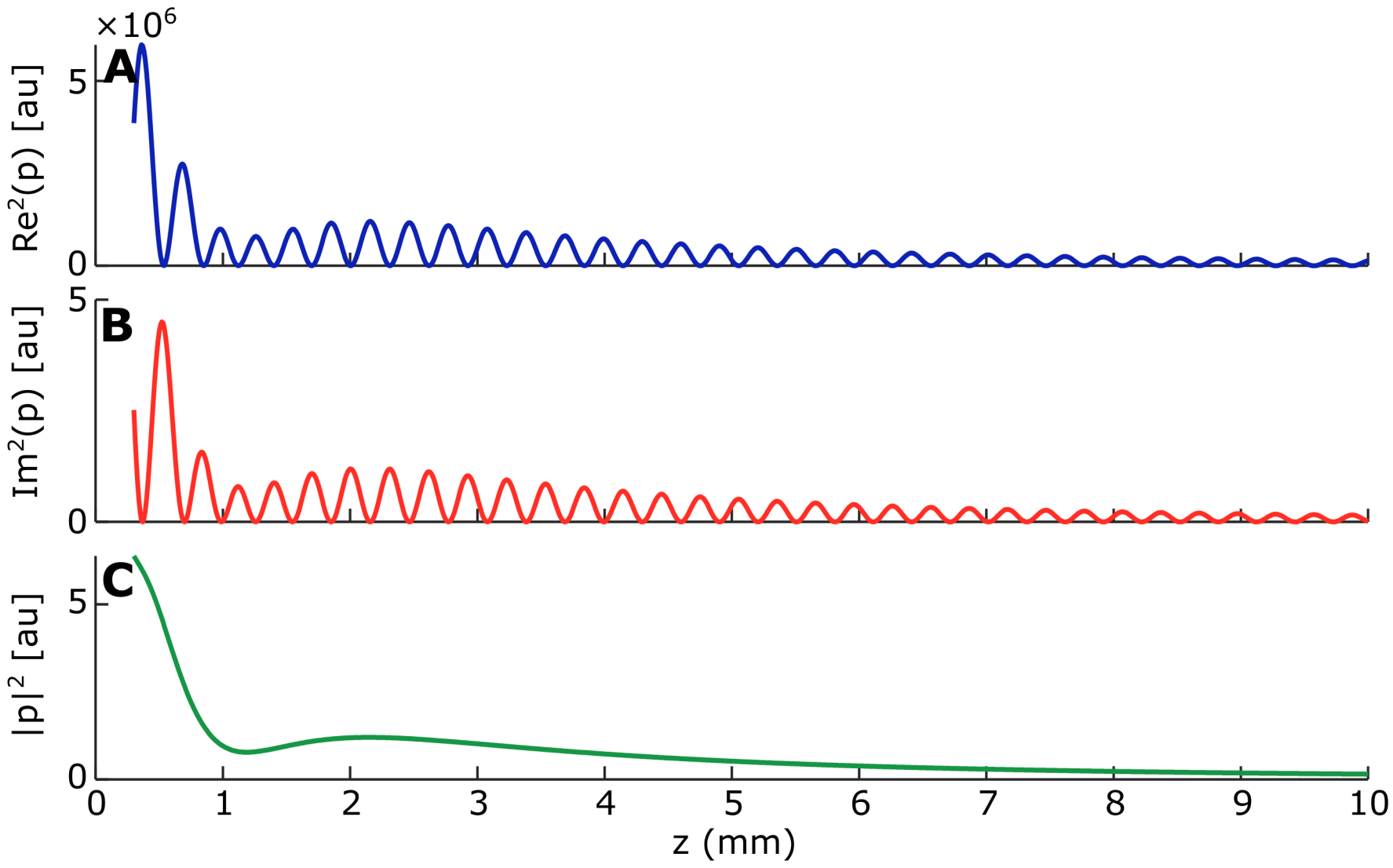

Figure S27: **Squared pressure versus distance for the therapeutic array. (A)** Real component, **(B)** imaginary component, and **(C)** the magnitude of the squared pressure emitted from the one therapeutic array on-axis.

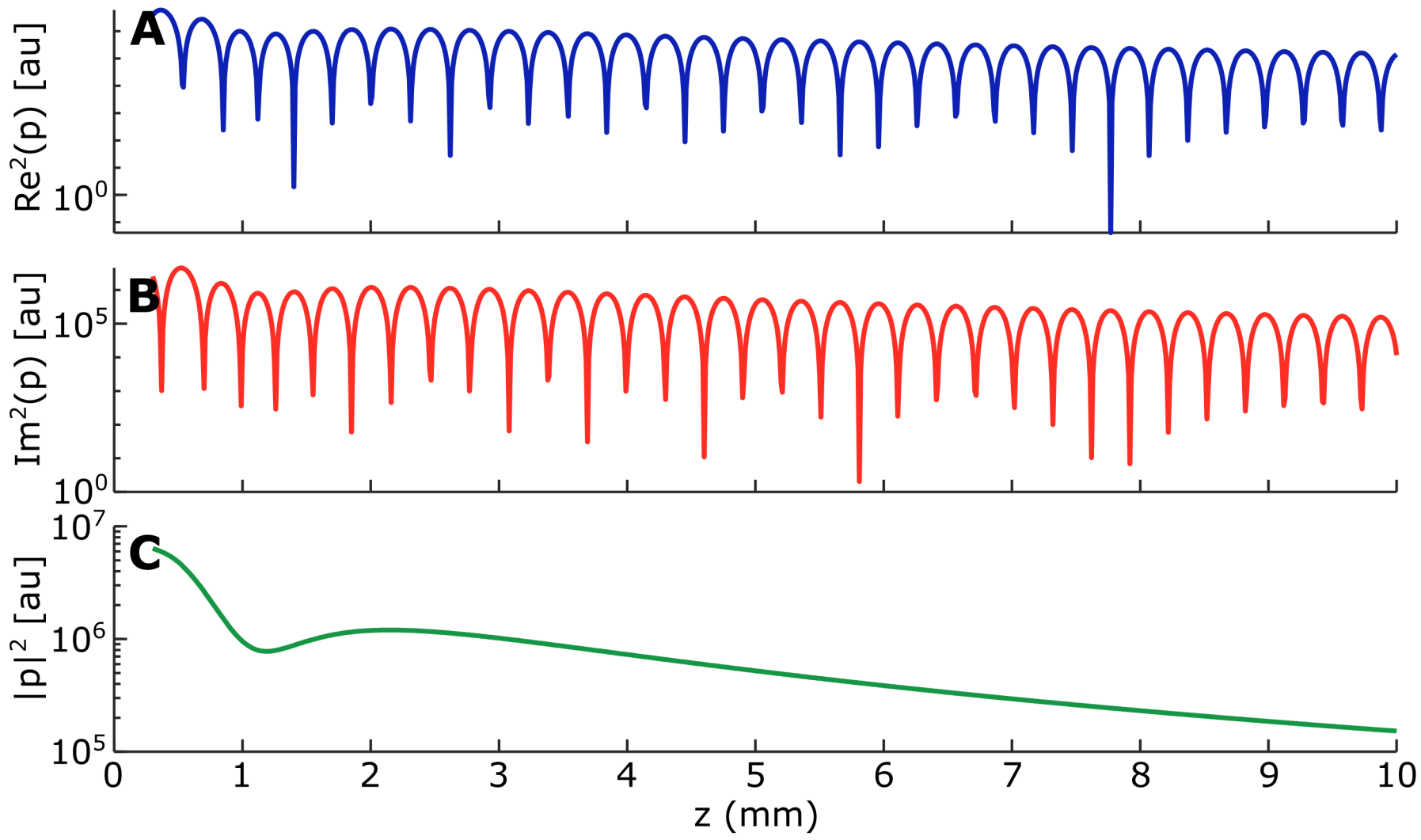

Figure S28: **Squared pressure visualized on a log scale for one therapy array. (A)** Real component, **(B)** imaginary component, and **(C)** the magnitude of the squared pressure emitted from the one therapeutic array on-axis, displayed on a log scale.

12.1.6 Transition from near-field to far-field regions

The distance at which the transition from near-field to the far-field is given by Equation S9:

$$z_{nf} = \frac{D^2}{4\lambda}, \tag{S9}$$

where D is the effective aperture diameter, and λ is the wavelength.

The effective aperture diameter (i.e., array width) is given by Equation S10:

$$\begin{aligned} D &= N_t \cdot pitch - kerf \\ &= 8 * (300\mu m) - 100\mu m \\ &= 2.3mm \end{aligned} \tag{S10}$$

The wavelength is given by Equation S11:

$$\begin{aligned} \lambda &= \frac{c}{f_t} \\ &= \frac{1500cm/s}{2.5 \cdot 10^6 1/s} \\ &= 0.6mm \end{aligned} \tag{S11}$$

Thus, $z_{nf} = 2.2$ mm, and the theoretical pressure at the near-field, far-field transition depth is 1096.1 au. This near-field, far-field transition depth, also known as the Rayleigh distance, corresponds with the local pressure maximum. In the far-field (i.e., the depths greater than the Rayleigh distance), there is more consistent, predictable wave propagation mimicking a plane wave.

To represent the pressure output in units of MPa, the material properties would need to be incorporated into the surface velocity measurement. To experimentally measure the true surface velocity, laser Doppler vibrometry could be utilized (*14*).

12.2 Off-axis, 1-dimensional pressure profile for dual, 8-element therapeutic arrays separated by 1 cm

The previous equations present the on-axis pressure of a single therapeutic ultrasound array from MUSIC. To incorporate the two therapeutic arrays, the 1-cm separation between the arrays (S_t, Figure S25B) is introduced to the pressure calculation formulas.

12.2.1 Off-axis pressure calculation

The first array is centered at $x_1 = -S_t/2 = -0.5$ cm, and the second array is centered at $x_2 = +S_t/2 = +0.5$ cm in the $y = 0$ plane. For the two arrays, the total pressure at any point $\mathbf{r}$ will be the sum of the pressures generated by each array.

We first evaluated the off-axis pressure at $x = 0$ (in between the arrays). Thus, the total pressure, p_{total} is given by Equation S12:

$$p_{total}(z) = p_1(z) + p_2(z). \tag{S12}$$

To incorporate the x-offset of each array, the center location of each array is included in Equation S6 to become Equation S13:

$$|\mathbf{r} - \mathbf{r}_n| = \sqrt{\left(x - x_n \pm \frac{S_t}{2}\right)^2 + (y - y_n)^2 + (z - z_n)^2}$$

$$= \sqrt{\left(x_n \pm \frac{S_t}{2}\right)^2 + z^2}. \tag{S13}$$

Thus, Equation S12 is expanded as follows:

$$p_{total}(z) = j\rho f_t \sum_{n=1}^{N_t} \frac{v_0 e^{-jk_t\sqrt{\left(x_n+\frac{S_t}{2}\right)^2+z^2}}}{\sqrt{\left(x_n+\frac{S_t}{2}\right)^2+z^2}} \Delta x_n + j\rho f_t \sum_{n=1}^{N_t} \frac{v_0 e^{-jk_t\sqrt{\left(x_n-\frac{S_t}{2}\right)^2+z^2}}}{\sqrt{\left(x_n-\frac{S_t}{2}\right)^2+z^2}} \Delta x_n. \tag{S14}$$

The 1D, off-axis acoustic pressure output of the dual therapeutic arrays is shown in Figure S29-Figure S31.

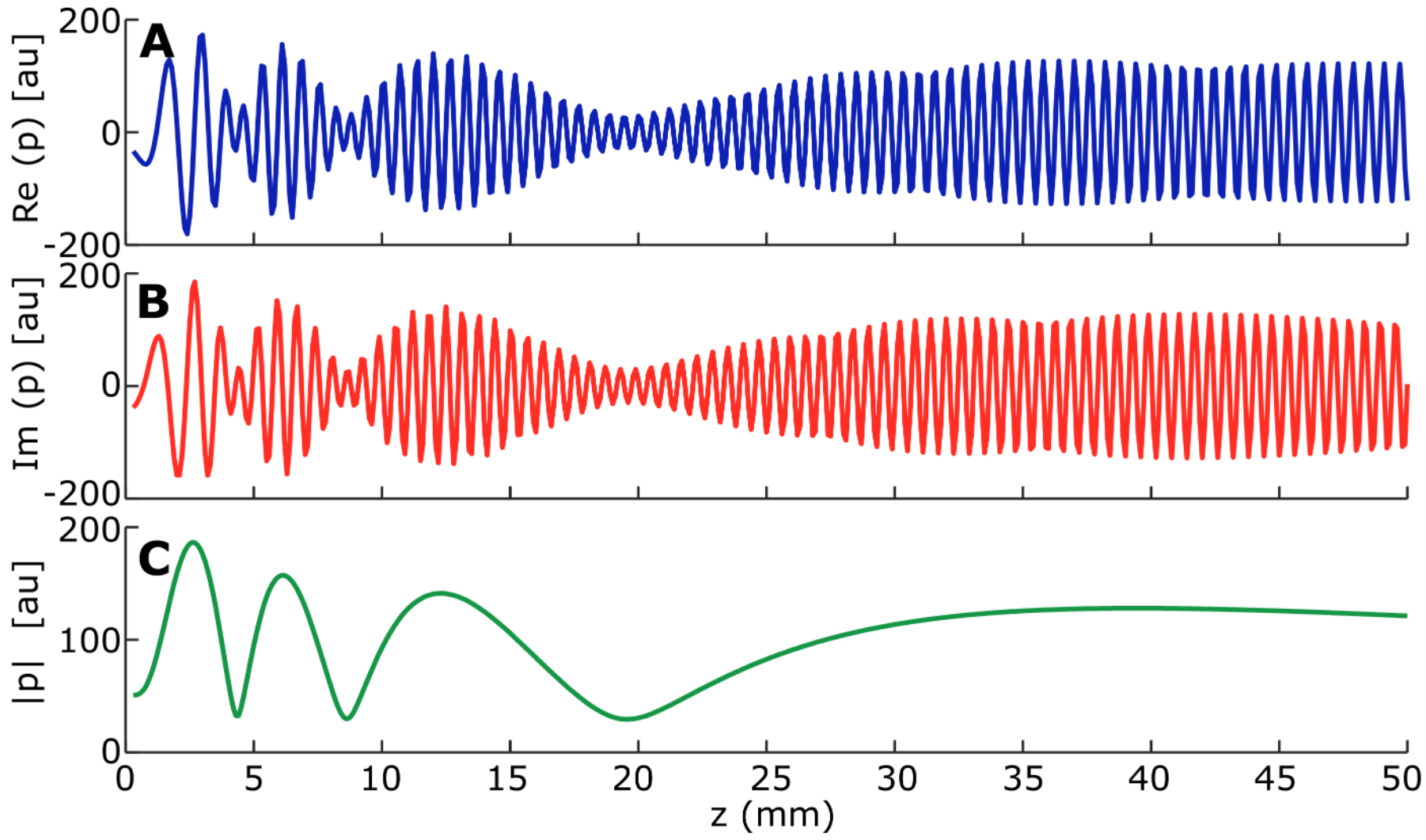

Figure S29: **Off-axis pressure profile for both therapy arrays. (A)** Real component, **(B)** imaginary component, and **(C)** the magnitude of the pressure emitted from the two therapeutic arrays in between the arrays (i.e., $x = 0$).

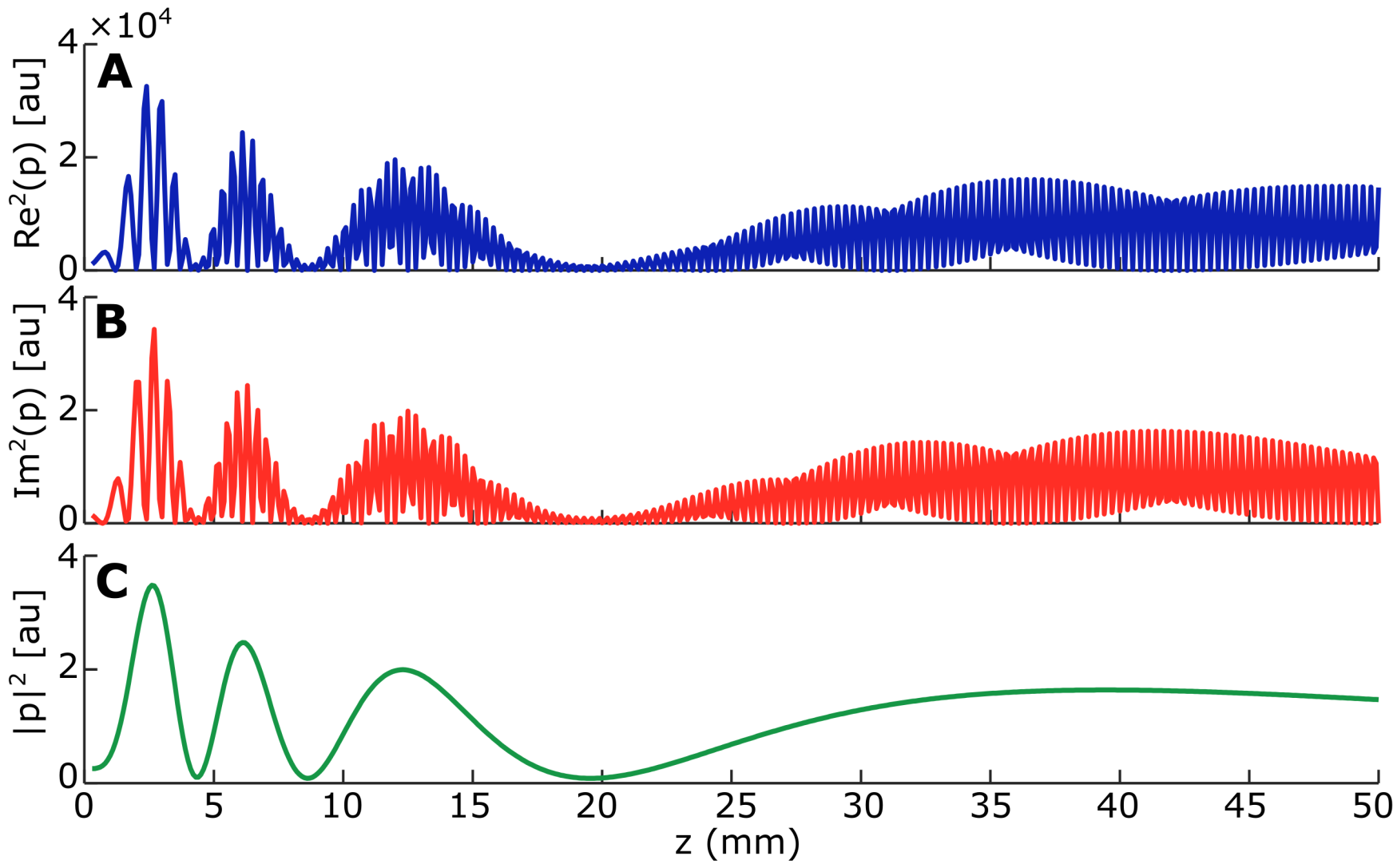

Figure S30: **Off-axis squared pressure profile for both therapy arrays.** **(A)** Real component, **(B)** imaginary component, and **(C)** the magnitude of the squared pressure emitted from the two therapeutic arrays in between the arrays (i.e., $x = 0$).

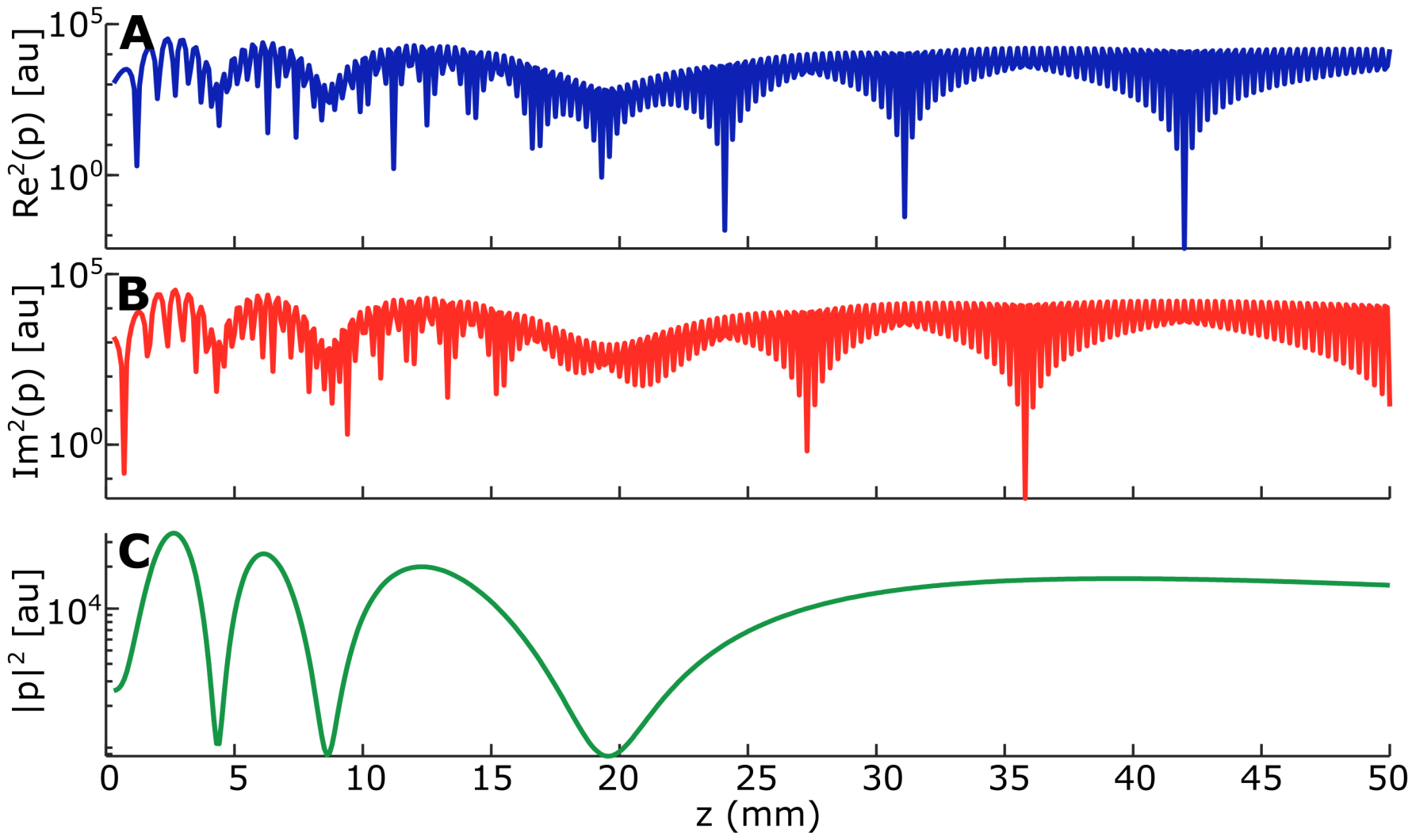

Figure S31: **Off-axis squared pressure profile visualized on a log scale for both therapy arrays.** **(A)** Real component, **(B)** imaginary component, and **(C)** the magnitude of the squared pressure emitted from the two therapeutic arrays in between the arrays (i.e., $x = 0$), displayed on a log scale.

12.2.2 Discussion of destructive interference effects in the dual therapeutic array configuration

At approximately 4.4 mm, 8.5 mm, and 19.6 mm depths, we observed local pressure minima between the sound waves emitted by the two therapeutic arrays. These regions represent locations where the acoustic pressure wave from each array is out of phase, resulting in pressure reduction. This phase mismatch is a direct result of the configuration of the two arrays, causing destructive interference along the off-axis $x = y = 0$ line.

12.3 2D pressure field analysis for the therapeutic arrays

After assessing the theoretical behavior along the $x = y = 0$ of the therapeutic arrays, we calculated the 2D pressure field. We assume an infinite y-axis boundary of the therapeutic arrays, so $(y - y_n) = 0$. Thus, the 2D pressure field in the XZ plane is calculated according to Equation S15 and illustrated in Figure S32. Figure S32A shows the 2D pressure field from a single therapeutic array, while Figure S32B shows the interaction of the two arrays. Notably, the destructive interference discussed in 12.2.2 is observed with the pressure minima at $z = 4.4$ mm and $z = 8.5$ mm along $x = 0$. $x = 0$ indicates the site of injury for MUSIC. Therefore, these pressure fields indicate that MUSIC does not maximize pressure over this injury site. Rather, the two therapeutic arrays target regions of the spinal cord a few millimeters away from the epicenter of injury (umbra). This region located neighboring the umbra is referred to as the penumbral zone (*50*). Targeting the penumbral zone with therapeutic ultrasound is critical because this region is prone to secondary injuries (e.g., ischemia, oxidative stress, loss of autoregulation, inflammation, and excitotoxicity (*3*)) following the initial trauma. These pressure fields could be utilized in further studies to optimize therapeutic interventions to limit the progression of secondary injury.

$$
\begin{aligned}
p_{total}(x, y, z) = j\rho f_t \sum_{n=1}^{N_t} \frac{v_0 e^{-jk_t \sqrt{\left(x - x_n + \frac{S_t}{2}\right)^2 + z^2}}}{\sqrt{\left(x - x_n + \frac{S_t}{2}\right)^2 + z^2}} \Delta x_n \\
+ j\rho f_t \sum_{n=1}^{N_t} \frac{v_0 e^{-jk_t \sqrt{\left(x - x_n - \frac{S_t}{2}\right)^2 + z^2}}}{\sqrt{\left(x - x_n - \frac{S_t}{2}\right)^2 + z^2}} \Delta x_n.
\end{aligned} \tag{S15}
$$

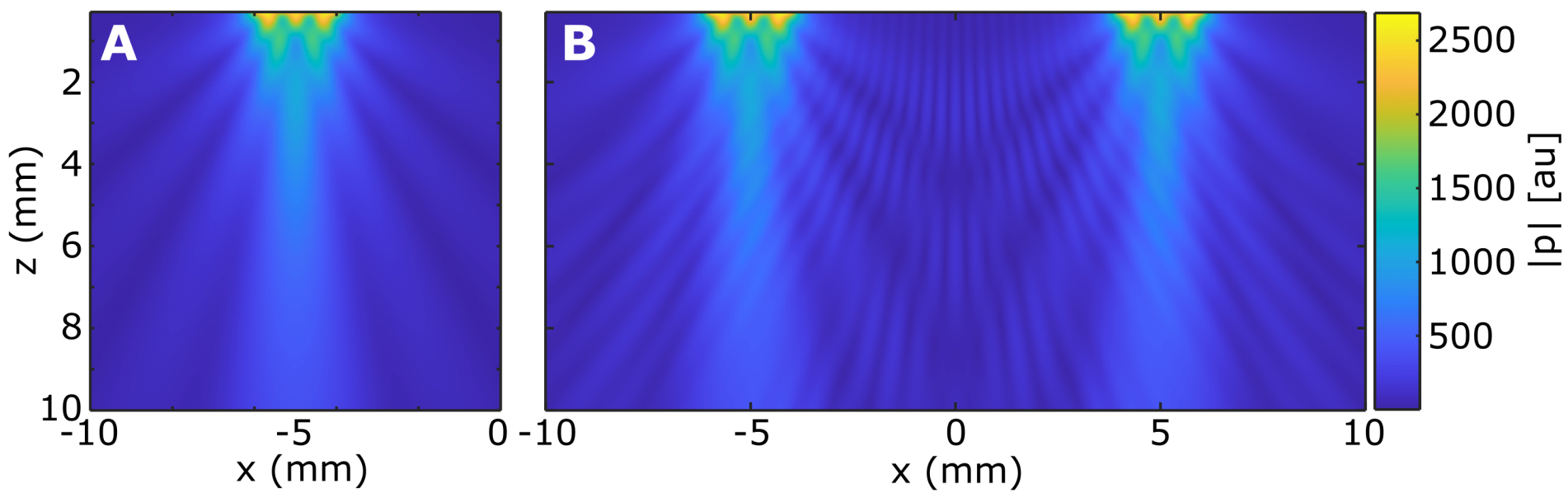

Figure S32: **XZ theoretical pressure maps** emitted from **(A)** one and **(B)** two therapeutic arrays at $y = 0$.

12.4 On-axis, 1-dimensional pressure profile for one, 64-element imaging array

12.4.1 Geometry and boundary conditions

The imaging arrays are oriented along the x-axis, with the elements arranged along the y-axis (Figure S25B).

The following specifications are provided for a single imaging array:

- Element pitch: 135 μm
- Element width, Δy_n: 111 μm
- Element kerf: 24 μm
- Frequency, f_i: 10.5 MHz
- Number of elements, N_i: 64

12.4.2 On-axis pressure calculation

When the field position is on-axis, directly in front of the center of the array, and given that the individual elements are oriented along y, $|\mathbf{r} - \mathbf{r}_n|$ is simplified as follows:

$$|\mathbf{r} - \mathbf{r}_n| = \sqrt{y_n^2 + z^2} \tag{S16}$$

Thus, the on-axis, 1D pressure for a single imaging array is given by Equation S17:

$$p(z) = j\rho f_i \sum_{n=1}^{N_i} \frac{v_0 e^{-jk_i\sqrt{y_n^2+z^2}}}{\sqrt{y_n^2 + z^2}} \Delta y_n, \tag{S17}$$

where y_n is the center position of each element and Δy_n is the width of each element.

Assuming the elements are symmetric about $y = 0$, the center position of each element is given by Equation S18:

$$y_n = \left(n - \frac{N_i - 1}{2}\right) * pitch, \tag{S18}$$

where n ranges from 0 to (N_i-1) (i.e., the index of the array element). Therefore, $y_n = [-4.25, -4.18, ..., 4.18, 4.25]$ mm.

The 1D theoretical, on-axis pressure of a single imaging array is shown in Figure S33-Figure S35.

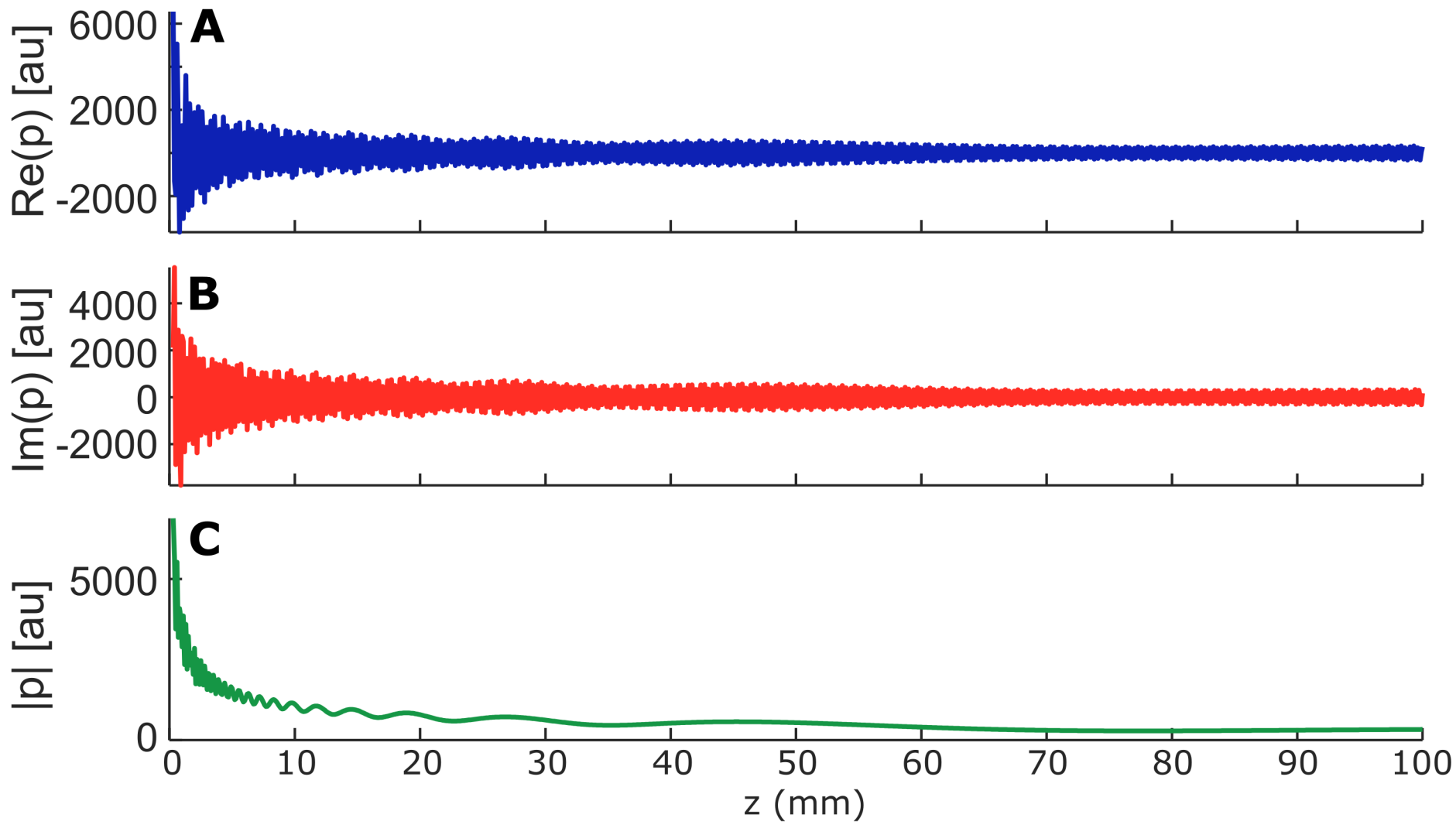

Figure S33: **On-axis pressure versus distance for an imaging array.** **(A)** Real component, **(B)** imaginary component, and **(C)** the magnitude of the on-axis pressure emitted from a single imaging array.

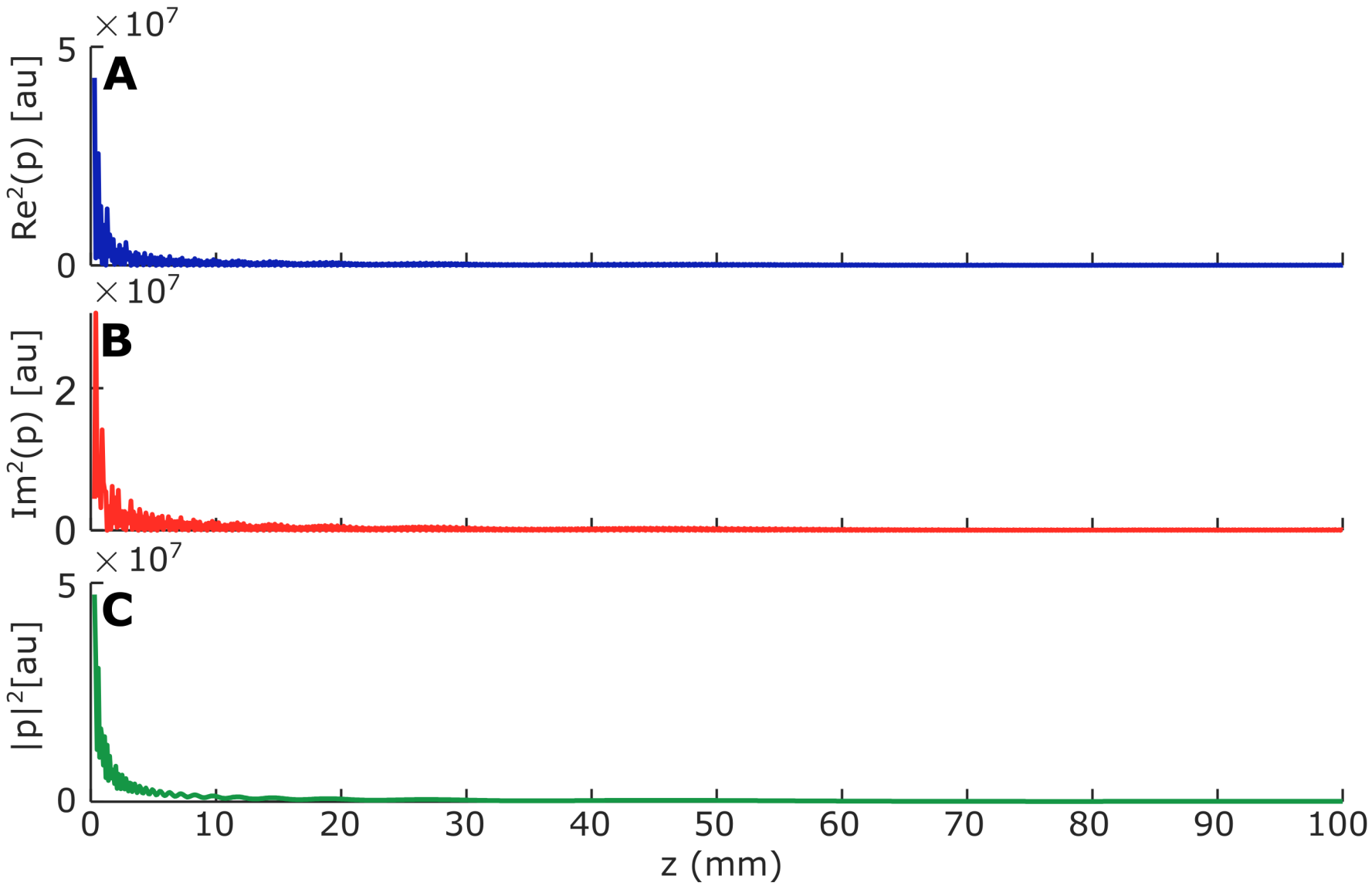

Figure S34: **On-axis, squared pressure versus distance for an imaging array. (A)** Real component, **(B)** imaginary component, and **(C)** the magnitude of the squared, on-axis pressure emitted from a single imaging array.

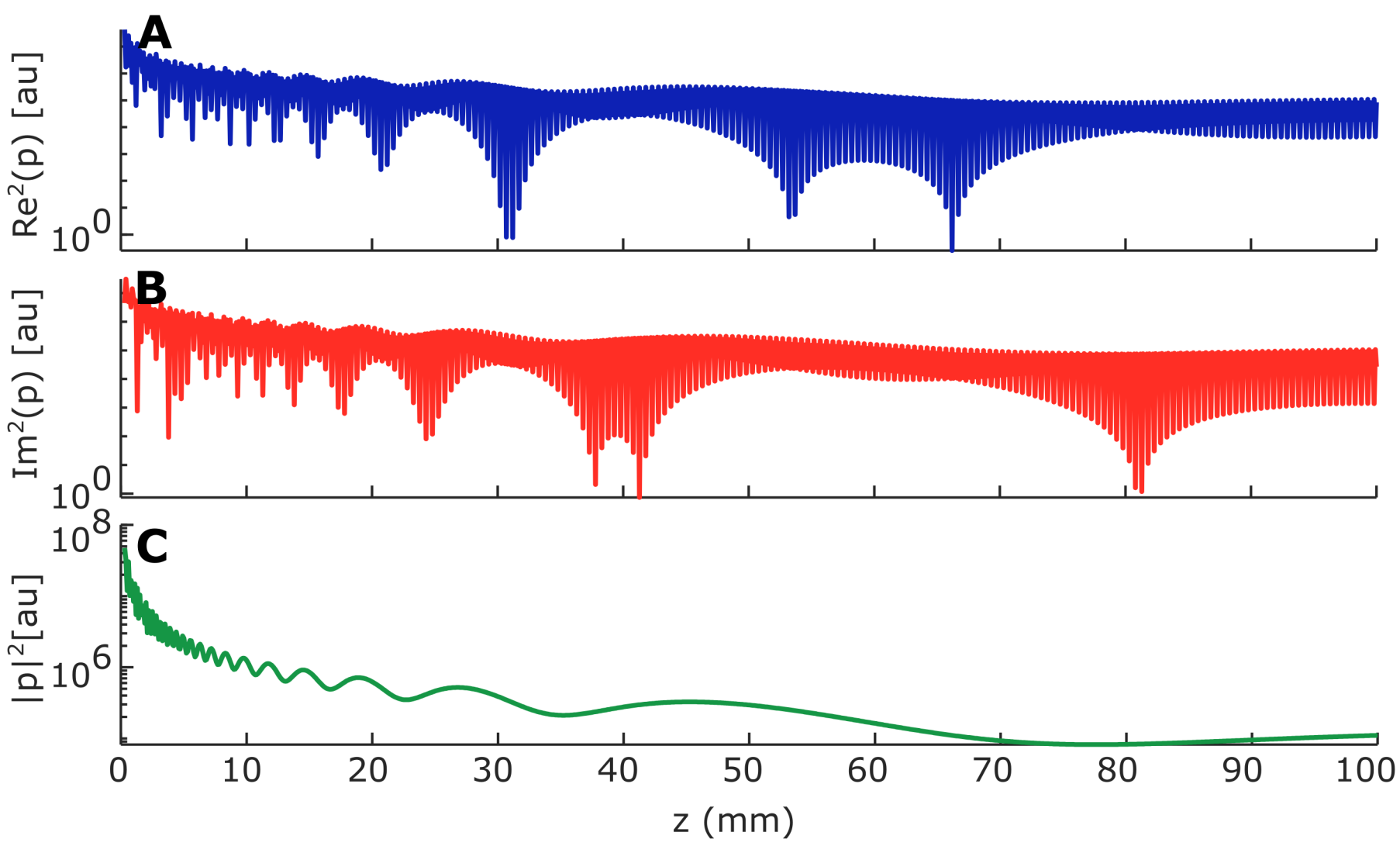

Figure S35: **On-axis, squared pressure versus distance for an imaging array on a log scale.** **(A)** Real component, **(B)** imaginary component, and **(C)** the magnitude of the squared, on-axis pressure emitted from a single imaging array displayed on a log scale.

The Rayleigh distance (Equation S9) was calculated to be 129.9 mm. Because this distance is beyond the size and depth of the spinal cord, we incorporated an acoustic lens into our design to bring forward the focus depth to that of the spinal cord.

12.5 Off-axis, 1-dimensional pressure profile for dual, 64-element imaging arrays separated by 3.4 mm

12.5.1 Off-axis pressure calculation

The two imaging arrays are centered at $\pm S_i/2 = \pm 1.7$ mm. Given this x offset, the distance from the field position to the source position is given by Equation S19:

$$|\mathbf{r} - \mathbf{r}_n| = \sqrt{\left(x \pm \frac{S_i}{2}\right)^2 + (y - y_n)^2 + z^2}. \tag{S19}$$

When measuring the off-axis profile in between the two imaging arrays (i.e., $x = y = 0$), the pressure outputs of the two arrays are symmetric and equivalent. Thus, Equation S12 is simplified to

$$p_{total}(z) = 2p_1(z) \tag{S20}$$

$$= 2j\rho f_i \sum_{n=1}^{N_i} \frac{v_0 e^{-jk_i\sqrt{\left(\frac{S_i}{2}\right)^2 + y_n^2 + z^2}}}{\sqrt{\left(\frac{S_i}{2}\right)^2 + y_n^2 + z^2}} \Delta y_n. \tag{S21}$$

The 1D theoretical pressure output of the two adjacent imaging arrays is shown in Figure S36-Figure S38.

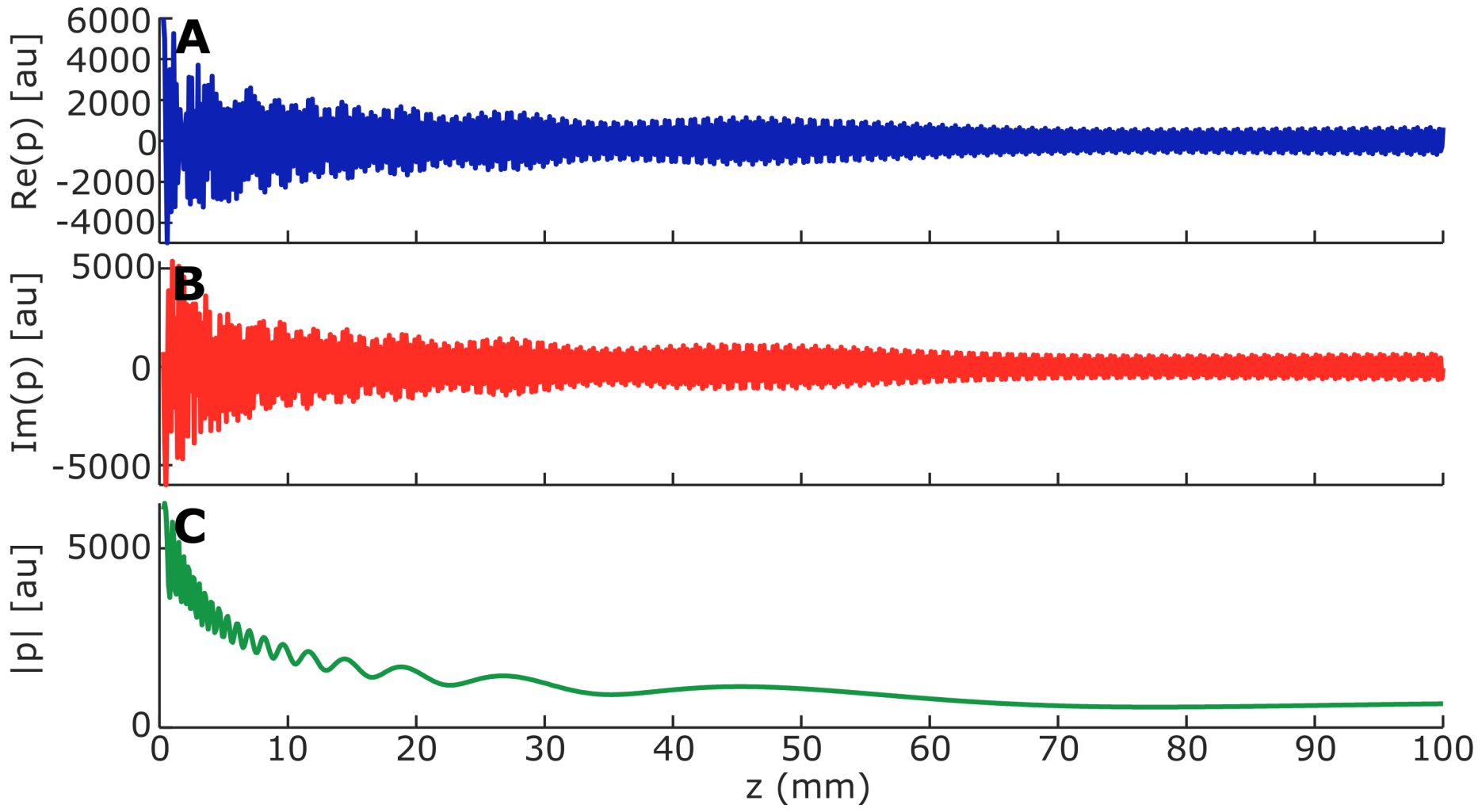

Figure S36: **Off-axis, pressure versus distance for both imaging arrays. (A)** Real component, **(B)** imaginary component, and **(C)** the magnitude of the pressure emitted from the two adjacent imaging arrays in between the arrays (i.e., $x = 0$, $y = 0$).

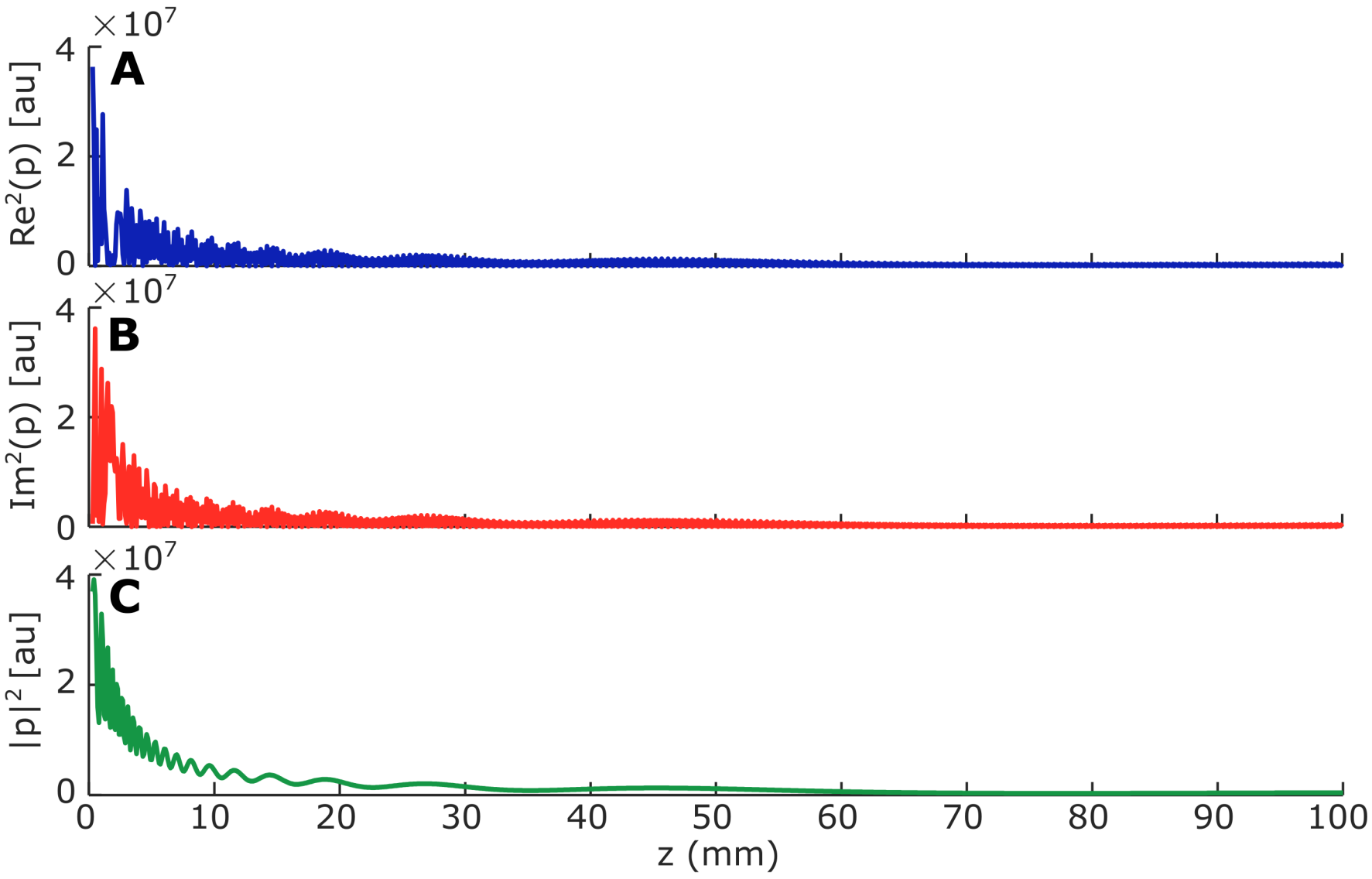

Figure S37: **Off-axis, squared pressure versus distance for both imaging arrays. (A)** Real component, **(B)** imaginary component, and **(C)** the magnitude of the squared pressure emitted from the two adjacent imaging arrays in between the arrays (i.e., $x = 0$, $y = 0$).

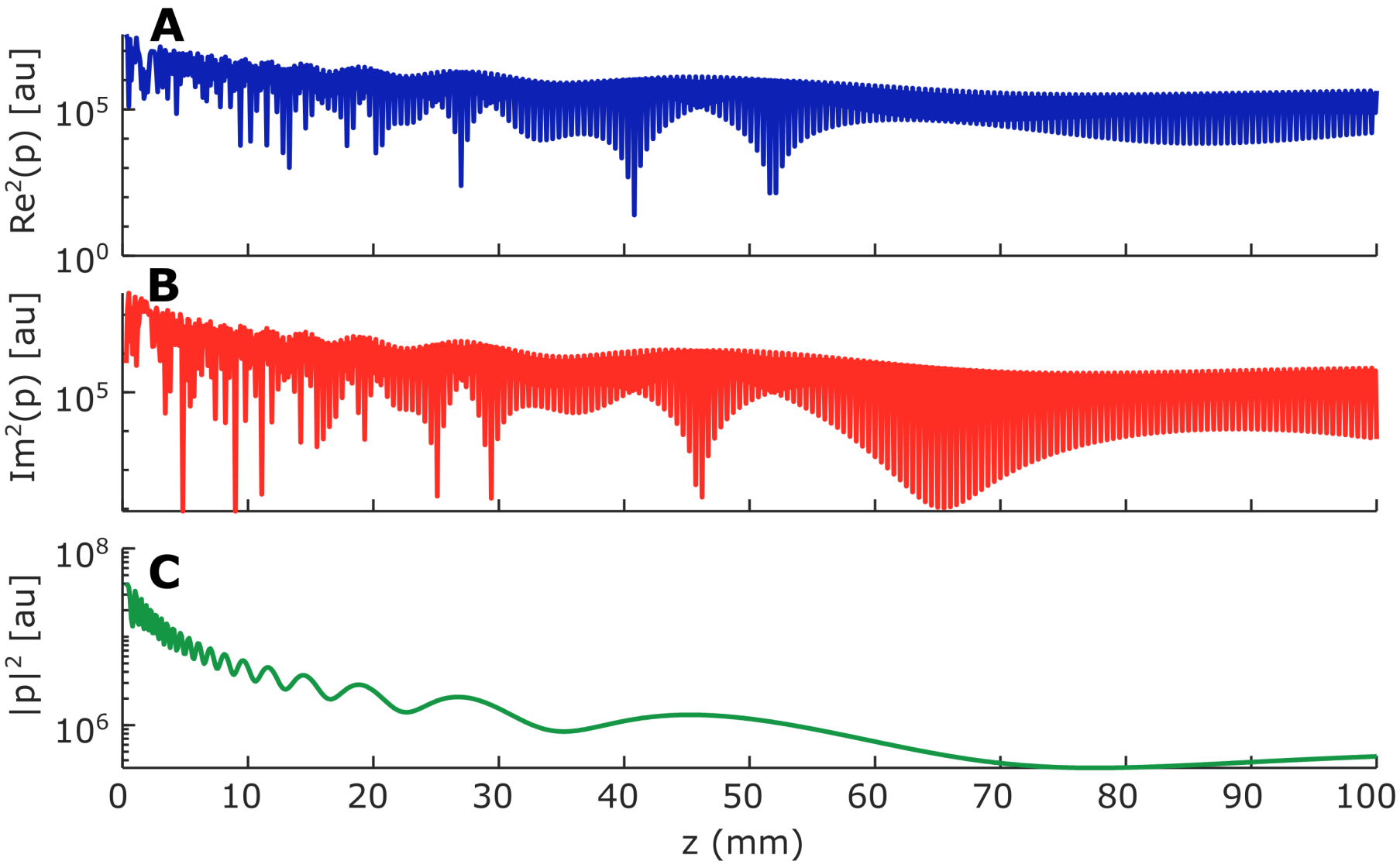

Figure S38: **Off-axis, squared pressure versus distance for both imaging arrays on a log scale.** **(A)** Real component, **(B)** imaginary component, and **(C)** the magnitude of the squared pressure emitted from the two adjacent imaging arrays in between the arrays (i.e., $x = 0$, $y = 0$) displayed on a log scale.

12.6 2D pressure field analysis for the imaging arrays

Expanding Equation S21 to visualize the theoretical pressure field in the XZ and YZ planes from the imaging arrays, we produce Equation S22.

$$p_{total}(x,y,z) = j\rho f_i \sum_{n=1}^{N_i} \left(\frac{v_0 e^{-jk_i \sqrt{\left(x+\frac{S_i}{2}\right)^2 + (y-y_n)^2 + z^2}}}{\sqrt{\left(x+\frac{S_i}{2}\right)^2 + (y-y_n)^2 + z^2}} \right) \Delta y_n + j\rho f_i \sum_{n=1}^{N_i} \left(\frac{v_0 e^{-jk_i \sqrt{\left(x-\frac{S_i}{2}\right)^2 + (y-y_n)^2 + z^2}}}{\sqrt{\left(x-\frac{S_i}{2}\right)^2 + (y-y_n)^2 + z^2}} \right) \Delta y_n. \tag{S22}$$

The YZ plane under a single imaging array is given by Figure S39A, while the XZ plane of the pressure emitted by the dual imaging arrays is given by Figure S39B at $y = 0$ mm.

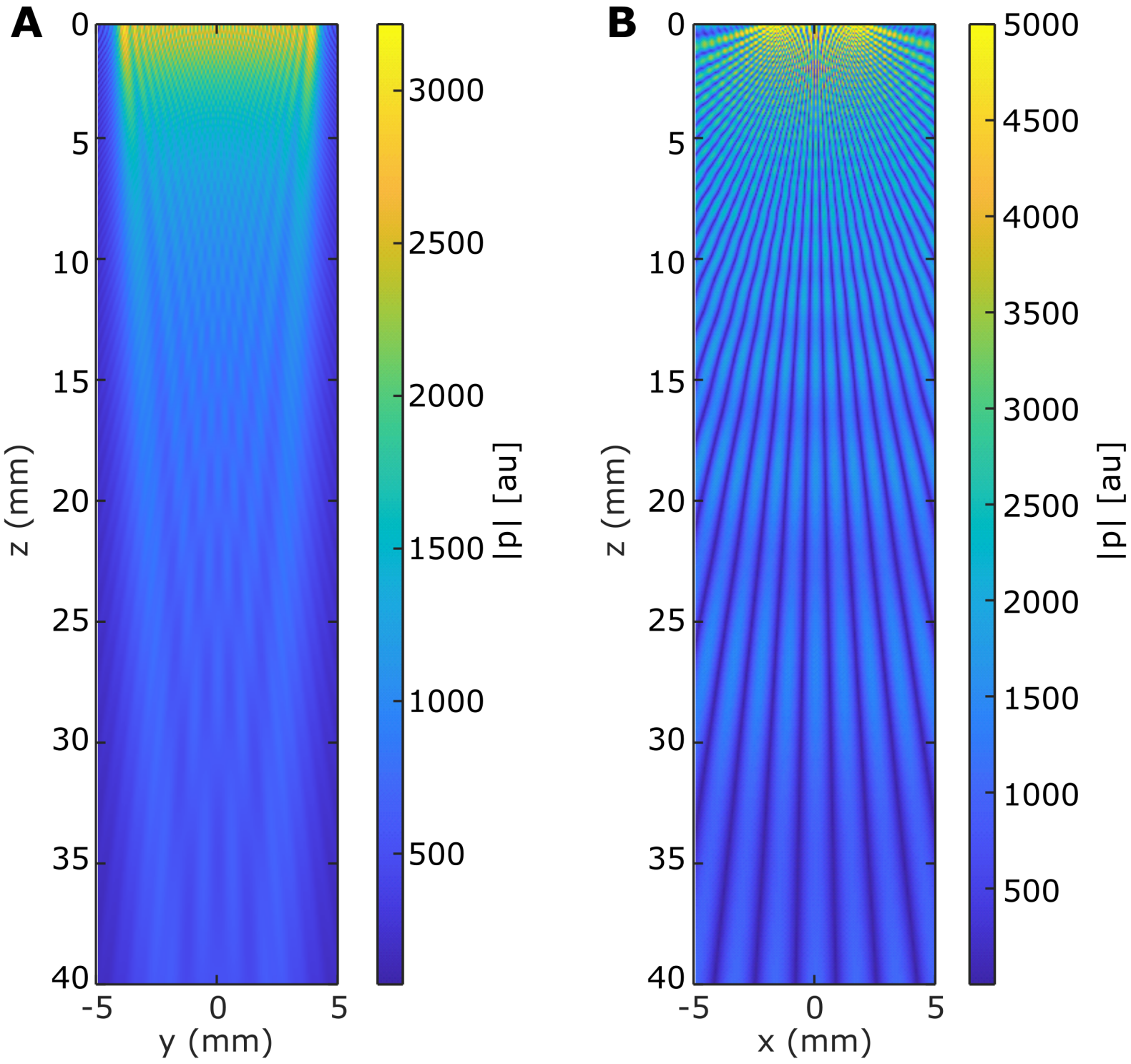

Figure S39: **Theoretical pressure field for the imaging arrays. (A)** YZ **(B)** XZ theoretical pressure map emitted from a single and both adjacent imaging arrays, respectively. The YZ plane is shown directly under a single imaging array ($x = 1.7$ mm), while the XZ plane is shown at $y = 0$ mm.

12.7 Generalized model for imaging & therapeutic arrays

We generalize Equation S22 and Equation S15 into a single, unified formula by setting N_m to be the number of elements in array m, $\mathbf{r}_{m,n}$ and the coordinates of the nth element in the mth array, respectively, and $S_{m,x}$ and $S_{m,y}$ be the offset of array m from $x = 0$ and $y = 0$, respectively. This generalized acoustic pressure equation is given by Equation S23:

$$p(x, y, z) = j\rho f_1 \sum_{n=1}^{N_1} \frac{v_0 e^{-jk_1|\mathbf{r}-\mathbf{r}_{1,n}|}}{|\mathbf{r}-\mathbf{r}_{1,n}|}\Delta n_1 + j\rho f_2 \sum_{n=1}^{N_2} \frac{v_0 e^{-jk_2|\mathbf{r}-\mathbf{r}_{2,n}|}}{|\mathbf{r}-\mathbf{r}_{2,n}|}\Delta n_2 + \ldots + j\rho f_m \sum_{n=1}^{N_m} \frac{v_0 e^{-jk_m|\mathbf{r}-\mathbf{r}_{m,n}|}}{|\mathbf{r}-\mathbf{r}_{m,n}|}\Delta n_m, \tag{S23}$$

and the general source point location and distance from the field point to the source point are given by Equation S24 and Equation S25, respectively:

$$\mathbf{r}_{m,n} = (x_n + S_{m,x}, y_n + S_{m,y}, 0), \tag{S24}$$

$$|\mathbf{r}-\mathbf{r}_{m,n}| = \sqrt{(x - x_n - S_{m,x})^2 + (y - y_n - S_{m,y})^2 + z^2}, \tag{S25}$$

where arrays with elements oriented along the x axis have $y_n = 0$, and arrays with elements oriented along the y axis have $x_n = 0$.

In the case of MUSIC, the combined pressure is given by:

$$\begin{aligned} p_{music}(x, y, z) = &\, j\rho f_t \sum_{n=1}^{N_t} \frac{v_0 e^{-jk_t\sqrt{(x-x_n+\frac{S_t}{2})^2+y^2+z^2}}}{\sqrt{(x-x_n+\frac{S_t}{2})^2+y^2+z^2}}\Delta x_n \\ &+ j\rho f_t \sum_{n=1}^{N_t} \frac{v_0 e^{-jk_2\sqrt{(x-x_n-\frac{S_t}{2})^2+y^2+z^2}}}{\sqrt{(x-x_n-\frac{S_t}{2})^2+y^2+z^2}}\Delta x_n \\ &+ j\rho f_i \sum_{n=1}^{N_i} \frac{v_0 e^{-jk_i\sqrt{(x+\frac{S_i}{2})^2+(y-y_n)^2+z^2}}}{\sqrt{(x+\frac{S_i}{2})^2+(y-y_n)^2+z^2}}\Delta y_n \\ &+ j\rho f_i \sum_{n=1}^{N_i} \frac{v_0 e^{-jk_i\sqrt{(x-\frac{S_i}{2})^2+(y-y_n)^2+z^2}}}{\sqrt{(x-\frac{S_i}{2})^2+(y-y_n)^2+z^2}}\Delta y_n \end{aligned} \tag{S26}$$

In scenarios such as MUSIC where the individual arrays can be controlled independently, the user may choose to operate some or all of the arrays simultaneously. To account for the choice of array activation, we introduce an activation coefficient, α, which is 1 when active and 0 when inactive.

$$\begin{aligned} p_{music}(x, y, z) = &\, \alpha_{therapy,1}\left(j\rho f_t \sum_{n=1}^{N_t} \frac{v_0 e^{-jk_t\sqrt{(x-x_n+\frac{S_t}{2})^2+y^2+z^2}}}{\sqrt{(x-x_n+\frac{S_t}{2})^2+y^2+z^2}}\Delta x_n \right) \\ &+ \alpha_{therapy,2}\left(j\rho f_t \sum_{n=1}^{N_t} \frac{v_0 e^{-jk_t\sqrt{(x-x_n-\frac{S_t}{2})^2+y^2+z^2}}}{\sqrt{(x-x_n-\frac{S_t}{2})^2+y^2+z^2}}\Delta x_n \right) \end{aligned}$$

$$+ \alpha_{imaging,1}\left(j\rho f_i \sum_{n=1}^{N_i} \frac{v_0 e^{-jk_i\sqrt{(x+\frac{S_i}{2})^2+(y-y_n)^2+z^2}}}{\sqrt{(x+\frac{S_i}{2})^2+(y-y_n)^2+z^2}}\Delta y_n\right)$$

$$+ \alpha_{imaging,2}\left(j\rho f_i \sum_{n=1}^{N_i} \frac{v_0 e^{-jk_i\sqrt{(x-\frac{S_i}{2})^2+(y-y_n)^2+z^2}}}{\sqrt{(x-\frac{S_i}{2})^2+(y-y_n)^2+z^2}}\Delta y_n\right) \quad \text{(S27)}$$

If both imaging arrays are active ($\alpha_{imaging,1} = \alpha_{imaging,2} = 1$) and both therapeutic arrays are active ($\alpha_{therapy,1} = \alpha_{therapy,2} = 1$), the combined pressure is shown in Figure S40.

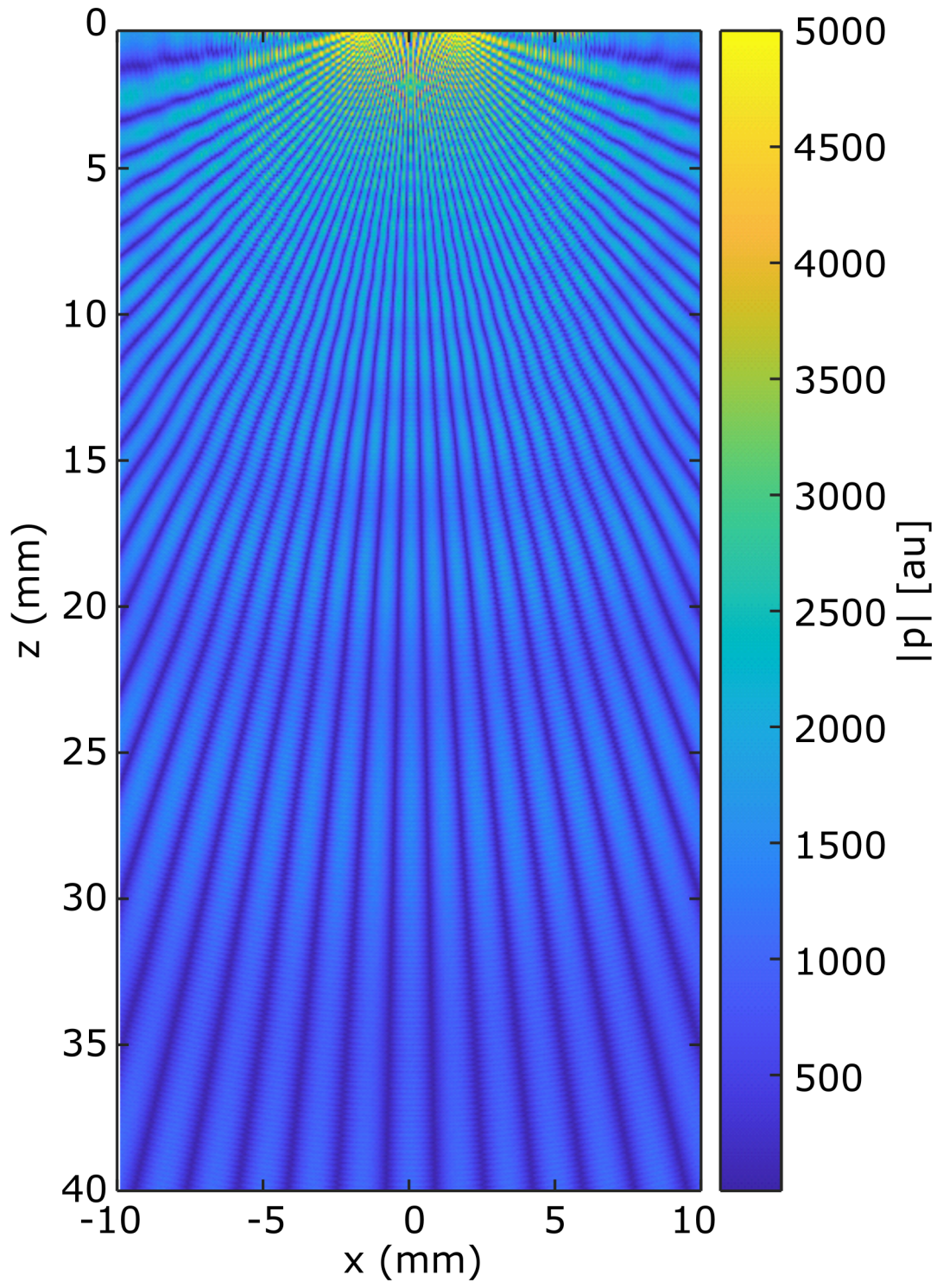

Figure S40: **XZ theoretical pressure map** emitted from both adjacent imaging arrays and both therapeutic arrays. The XZ plane is shown at $y = 0$ mm.

12.8 Conclusion

Derived from the Rayleigh-Sommerfeld diffraction integral, we present a generalized acoustic pressure field formula for a device composed of multiple 1-dimensional linear ultrasound arrays, in addition to a specific MUSIC formula. We provide detailed formulas for both on-axis and off-axis pressure fields, whether individual arrays are activated individually or simultaneously. Equation S23 models the acoustic pressure field produced by parallel or perpendicular arrays for diagnostic and therapeutic ultrasound applications. Specifically for MUSIC, Equation S27 represents both 64-element imaging arrays oriented along the y-axis and 8-element therapeutic arrays oriented along the x-axis. The MUSIC formula can aid in optimizing clinical outcomes and imaging quality after spinal cord injury. The generalized pressure formula represents the near-field and far-field patterns. Furthermore, Equation S23 can accommodate a wide variety of device and array configurations, enhancing versatility of the Rayleigh integral. To the best of our knowledge, this theoretical formula is the first to model the acoustic pressure of multiple 1-dimensional linear arrays. This model promotes novel clinical applications utilizing image-guided and therapeutic ultrasound.

Supplementary Section 13
In vivo porcine deployment of MUSIC

All animal studies, including terminal and survival studies, were conducted using porcine models following procedures approved by the Johns Hopkins Animal Care and Use Committee. A total of N = 17 female Yorkshire pigs weighing 35-40 kg were tested. All animals were provided a 7-day acclimation period and a 12-hour fasting period prior to the scheduled procedure.

On the day of surgery, pigs were sedated with an intramuscular injection of ketamine (20-30 mg/kg) and xylazine (2-3 mg/kg), followed by intubation using intravenous propofol (0.83-1.66 mg/kg) and maintenance on isoflurane (0.5-2.5% + 1-2 L O2/min) and fentanyl constant-rate infusion (0.03-0.2 mg/kg/hr). Endotracheal intubation was confirmed via auscultation and X-ray imaging. The spinous process of T5 was marked on the skin during X-ray imaging. After pigs were transferred to the operating table, a femoral arterial line was placed under ultrasound guidance (for non-survival studies). The pigs were then flipped into a prone position and the surgical area was sterilized with betadine. Heart rate, blood pressure, respiratory rate, and rectal temperature were monitored continuously and recorded every 15–30 minutes. Hypothermia was prevented with warming blankets.

A midline incision was performed from T4–T6 using T5 as a reference landmark and palpating for the adjacent spinous processes. This was followed by subcutaneous, soft-tissue, and muscle dissection using monopolar electrocautery. After exposing the spinous processes, a sub-periosteal dissection was performed down to the lamina over the target vertebra using a combination of monopolar electrocautery and a Cobb periosteal elevator. Using a combination of Kerrison and Leksell rongeurs, the interspinous ligaments, spinous processes, and lamina were removed until the ligamentum flavum was visualized. Lamina was removed laterally to the level of the facet joints to create a large window with ample space for the placement of MUSIC. Dissection was continued laterally until transverse processes were visible. The cortical layer of the right T4, left T5, and right T6 transverse processes were removed, and a pedicle finder was advanced 2 cm anteromedially through the pedicle to create a pilot hole. A ball-tip probe was used to confirm there was no cortical breach, and Quartex titanium screws (Globus Medical, Audubon, PA, USA) were placed using these trajectories. Titanium rods were placed on the screws and caps were used to fix the rods in place. Microdissection was used to incise and remove the ligamentum flavum. The epidural fat overlying the spinal cord was carefully suctioned. The custom rail system was connected to the spinal instrumentation and used to secure the MUSIC device over the spinal cord (**Supplementary Video 8**). The output flex cable of the MUSIC device was tunneled through the soft tissues and externalized through a separate exit site before wound closure, allowing the surgical field to be closed while maintaining external access to the device. The externalized segment was routed flat against the body to reduce exposed loops and provide practical strain relief during handling and repositioning. In a clinical workflow, this exit site would be managed with sterile occlusive dressing practices analogous to those used for temporary spinal or lumbar drainage systems. The i22LH8 transducer of the Canon Aplio i800 was also secured over the spinal cord via a custom probe holder with attachments to the spinal hardware, allowing it to serve as a clinical state-of-the-art comparison. Ultrasound images, including B-mode, PDI, and spectral Doppler data were collected throughout the surgery. For pigs who underwent SCI, the injury was induced by dropping a 20-g weight from a height of 15 cm or using a custom spinal cord impactor over T5 (*65*). At the termination of each study, pigs were euthanized with intravenous Euthasol (pentobarbital sodium and phenytoin sodium) solution. Euthanasia was confirmed by the veterinary team via auscultation and physical examination.

Supplementary Section 14
Explantation of MUSIC

A proof-of-concept explantation of MUSIC was performed to demonstrate a potential removal workflow without surgical intervention. Following device implantation, wound closure, and externalization of the flex cable through a tunneled exit site, the external cable was connected to a force sensor and progressively loaded in the caudal direction until device release and removal were achieved. The results support the feasibility of a future workflow in which MUSIC is removed through a tunneled lumbar exit site at the bedside without requiring reopening of the surgical field or return to the operating room. Future studies should evaluate this removal workflow across longer monitoring periods and additional clinical-use conditions.

Supplementary Section 15

Comparing MUSIC to state-of-the-art neural interfaces

Category	Metric	Elec. SCS (74–76)	Sonocloud-9 (77, 78)	ImPULS (30)	MUSIC
System Capabilities					
Platform Features	Chronicity	Chronic	Chronic	Research	**Acute/Sub-chr.**†
	On-device Monitoring	×	×	×	✓
	Application	Pain/BP	BBB Open.	Neuromod.	**Hemodyn.**
	Flexibility	Limited	Single	Single	**Multi-modal**
Acoustic & Stimulation Performance					
Targeting	Depth	–	64 mm	15 μm	**7 mm**
	Coverage	Non-spec.	40 mL	80×100 μm	**10×4 mm**
	Beam Steer.	No	No	No	**Electronic**
Stim Params	Peak Press.	–	0.2*	0.059	**0.4 MPa**
	Elements	16+ (Elec.)	9	1	**8 (Phased)**
	Focusing	Fixed	Fixed	Fixed	**Programmable**
Monitoring & Feedback					
Sensing	Real-time Feedback	Limited	×	×	✓ **(Doppler)**

* Pressure estimated from literature; † Chronic capability under development; N/R = Not reported.

Table S2: **Comprehensive comparison of implantable platforms.** MUSIC uniquely integrates therapeutic ultrasound deployment with real-time hemodynamic monitoring.

Supplementary Section 16
Respiratory monitoring capabilities

While monitoring the spinal cord remains the primary sensing objective of the MUSIC interface, it is important to recognize that systemic factors can significantly influence outcomes following SCI. Therefore, MUSIC is designed not only to capture blood flow data to extract cardiac metrics but also to track the subtle, natural movements of the spinal cord that occur during respiration.

This capability is critical because breathing function has been correlated with spinal cord perfusion pressure (*79*), and heart rate variability has been shown to differentiate between healthy and injured spinal cord states (*80*). To address these needs, MUSIC could incorporate algorithms that quantify both cardiac and respiratory function. By using ultrasound to track the spinal cord's pulsatile motion, MUSIC enables visualization of the cardiac and respiratory cycles, as illustrated in (Figure S41) (*81*).

To analyze these motions, MUSIC B-mode ultrasound images, acquired at a 60 Hz sampling frequency, were processed in Fiji using the TrackMate 7 feature (*82*, *83*), which tracks the movement of the central canal of the spinal cord. This analytical pipeline enables the use of pseudo-motion-mode (M-mode) ultrasound, demonstrating MUSIC's ability to provide an integrated monitoring platform that tracks vital signs in addition to spinal cord activity.

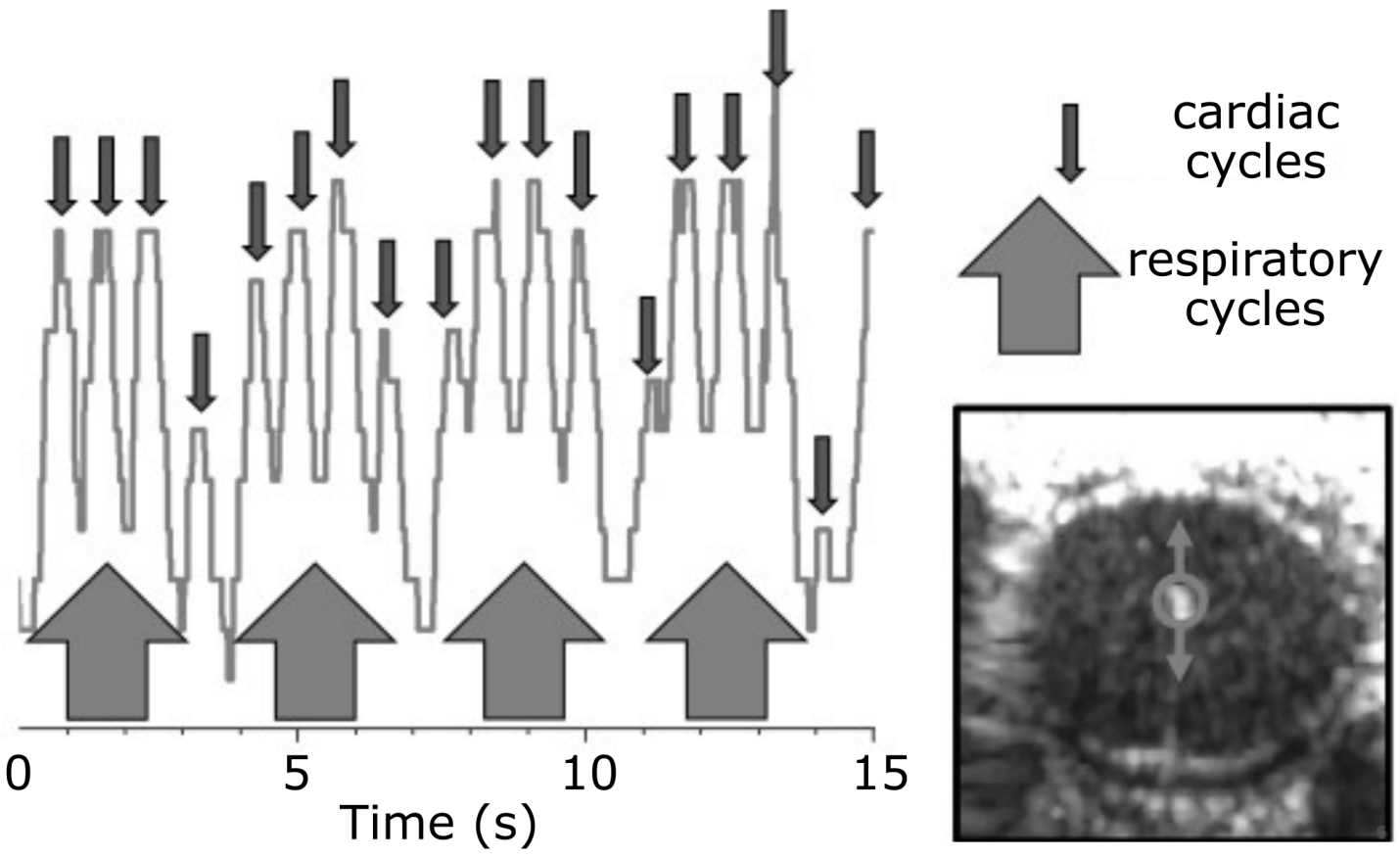

Figure S41: **MUSIC is capable of systemic physiological monitoring.** An motion-mode (M-mode)-like schematic illustrates how MUSIC could monitor respiratory and cardiac signals by tracking the subtle, natural movements of the spinal cord.

Supplementary Section 17
Acoustic and audio capabilities of MUSIC

Spinal cord injuries (SCI) are notoriously difficult to characterize using traditional imaging modalities such as B-mode ultrasound or power Doppler. This is especially complicated for slow flow (2-5 cm/s) in small microvasculature (e.g., sulcal artery: 140 microns) with high pulsatility. Consequently, accurate interpretation of this visual data requires highly trained neuroradiologists. However, such specialized personnel are neither accessible in all settings throughout the world, nor continuously on-call postoperatively for consecutive days even in the most resourced of settings. Despite the challenging diagnosis with visual insights, the corresponding audio outputs (healthy vs injured) have not been well studied and deserve a deeper investigation.

As a demonstration of clinical relevance for MUSIC's spectral Doppler capabilities, two audio files are included in this supplementary material (**Supplementary Videos 9-10**):

I) **Healthy Spinal Cord** – Spectral Doppler audio from a healthy porcine spinal cord (measured from the sulcal artery), demonstrating rhythmic and consistent flow patterns.

II) **Injured Spinal Cord** – Spectral Doppler audio from an injured porcine spinal cord (measured from the sulcal artery), demonstrating irregular and disrupted flow patterns characteristic of injury.

Using Equation S28 below, the blood flow velocities can be extracted from audio files.

$$v = \frac{c\Delta f}{2f_0 \cos\theta} \tag{S28}$$

where v is the blood velocity, Δf is the Doppler frequency shift, c is the speed of sound, f_0 is the center frequency of the ultrasound transducer, and θ is the angle between the transmitted ultrasound wave and the direction of blood flow.

The audio files produced by the MUSIC interface present an opportunity to study whether one can detect healthy vs perturbed flow based on audio alone. If so, this approach opens new avenues for:

1) AI-driven diagnostic tools, e.g. machine learning algorithms could analyze these sounds to identify specific patterns correlating with SCI severity 2) Traditional signal processing: Each signal can be used to conduct further in depth signal processing. For example, the envelope (which is a trace of the maximum blood flow velocity as a function of time) and its derivatives tend to be the main datastream for analysis. With few exceptions, including spectral broadening (data beneath the maximum blood flow velocity envelope), the data under the envelope tends to be overlooked. Thus, this subset of data appears to remain relatively untapped and underutilized. However, key acoustic signatures present within these data are easily processed through the MUSIC platform, allowing for a more complete picture of flow behavior, beyond a single BFV value. Figure S42 demonstrates one such insight whereby the full velocity profile of blood flow and its relative power allow physiological state differentiation; the conventionally extracted envelope only provides a subset of blood flow velocities, ignoring the slower flow components.

When Figure S42 is combined with MUSIC 's audio playback, the datastream provides an accessible and readily interpretable modality for assessing changes in the health of spinal cord microvasculature. This pipeline thereby has the potential to empower healthcare providers to identify subtle differences that signify injury progression or recovery without needing expertise.

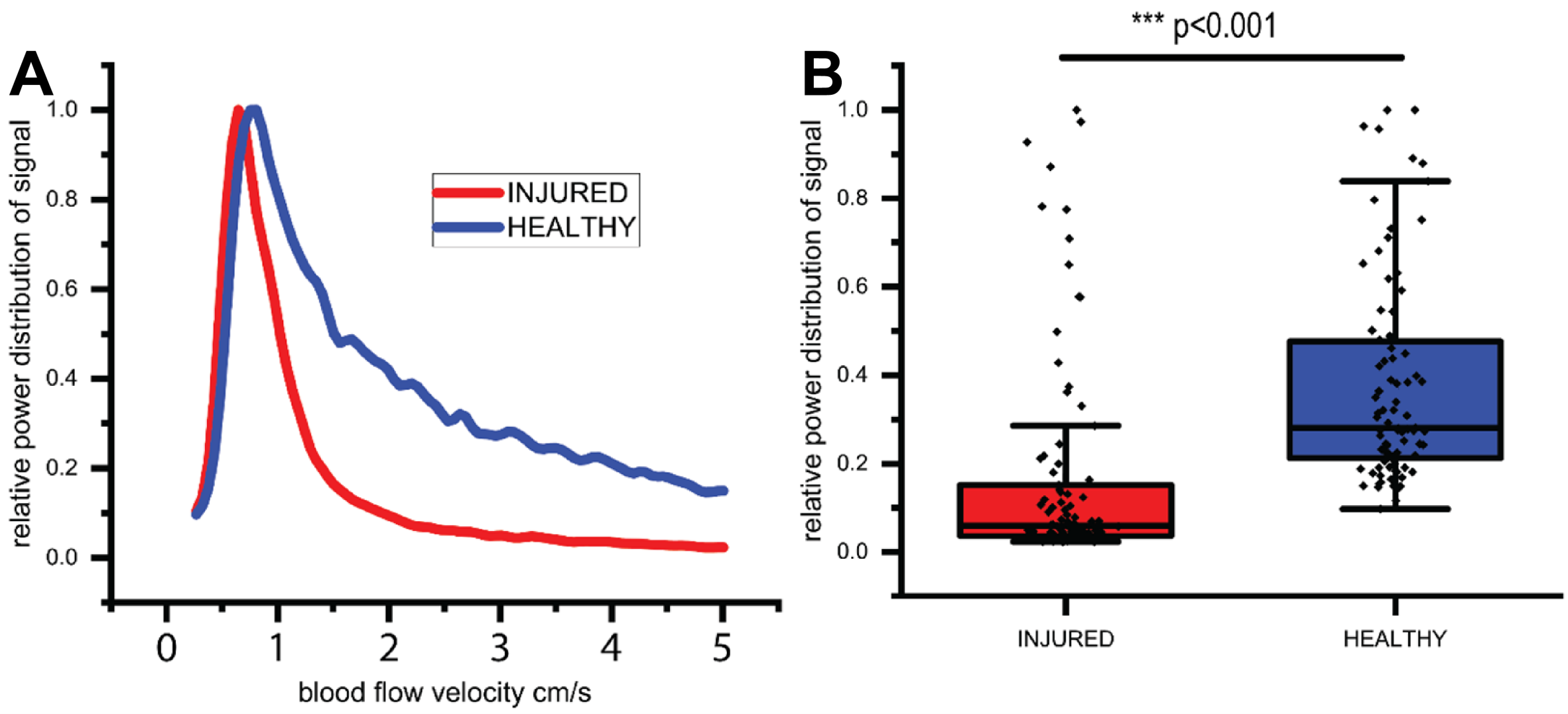

Figure S42: **Hemodynamic profiles of spinal cord microvasculature are plotted with an audio-based adapted power spectral density analysis. (A)** the relative power distribution are plotted for a healthy and an injured blood vessel. **(B)**the data is plotted in 10 Hz (corresponding to 0.05 cm/s) bins across the entire profile. In this instance, the audio system detects a significant difference ($p<0.001$) in the blood flow profile between these states.

Supplementary Section 18
MUSIC's mobile app

The ultrasound neural interface presented in this work addresses critical limitations in managing spinal cord injuries by providing a platform for continuous monitoring and infrastructure for potentially therapeutic interventions. These patients, particularly those recovering post-operatively, could benefit from a reliable means of tracking the injury site to optimize outcomes and minimize complications. Integrating this advanced interface with a corresponding mobile application could provide caregivers with an intuitive and effective way to monitor and manage these complex cases (*84*). Such integration enables access to crucial data, ensuring timely interventions and reducing reliance on additional, intermittent imaging like MRI scans.

The mobile application was developed specifically to use the capabilities of the interface, enabling healthcare providers, for the first time, to monitor local parameters such as the spinal vascular pulsatility index, resistivity index, and microvascular flow after the surgical incision patient is closed. In addition to these local parameters, systemic metrics such as heart rate and respiratory rate can be monitored intermittently for verification and calibration purposes, ensuring optimal device performance. Furthermore, the integration of deep learning-based methods, including hematoma detection and spinal cord segmentation, significantly enhances the app's utility, empowering healthcare providers to identify potential complications with high precision.

This application ensures both real-time and retrospective data viewer and analyzer for healthcare providers. Real-time monitoring facilitates immediate feedback and data visualization, allowing users to assess patient status and interact with the interface for therapeutic intervention, e.g., focused ultrasound stimulation (FUS), when necessary. The app's ability to connect locally via Wi-Fi and Bluetooth or remotely through internet-enabled real-time databases ensures accessibility, regardless of the healthcare providers' location. Moreover, the retrospective monitoring feature enables long-term tracking of patient data, providing valuable insights into the progress of recovery and the efficacy of treatments over time.

The app is developed to analyze and real-time stream B-mode, power Doppler, and spectral Doppler imaging, providing users with comprehensive tools for clinical interpretation. Users can adjust imaging settings such as gain, scale, and focus to optimize image quality. While supporting multiple imaging modes, the app primarily extracts key physiological parameters representing blood flow dynamics from the spectral Doppler waveforms.

Buffers, arrays of data representing a single time point of the spectral Doppler scan, are received in real-time and accumulated into sets of 32 which then undergoes a processing pipeline, including normalization, clipping to a maximum intensity of 0.50 to remove outliers, and de-noising using Gaussian filters (σ=1).

These processed sets, combined with up to 40 previous sets, create a continuous visualization window of approximately 10 seconds for holistic analysis. Advanced techniques like spectral edge detection using threshold-based binary masking identifies waveforms and reconstructs top-edge profiles to extract parameters such as peak systolic velocity (PSV) and end-diastolic velocity (EDV). These values enable the calculation of key metrics, including mean velocity, resistivity index (RI), and pulsatility index (PI), critical for clinical assessment. The processed data, including Base64-encoded spectral images and extracted metrics, is transmitted to the app interface via direct Wi-Fi or Bluetooth connections and stored in Firebase Realtime Database for remote visualization and retrospective analysis(*85*). The app's user interface was built using React Native, ensuring compatibility with both iOS and Android devices, while the backend is implemented in Python to handle all numerical computations efficiently.

Beyond monitoring, the app offers advanced functionalities that streamline patient management and improve care. Detailed reports and notifications keep caregivers informed of significant changes, fostering proactive decision-making. Additionally, the application maintains an exhaustive record of all parameters and interactions, serving as a centralized repository for patient data. This feature not only enhances the caregiver's ability to analyze trends and outcomes but also ensures comprehensive documentation

for following research projects. Together, the ultrasound neural interface and its integrated mobile application represent a transformative approach to spinal cord injury management, marrying cutting-edge technology with user-centric design for optimal care delivery.

A live demonstration of the mobile app on a human with MUSIC as a wearable device placed over the radial artery is shown in **Supplementary Video 11**. The demonstration was approved by the Johns Hopkins Medicine Institutional Review Board (IRB protocol 00353692). The participant provided written informed consent before participation and before use of the resulting images and video. Data were collected in accordance with the approved protocol, and participant privacy was maintained.

Supplementary Section 19

Broader utility of the neural interface including cranial imaging

While MUSIC was designed to monitor and treat spinal cord injury, it also offers significant potential for real-time monitoring of brain ventricles and structures. Specifically, MUSIC has the potential to detect cerebrospinal fluid (CSF) accumulation in conditions such as hydrocephalus—a condition affecting millions worldwide (*86*). Leveraging existing cranial openings, this device could enable on-demand detection of ventricular changes, providing an alternative to repeated imaging with computed tomography or magnetic resonance imaging (*87*). By offering continuous monitoring, MUSIC could reduce unnecessary emergency visits, lower radiation exposure, and significantly cut healthcare costs linked to false alarms. These advantages might be particularly impactful for diagnosing and managing shunt malfunctions, where early detection of ventricular enlargement, infection, or other concerning pathologies can improve long-term neurological outcomes.

A porcine midline craniotomy was performed to evaluate the feasibility of MUSIC for ventricular imaging and assessment of brain anatomy (IACUC approved protocol). This procedure involved creating a triangular, 3-flap stellate incision through the scalp layers (skin, subcutaneous tissue, galea aponeurotica, and periosteum) to expose the skull. Burr holes were created with a perforator drill and connected using a Kerrison rongeur, carefully crossing the superior sagittal sinus. Following the removal of the bone flap, the dura was inspected to ensure its integrity; no durotomy was performed.

Post-craniotomy, MUSIC was placed by hand on the exposed dural surface. *In vivo* B-mode ultrasound imaging of the porcine brain was conducted to visualize gyral folds and ventricular structures. Images acquired using MUSIC were compared with clinical-grade ground truth images captured in a similar manner via a Canon i22LH8 transducer connected to a Canon Aplio i800 ultrasound system (Canon Medical Systems, Tustin, CA, USA). MUSIC demonstrated the capability to delineate the shape of the ventricles and brain anatomy (Figure S43). These results support the feasibility of MUSIC as a portable, nonionizing platform for real-time cranial imaging in this porcine model.

Beyond hydrocephalus, MUSIC has the potential to transform neurological care in a variety of conditions. Its continuous, real-time imaging capabilities could enable brain tumor management by supporting intraoperative navigation, evaluating the extent of resection, and monitoring postoperative recurrence (*88*, *89*). It also paves the way for a platform for ultrasound-based deep brain stimulation to address disorders such as Parkinson's disease and depression and facilitates temporary localized openings of the blood-brain barrier for delivery of targeted therapy (*30*, *90*, *91*). Additionally, MUSIC has long-term implications to enhance stroke management through sonothrombolysis to improve ischemic stroke outcomes and offer insights into brain atrophy in Alzheimer's disease for early intervention. Its continuous monitoring capabilities support timely interventions in traumatic brain injury by assessing structural changes. Furthermore, MUSIC provides an opportunity for enhanced guidance during complex neurosurgical procedures, improving precision and safety. These potential applications illustrate the broader versatility of the platform and motivate future validation across neurological and neurosurgical settings.

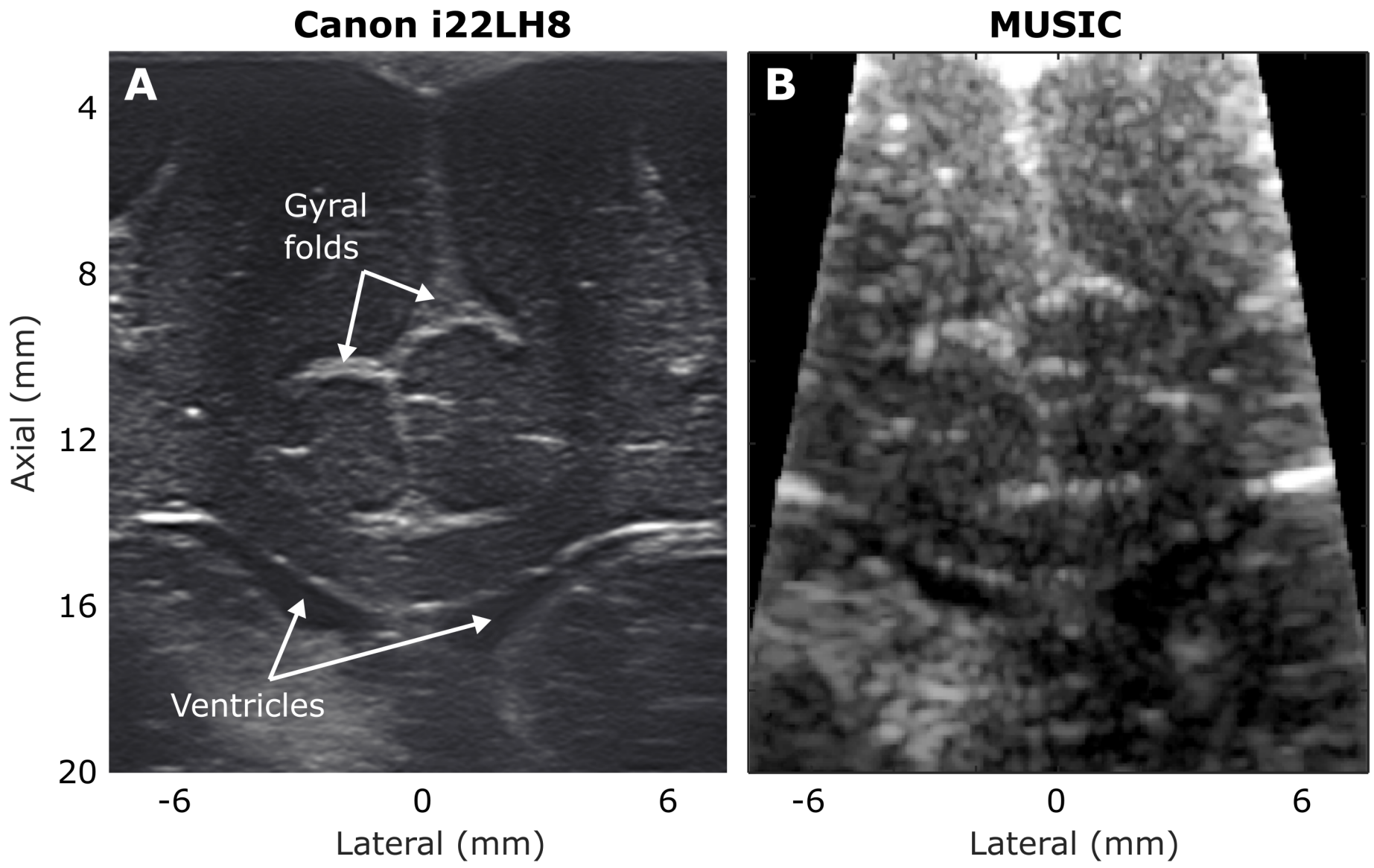

Figure S43: **MUSIC can perform cranial imaging. (A)** B-mode images captured of a porcine brain with the Canon i22LH8 and **(B)** MUSIC transducers. The gyral folds and ventricles are visible in these B-mode images.

Captions for Supplementary Data and Movies

Supplementary Data 1. Source data for the figures. The Excel workbook contains the raw numerical data underlying the main and supplementary figures for which source data are provided. The data are organized in separate worksheets by figure number.

Supplementary Movie 1. Video abstract of the MUSIC ultrasound neural interface. This movie provides an overview of the MUSIC ultrasound neural interface.

Supplementary Movie 2. Automated localization of the spinal cord injury region. The automated hematoma-detection algorithm dynamically localizes the injury region in a porcine spinal cord during intentional changes in orientation.

Supplementary Movie 3. B-mode and power Doppler imaging in a porcine model. High-resolution B-mode and power Doppler imaging acquired with the ultrasound neural interface is shown in a porcine model.

Supplementary Movie 4. Postoperative monitoring in an awake porcine model. An awake pig with MUSIC implanted is shown walking and eating while the interface acquires spinal cord blood-flow velocity data using spectral Doppler.

Supplementary Movie 5. Computer-aided design model of the MUSIC rail system. A computer-aided design model demonstrates the function of the rail system used to safely secure MUSIC.

Supplementary Movie 6. Benchtop demonstration of the MUSIC rail system. The utility and functionality of the custom rail system are demonstrated on the benchtop.

Supplementary Movie 7. Artificial intelligence-assisted spinal cord segmentation. MUSIC compatibility with plug-and-play artificial intelligence models is demonstrated using Segment Anything Model 2 (SAM 2) to highlight the spinal cord in B-mode images during intentional movements and changes in orientation.

Supplementary Movie 8. Intraoperative placement of MUSIC. Placement of MUSIC during porcine surgery is shown at increased playback speed.

Supplementary Movie 9. Spectral Doppler recording in a healthy porcine spinal cord. A localized spectral Doppler blood-flow signal attributable to the sulcal artery region is shown in a healthy porcine spinal cord.

Supplementary Movie 10. Spectral Doppler recording in an injured porcine spinal cord. A localized spectral Doppler blood-flow signal attributable to the sulcal artery region is shown in an injured porcine spinal cord.

Supplementary Movie 11. Mobile application demonstration. An illustrative mobile application is demonstrated using a human radial artery to show the broader utility of MUSIC as a wearable or noninvasive ultrasound device.

REFERENCES

1. GBD 2016 Traumatic Brain Injury and Spinal Cord Injury Collaborators, Global, regional, and national burden of traumatic brain injury and spinal cord injury, 1990–2016: A systematic analysis for the global burden of disease study 2016. *Lancet Neurol.* **18**, 56–87 (2019).

2. R. G. Grossman, R. F. Frankowski, K. D. Burau, E. G. Toups, J. W. Crommett, M. M. Johnson, M. G. Fehlings, C. H. Tator, C. I. Shaffrey, S. J. Harkema, J. E. Hodes, B. Aarabi, M. K. Rosner, J. D. Guest, J. S. Harrop, Incidence and severity of acute complications after spinal cord injury. *J. Neurosurg. Spine* **17**, 119–128 (2012).

3. A. Alizadeh, S. M. Dyck, S. Karimi-Abdolrezaee, Traumatic spinal cord injury: An overview of pathophysiology, models and acute injury mechanisms. *Front. Neurol.* **10**, 282 (2019).

4. B. C. Walters, M. N. Hadley, R. J. Hurlbert, B. Aarabi, S. S. Dhall, D. E. Gelb, M. R. Harrigan, C. J. Rozelle, T. C. Ryken, N. Theodore, American Association of Neurological Surgeons; Congress of Neurological Surgeons, Guidelines for the management of acute cervical spine and spinal cord injuries: 2013 update. *Neurosurgery* **60**, 82–91 (2013).

5. B. K. Kwon, L. A. Tetreault, A. R. Martin, P. M. Arnold, R. A. W. Marco, V. F. J. Newcombe, C. M. Zipser, S. L. McKenna, R. Korupolu, C. J. Neal, R. Saigal, N. E. Glass, S. Douglas, M. Ganau, V. Rahimi-Movaghar, J. S. Harrop, B. Aarabi, J. R. Wilson, N. Evaniew, A. C. Skelly, M. G. Fehlings, A clinical practice guideline for the management of patients with acute spinal cord injury: Recommendations on hemodynamic management. *Global Spine J.* **14**, 187S–211S (2024).

6. T. C. Ryken, R. J. Hurlbert, M. N. Hadley, B. Aarabi, S. S. Dhall, D. E. Gelb, C. J. Rozzelle, N. Theodore, B. C. Walters, The acute cardiopulmonary management of patients with cervical spinal cord injuries. *Neurosurgery* **72**, 84–92 (2013).

7. R. Sajdeya, N. D. Yanez, M. Kampp, M. D. Goodman, D. Zonies, B. Togioka, A. Nunn, R. D. Winfield, N. D. Martin, A. Kohli, T. T. Huynh, D. O. Okonkwo, R. A. Poblete, E. J. Gilmore, R. M. Chesnut, A. E. Bunnell, T. Ohnuma, M. Hashemaghaie, M. M. Treggiari, Early blood

pressure targets in acute spinal cord injury: A randomized clinical trial. *JAMA Netw. Open* **8**, e2525364 (2025).

8. K. M. Kale, S. Patel, S. A. Nischal, L. Ceccon, J. Heller, J. Jallo, J. S. Harrop, S. K. Prasad, Mean arterial pressure augmentation for acute traumatic spinal cord injury: A systematic review and meta-analysis of neurological recovery and mortality. *Global Spine J.* (2026).

9. K. A. Agyeman, D. J. Lee, J. Russin, E. I. Kreydin, W. Choi, A. Abedi, Y. T. Lo, J. Cavaleri, K. Wu, V. R. Edgerton, C. Liu, V. N. Christopoulos, Functional ultrasound imaging of the human spinal cord. *Neuron* **112**, 1710–1722.e3 (2024).

10. Z. Z. Khaing, L. N. Cates, J. E. Hyde, R. Hammond, M. Bruce, C. P. Hofstetter, Transcutaneous contrast-enhanced ultrasound imaging of the posttraumatic spinal cord. *Spinal Cord* **58**, 695–704 (2020).

11. M. J. Kerensky, A. Paul, D. Routkevitch, A. M. Hersh, K. M. Kempski Leadingham, A. D. Davidar, B. F. Judy, J. Punnoose, A. Williams, A. Kumar, K. Lehner, B. Smith, J. K. Son, J. R. Azadi, H. Shekhar, K. P. Mercado-Shekhar, N. V. Thakor, N. Theodore, A. Manbachi, Tethered spinal cord tension assessed via ultrasound elastography in computational and intraoperative human studies. *Commun. Med.* **4**, 4 (2024).

12. C. P. Hofstetter, A. Sadeghi, R. Ramkumar, M. Bruce, Z. Z. Khaing, Sound decisions: Real-time ultrasound in the management of traumatic spinal cord injury. *Curr. Opin. Neurol.* **38**, 635–643 (2025).

13. I. R. Griffiths, J. O. Rowan, R. A. Crawford, Spinal cord blood flow measured by a hydrogen clearance technique. *J. Neurol. Sci.* **26**, 529–544 (1975).

14. S. J. Rothberg, M. S. Allen, P. Castellini, D. Di Maio, J. J. J. Dirckx, D. J. Ewins, B. J. Halkon, P. Muyshondt, N. Paone, T. Ryan, H. Steger, E. P. Tomasini, S. Vanlanduit, J. F. Vignola, An international review of laser Doppler vibrometry: Making light work of vibration measurement. *Opt. Lasers Eng.* **99**, 11–22 (2017).

15. S. Lévy, P.-H. Roche, M. Guye, V. Callot, Feasibility of human spinal cord perfusion mapping using dynamic susceptibility contrast imaging at 7T: Preliminary results and identified guidelines. *Magn. Reson. Med.* **85**, 1183–1194 (2021).

16. J.-S. Tan, C.-C. Lin, G.-S. Chen, Vasomodulation of peripheral blood flow by focused ultrasound potentiates improvement of diabetic neuropathy. *BMJ Open Diabetes Res. Care* **8**, e001004 (2020).

17. Y. Y. Shen, J. V. Jethe, A. P. Reid, J. Hehir, M. M. Amaral, C. Ren, S. Hao, C. Zhou, J. A. N. Fisher, Label free, capillary-scale blood flow mapping in vivo reveals that low-intensity focused ultrasound evokes persistent dilation in cortical microvasculature. *Commun. Biol.* **8**, 12 (2025).

18. M. A. O'Reilly, Exploiting the mechanical effects of ultrasound for noninvasive therapy. *Science* **385**, eadp7206 (2024).

19. H.-J. Kim, T. T. Phan, K. Lee, J. S. Kim, S.-Y. Lee, J. M. Lee, J. Do, D. Lee, S.-P. Kim, K. P. Lee, J. Park, C. J. Lee, J. M. Park, Long-lasting forms of plasticity through patterned ultrasound-induced brainwave entrainment. *Sci. Adv.* **10**, eadk3198 (2024).

20. J. Blesa, J. A. Pineda-Pardo, K.-I. Inoue, C. Gasca-Salas, T. Balzano, N. López-González Del Rey, A. Reinares-Sebastián, N. Esteban-García, R. Rodríguez-Rojas, R. Márquez, M. Ciorraga, M. Del Álamo, L. García-Cañamaque, S. Ruiz de Aguiar, I. Rachmilevitch, I. Trigo-Damas, M. Takada, J. A. Obeso, BBB opening with focused ultrasound in nonhuman primates and Parkinson's disease patients: Targeted AAV vector delivery and PET imaging. *Sci. Adv.* **9**, eadf4888 (2023).

21. Y. Tsehay, Y. Zeng, C. Weber-Levine, T. Awosika, M. Kerensky, A. M. Hersh, Z. Ou, K. Jiang, M. Bhimreddy, S. J. Bauer, J. N. Theodore, V. M. Quiroz, I. Suk, S. Alomari, J. Sun, S. Tong, N. Thakor, J. C. Doloff, N. Theodore, A. Manbachi, Low-intensity pulsed ultrasound neuromodulation of a rodent's spinal cord suppresses motor evoked potentials. *IEEE Trans. Biomed. Eng.* **70**, 1992–2001 (2023).

22. M. Bhimreddy, D. Routkevitch, A. M. Hersh, A. Mohammadabadi, A. K. Menta, K. Jiang, C. Weber-Levine, A. D. Davidar, J. Punnoose, K. M. K. Leadingham, J. C. Doloff, B. Tyler, N. Theodore, A. Manbachi, Disruption of the blood-spinal cord barrier using low-intensity focused ultrasound in a rat model. *J. Vis. Exp.*, e65113, (2023).

23. A. R. Rezai, P. F. D'Haese, V. Finomore, J. Carpenter, M. Ranjan, K. Wilhelmsen, R. I. Mehta, P. Wang, U. Najib, C. V. Ligo Teixeira, T. Arsiwala, A. Tarabishy, P. Tirumalai, D. O. Claassen, S. Hodder, M. W. Haut, Ultrasound blood-brain barrier opening and aducanumab in Alzheimer's disease. *N. Engl. J. Med.* **390**, 55–62 (2024).

24. S. Zhou, G. Park, M. Lin, X. Yang, S. Xu, Wearable ultrasound technology. *Nat. Rev. Bioeng.* **3**, 835–854 (2025).

25. C. Wang, X. Chen, L. Wang, M. Makihata, H.-C. Liu, T. Zhou, X. Zhao, Bioadhesive ultrasound for long-term continuous imaging of diverse organs. *Science* **377**, 517–523 (2022).

26. H. Hu, H. Huang, M. Li, X. Gao, L. Yin, R. Qi, R. S. Wu, X. Chen, Y. Ma, K. Shi, C. Li, T. M. Maus, B. Huang, C. Lu, M. Lin, S. Zhou, Z. Lou, Y. Gu, Y. Chen, Y. Lei, X. Wang, R. Wang, W. Yue, X. Yang, Y. Bian, J. Mu, G. Park, S. Xiang, S. Cai, P. W. Corey, J. Wang, S. Xu, A wearable cardiac ultrasound imager. *Nature* **613**, 667–675 (2023).

27. J. Liu, N. Liu, Y. Xu, M. Wu, H. Zhang, Y. Wang, Y. Yan, A. Hill, R. Song, Z. Xu, M. Park, Y. Wu, J. L. Ciatti, J. Gu, H. Luan, Y. Zhang, T. Yang, H.-Y. Ahn, S. Li, W. Z. Ray, C. K. Franz, M. R. MacEwan, Y. Huang, C. W. Hammill, H. Wang, J. A. Rogers, Bioresorbable shape-adaptive structures for ultrasonic monitoring of deep-tissue homeostasis. *Science* **383**, 1096–1103 (2024).

28. W. Du, L. Zhang, E. Suh, D. Lin, C. Marcus, L. Ozkan, A. Ahuja, S. Fernandez, I. I. Shuvo, D. Sadat, W. Liu, F. Li, A. P. Chandrakasan, T. Ozmen, C. Dagdeviren, Conformable ultrasound breast patch for deep tissue scanning and imaging. *Sci. Adv.* **9**, eadh5325 (2023).

29. E.-Y. Park, X. Cai, J. Foiret, H. Bendjador, D. Hyun, B. Z. Fite, R. Wodnicki, J. J. Dahl, R. D. Boutin, K. W. Ferrara, Fast volumetric ultrasound facilitates high-resolution 3D mapping of tissue compartments. *Sci. Adv.* **9**, eadg8176 (2023).

30. J. F. Hou, M. O. G. Nayeem, K. A. Caplan, E. A. Ruesch, A. Caban-Murillo, E. Criado-Hidalgo, S. B. Ornellas, B. Williams, A. A. Pearce, H. E. Dagdeviren, M. Surets, J. A. White, M. G. Shapiro, F. Wang, S. Ramirez, C. Dagdeviren, An implantable piezoelectric ultrasound stimulator (ImPULS) for deep brain activation. *Nat. Commun.* **15**, 4601 (2024).

31. X. Wan, J. Chen, Ultrasound-induced wireless implantable stimulator for adaptive pain management. *Nat. Electron.* **8**, 384–385 (2025).

32. R. J. Dickinson, R. I. Kitney, Miniature ultrasonic probe construction for minimal access surgery. *Phys. Med. Biol.* **49**, 3527–3538 (2004).

33. M. Hussain, H. Zahra, S. M. Abbas, Y. Zhu, Flexible dielectric materials: Potential and applications in antennas and RF sensors. *Adv. Electron. Mater.* **10**, 2400240 (2024).

34. R. Liang, M. Kerensky, E. Curry, G. Mess, R. Thombre, S. Kamal, F. Aghabaglou, R. Mejia, F. Chavez, K. Morrison, N. Thakor, N. Theodore, A. Manbachi, "Designing an accurate benchtop characterization device: An acoustic measurement platform for localizing and implementing therapeutic ultrasound devices and equipment (amplitude)," in *2022 Design of Medical Devices Conference* (ASME, 2022).

35. A. D. Maxwell, C. A. Cain, T. L. Hall, J. B. Fowlkes, Z. Xu, Probability of cavitation for single ultrasound pulses applied to tissues and tissue-mimicking materials. *Ultrasound Med. Biol.* **39**, 449–465 (2013).

36. T. Şen, O. Tüfekçioğlu, Y. Koza, Mechanical index. *Anatol. J. Cardiol.* **15**, 334–336 (2015).

37. C. Pasquinelli, L. G. Hanson, H. R. Siebner, H. J. Lee, A. Thielscher, Safety of transcranial focused ultrasound stimulation: A systematic review of the state of knowledge from both human and animal studies. *Brain Stimul.* **12**, 1367–1380 (2019).

38. Z. Z. Khaing, J. Leyendecker, J. N. Harmon, S. Sivakanthan, L. N. Cates, J. E. Hyde, M. Krueger, R. W. Glenny, M. Bruce, C. P. Hofstetter, Perfusion imaging metrics after acute traumatic spinal cord injury are associated with injury severity in rats and humans. *Sci. Transl. Med.* **16**, eadn4970 (2024).

39. J. N. Harmon, J. E. Hyde, D. E. Jensen, E. C. D'cessare, A. A. Odarenko, M. F. Bruce, Z. Z. Khaing, Quantifying injury expansion in the cervical spinal cord with intravital ultrafast contrast-enhanced ultrasound imaging. *Exp. Neurol.* **374**, 114681 (2024).

40. M. M. Win, M. Saini, S. D. Pande, K. V. Prasanna, Sulcal artery syndrome presenting as an incomplete Brown-Séquard syndrome: Report of an unusual case and review of the literature. *Proc. Singap. Healthc.* **29**, 59–63 (2020).

41. G. Jocher, A. Chaurasia, J. Qiu, Ultralytics YOLO (2023); https://github.com/ultralytics/ultralytics.

42. N. Ravi, V. Gabeur, Y.-T. Hu, R. Hu, C. Ryali, T. Ma, H. Khedr, R. Rädle, C. Rolland, L. Gustafson, E. Mintun, J. Pan, K. V. Alwala, N. Carion, C.-Y. Wu, R. Girshick, P. Dollár, C. Feichtenhofer, SAM 2: Segment anything in images and videos. arXiv:2408.00714 [cs.CV] (2024).

43. Y.-S. Lee, K.-T. Kim, B. K. Kwon, Hemodynamic management of acute spinal cord injury: A literature review. *Neurospine* **18**, 7–14 (2021).

44. S. D. Wait, M. Y. S. Kalani, A. S. Little, G. D. Consiglieri, J. S. Ross, M. R. Kucia, V. K. H. Sonntag, N. Theodore, Prospective observational study of acute postlumbar laminectomy MRI. *J. Neurosurg. Spine* **20**, 41–44 (2014).

45. C. S. Ahuja, S. Nori, L. Tetreault, J. Wilson, B. Kwon, J. Harrop, D. Choi, M. G. Fehlings, Traumatic spinal cord injury-repair and regeneration. *Neurosurgery* **80**, S9–S22 (2017).

46. P. Herman, A. Stein, K. Gibbs, I. Korsunsky, P. Gregersen, O. Bloom, Persons with chronic spinal cord injury have decreased natural killer cell and increased toll-like receptor/inflammatory gene expression. *J. Neurotrauma* **35**, 1819–1829 (2018).

47. B. K. Kwon, O. Bloom, I.-B. Wanner, A. Curt, J. M. Schwab, J. Fawcett, K. K. Wang, Neurochemical biomarkers in spinal cord injury. *Spinal Cord* **57**, 819–831 (2019).

48. K. M. Kempski Leadingham, D. Routkevitch, A. M. Hersh, M. Kerensky, H. G. Abramson, C. Weber-Levine, K. Robinson, K. Jiang, B. Judy, A. Perdomo-Pantoja, N. Theodore, A. Manbachi, "Quantification of spinal cord microvascular perfusion utilizing ultrasound," in *2023 IEEE International Ultrasonics Symposium (IUS)* (IEEE, 2023), pp. 1–4.

49. C. H. Tegeler, K. Crutchfield, M. Katsnelson, J. Kim, R. Tang, L. Passmore Griffin, T. Rundek, G. Evans, Transcranial Doppler velocities in a large, healthy population. *J. Neuroimaging* **23**, 466–472 (2013).

50. D. Routkevitch, Z. Soulé, N. Kats, E. Baca, A. M. Hersh, K. M. Kempski-Leadingham, A. K. Menta, M. Bhimreddy, K. Jiang, A. D. Davidar, C. Smit, N. Theodore, N. V. Thakor, A. Manbachi, Non-contrast ultrasound image analysis for spatial and temporal distribution of blood flow after spinal cord injury. *Sci. Rep.* **14**, 714 (2024).

51. T. Zhang, N. Pan, Y. Wang, C. Liu, S. Hu, Transcranial focused ultrasound neuromodulation: A review of the excitatory and inhibitory effects on brain activity in human and animals. *Front. Hum. Neurosci.* **15**, 749162 (2021).

52. R. Thombre, G. Mess, K. M. Kempski Leadingham, S. Kapoor, A. Hersh, M. Acord, T. Kaovasia, N. Theodore, B. Tyler, A. Manbachi, Towards standardization of the parameters for opening the blood-brain barrier with focused ultrasound to treat glioblastoma multiforme: A systematic review of the devices, animal models, and therapeutic compounds used in rodent tumor models. *Front. Oncol.* **12**, 1072780 (2023).

53. F. Streijger, K. So, N. Manouchehri, A. Gheorghe, E. B. Okon, R. M. Chan, B. Ng, K. Shortt, M. S. Sekhon, D. E. Griesdale, B. K. Kwon, A direct comparison between norepinephrine and phenylephrine for augmenting spinal cord perfusion in a porcine model of spinal cord injury. *J. Neurotrauma* **35**, 1345–1357 (2018).

54. N. L. Martirosyan, M. Y. S. Kalani, W. D. Bichard, A. A. Baaj, L. F. Gonzalez, M. C. Preul, N. Theodore, Cerebrospinal fluid drainage and induced hypertension improve spinal cord perfusion after acute spinal cord injury in pigs. *Neurosurgery* **76**, 461–469 (2015).

55. Y. G. Kim, S. E. Kim, J. Lee, S. Hwang, S.-S. Yoo, H. W. Lee, Neuromodulation using transcranial focused ultrasound on the bilateral medial prefrontal cortex. *J. Clin. Med.* **11**, 3809 (2022).

56. P. Smith, N. Ogrodnik, J. Satkunarajah, M. A. O’Reilly, Characterization of ultrasound-mediated delivery of trastuzumab to normal and pathologic spinal cord tissue. *Sci. Rep.* **11**, 4412 (2021).

57. S.-M. P. Fletcher, M. Choi, R. Ramesh, M. A. O'Reilly, Focused ultrasound-induced blood–spinal cord barrier opening using short-burst phase-keying exposures in rats: A parameter study. *Ultrasound Med. Biol.* **47**, 1747–1760 (2021).

58. B. Aarabi, J. S. Albrecht, J. M. Simard, T. Chryssikos, G. Schwartzbauer, C. A. Sansur, K. Crandall, M. Gertner, B. Howie, A. Wessell, G. Cannarsa, N. Caffes, J. Oliver, K. Shanmuganathan, J. Olexa, C. Diaz Lomangino, M. Scarboro, Trends in demographics and markers of injury severity in traumatic cervical spinal cord injury. *J. Neurotrauma* **38**, 756–764 (2021).

59. S. Schading-Sassenhausen, D. Pfyffer, L. Farner, A. Grillhoesl, O. Mach, D. Maier, L. Grassner, I. Leister, A. Curt, P. Freund, Extent of traumatic spinal cord injury is lesion level dependent and predictive of recovery: A multicenter neuroimaging study. *J. Neurotrauma* **41**, 2146–2157 (2024).

60. A. M. Moubark, L. Nie, M. H. M. Zaman, M. A. Zulkifley, S. H. M. Ali, S. Freear, Selection of excitation signals and acoustic pressure measurement for in-vivo studies with ultrasound array research platform II. *IOP Conf. Ser.: Mater. Sci. Eng.* **1070**, 012095 (2021).

61. S. Agrawal, K. Johnstonbaugh, J. Y. Clark, J. D. Raman, X. Wang, S.-R. Kothapalli, Design, development, and multi-characterization of an integrated clinical transrectal ultrasound and photoacoustic device for human prostate imaging. *Diagnostics* **10**, 566 (2020).

62. K. M. Kempski, M. T. Graham, M. R. Gubbi, T. Palmer, M. A. Lediju Bell, Application of the generalized contrast-to-noise ratio to assess photoacoustic image quality. Biomedical. *Opt. Express* **11**, 3684–3698 (2020).

63. D. Hyun, A. Wiacek, S. Goudarzi, S. Rothlubbers, A. Asif, K. Eickel, Y. C. Eldar, J. Huang, M. Mischi, H. Rivaz, D. Sinden, R. J. G. van Sloun, H. Strohm, M. A. Lediju Bell, Deep learning for ultrasound image formation: CUBDL evaluation framework and open datasets. *IEEE Trans. Ultrason. Ferroelectr. Freq. Control* **68**, 3466–3483 (2021).

64. A. Rodriguez-Molares, O. M. H. Rindal, J. D'hooge, S.-E. Måsøy, A. Austeng, M. A. L. Bell, H. Torp, The generalized contrast-to-noise ratio: A formal definition for lesion detectability. *IEEE Trans. Ultrason. Ferroelectr. Freq. Control* **67**, 745–759 (2020).

65. L. Steger, A. K. Ghaith, C. Weber-Levine, K. Robinson, C. Krueger, C. Smit, S. Krishnan, K. M. Kempski Leadingham, D. Davidar, D. Routkevitch, K. Jiang, V. Quiroz, S. Bauer, R. Liang, M. Kerensky, I. Suk, B. Tyler, J. C. Doloff, N. Theodore, A. Manbachi, Precision in spinal cord injury research: A novel electromagnetic impactor for a consistent porcine model. *Ann. Biomed. Eng.* **53**, 2983–2995 (2025).

66. C. F. Mullins, S. Harris, D. Pang, A retrospective review of elevated lead impedances in impedance-dependent magnetic resonance-conditional spinal cord stimulation devices. *Pain Pract.* **24**, 270–277 (2024).

67. I. Garg, D. Wang, Complications of spinal cord stimulator trials and implants: A review. *Curr. Pain Headache Rep.* **27**, 837–842 (2023).

68. A. Kumar, K. Kotkar, K. Jiang, M. Bhimreddy, D. Davidar, C. Weber-Levine, S. Krishnan, M. J. Kerensky, R. Liang, K. K. Leadingham, D. Routkevitch, A. M. Hersh, K. Ashayeri, B. Tyler, I. Suk, J. Son, N. Theodore, N. Thakor, A. Manbachi, A novel open-source ultrasound dataset

with deep learning benchmarks for spinal cord injury localization and anatomical segmentation. *Sci. Rep.* **15**, 33192 (2025).

69. Microsoft, Neural Network Intelligence (2021); https://github.com/microsoft/nni.

70. C. H. Tator, I. Koyanagi, Vascular mechanisms in the pathophysiology of human spinal cord injury. *J. Neurosurg.* **86**, 483–492 (1997).

71. J. H. Nederhoed, J. Slikkerveer, K. W. Meyer, W. Wisselink, R. J. P. Musters, K. K. Yeung, Contrast-enhanced sonothrombolysis in a porcine model of acute peripheral arterial thrombosis and prevention of anaphylactic shock. *Lab. Anim* **43**, 91–94 (2014).

72. K. R. Murphy, J. S. Farrell, J. Bendig, A. Mitra, C. Luff, I. A. Stelzer, H. Yamaguchi, C. C. Angelakos, M. Choi, W. Bian, T. DiIanni, E. Martinez Pujol, N. Matosevich, R. Airan, B. Gaudillière, E. E. Konofagou, K. Butts-Pauly, I. Soltesz, L. de Lecea, Optimized ultrasound neuromodulation for non-invasive control of behavior and physiology. *Neuron* **112**, 3252–3266.e5 (2024).

73. R. S. C. Cobbold, *Foundations of Biomedical Ultrasound* (Oxford Univ. Press, 2006).

74. D. Papadopoulos, M. S. Suk, D. Andreychik, V. Nikolaou, M. H. Haak, Rates and causes of reoperations following spinal cord stimulation within a 2-12 year period. *Global Spine J.* **15**, 467–473 (2025).

75. B. C. Hoelzer, M. Bendel, T. Deer, J. S. Eldrige, D. Walega, Z. Wang, S. J. Costandi, G. Azer, W. Qu, S. Falowski, S. A. Neuman, S. Moeschler, C. Wassef, C. K. Kim, T. Niazi, T. Saifullah, B. Yee, C. Kim, C. L. Oryhan, J. Rosenow, D. T. Warren, I. Lerman, R. Mora, S. Hayek, M. C. Hanes, T. T. Simopoulos, S. Sharma, C. Gilligan, W. Grace, T. Ade, N. Mekhail, J. Hunter, D. Choi, D. Y. Choi, Spinal cord stimulator implant infection rates and risk factors: A multicenter retrospective study. *Neuromodulation* **20**, 558–562 (2017).

76. S. Eldabe, E. Buchser, R. Duarte, Complications of spinal cord stimulation and peripheral nerve stimulation techniques: A review of the literature. *Pain Med.* **17**, 325–336 (2016).

77. A. Idbaih, F. Ducray, R. Stupp, N. Baize, O. L. Chinot, J. F. De Groot, J. Guyotat, A. M. Sonabend, P. Menei, H. Dufour, J. Weinberg, C. Desseaux, M. Canney, C. Schmitt, A. Carpentier, A phase I/IIa study to evaluate the safety and efficacy of blood-brain barrier (BBB) opening with the SonoCloud-9 implantable ultrasound device in recurrent glioblastoma patients receiving IV carboplatin. *J. Clin. Oncol.* **39**, 2049 (2021).

78. A. M. Sonabend, A. Gould, Y. Luan, Y. Hou, L. Chen, M. A. Kobayashi, B. A. Castro, D. Y. Zhang, F. V. Korobova, C. Amidei, J. F. Bebawy, B. P. Liu, C. M. Horbinski, C. Desseaux, I. B. Helenowski, H. Zhang, M. M. Muzzio, F. Yue, M. Canney, R. Stupp, Repeated opening of the blood-brain barrier with the skull-implantable SonoCloud-9 (SC9) device: Phase 1 trial of nab-paclitaxel and SC9 in recurrent glioblastoma. *J. Clin. Oncol.* **40**, 2016 (2022).

79. R. Visagan, E. Boseta, A. Zoumprouli, M. C. Papadopoulos, S. Saadoun, Spinal cord perfusion pressure correlates with breathing function in patients with acute, cervical traumatic spinal cord injuries: An observational study. *Crit. Care* **27**, 362 (2023).

80. D. C. Bunten, A. L. Warner, S. R. Brunnemann, J. L. Segal, Heart rate variability is altered following spinal cord injury. *Clin. Auton. Res.* **8**, 329–334 (1998).

81. A. Kimura, A. Seichi, H. Inoue, T. Endo, M. Sato, T. Higashi, Y. Hoshino, Ultrasonographic quantification of spinal cord and dural pulsations during cervical laminoplasty in patients with compressive myelopathy. *Eur. Spine J.* **21**, 2450–2455 (2012).

82. J. Schindelin, I. Arganda-Carreras, E. Frise, V. Kaynig, M. Longair, T. Pietzsch, S. Preibisch, C. Rueden, S. Saalfeld, B. Schmid, J.-Y. Tinevez, D. J. White, V. Hartenstein, K. Eliceiri, P. Tomancak, A. Cardona, Fiji: An open-source platform for biological-image analysis. *Nat. Methods* **9**, 676–682 (2012).

83. D. Ershov, M.-S. Phan, J. W. Pylvänäinen, S. U. Rigaud, L. Le Blanc, A. Charles-Orszag, J. R. W. Conway, R. F. Laine, N. H. Roy, D. Bonazzi, G. Duménil, G. Jacquemet, J.-Y. Tinevez, TrackMate 7: Integrating state-of-the-art segmentation algorithms into tracking pipelines. *Nat. Methods* **19**, 829–832 (2022).

84. S. R. Knight, N. Ng, A. Tsanas, K. Mclean, C. Pagliari, E. M. Harrison, Mobile devices and wearable technology for measuring patient outcomes after surgery: A systematic review. *NPJ Digit. Med.* **4**, 157 (2021).

85. Google Inc., Firebase: Google's mobile and web application development platform (Google, 2024); https://firebase.google.com.

86. NIH: National Institute of Neurological Disorders and Stroke, Hydrocephalus, www.ninds.nih.gov/health-information/disorders/hydrocephalus.

87. T. P. Boyle, L. E. Nigrovic, Radiographic evaluation of pediatric cerebrospinal fluid shunt malfunction in the emergency setting. *Pediatr. Emerg. Care* **31**, 435–440 (2015).

88. R. Sastry, W. L. Bi, S. Pieper, S. Frisken, T. Kapur, W. Wells III, A. J. Golby, Applications of ultrasound in the resection of brain tumors. *J. Neuroimaging* **27**, 5–15 (2017).

89. Y. Yang, X. Hu, Y. Liu, B. Ouyang, J. Zhang, H. Jin, Z. Yu, R. Liu, Z. Li, L. Jiang, X. Lin, B. Xu, An implantable ultrasound-powered device for the treatment of brain cancer using electromagnetic fields. *Sci. Adv.* **8**, eabm5023 (2022).

90. K.-H. Choi, J.-H. Kim, Therapeutic applications of ultrasound in neurological diseases. *J. Neurosonol. Neuroimag.* **11**, 62–72 (2019).

91. A. M. Hersh, M. Bhimreddy, C. Weber-Levine, K. Jiang, S. Alomari, N. Theodore, A. Manbachi, B. M. Tyler, Applications of focused ultrasound for the treatment of glioblastoma: A new frontier. *Cancer* **14**, 4920 (2022).
